# A Photoswitchable HaloTag for Spatiotemporal Control of Fluorescence in Living Cells

**DOI:** 10.1002/anie.202424955

**Published:** 2025-10-23

**Authors:** Franziska Walterspiel, Begoña Ugarte‐Uribe, Jonas Weidenhausen, Merrin Vincent, Kaarjel K. Narayanasamy, Anna Dimitriadi, Arif Ul Maula Khan, Martin Fritsch, Christoph W. Müller, Timo Zimmermann, Claire Deo

**Affiliations:** ^1^ European Molecular Biology Laboratory Meyerhofstraße 1 69117 Heidelberg Germany

**Keywords:** Chemigenetic, Fluorescence microscopy, HaloTag, Photoswitch, Rhodamine

## Abstract

Photosensitive fluorophores, whose emission can be controlled using light, are essential for advanced biological imaging, enabling precise spatiotemporal tracking of molecular features and facilitating super‐resolution microscopy techniques. Although irreversibly photoactivatable fluorophores are well established, reversible reporters that can be reactivated multiple times remain scarce, and only a few have been applied in living cells using generalizable protein labeling methods. To address these limitations, we introduce chemigenetic photoswitchable fluorophores, leveraging the self‐labeling HaloTag protein with fluorogenic rhodamine dye ligands. By incorporating a light‐responsive protein domain into HaloTag, we engineer a tunable, photoswitchable HaloTag (psHaloTag), which can reversibly modulate the fluorescence of a bound dye‐ligand via a light‐induced conformational change. Our best performing psHaloTag variants show excellent performance in living cells, with large, reversible, deep‐red fluorescence turn‐on upon 450 nm illumination across various biomolecular targets and SMLM compatibility. Together, this work establishes the chemigenetic approach as a versatile platform for the design of photoswitchable reporters, tunable through both genetic and synthetic modifications, with promising applications for dynamic imaging.

## Graphical abstract

Photoswitchable fluorophores are critical for advanced bioimaging. Here, we develop a photoswitchable self‐labeling HaloTag that can reversibly modulate the emission of a bound fluorogenic dye via a light‐induced conformational change. In living cells, this tunable chemigenetic system enables precise spatiotemporal activation and control of emitter density in SMLM.

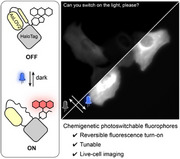

## Introduction

The ability to control the emission properties of a fluorophore using light is a powerful asset for biological imaging.^[^ ^1^, ^2^ ^]^ Indeed, the unique ability of photosensitive reporters to switch between a non‐fluorescent and a fluorescent state under precise light conditions has enabled numerous biological applications, such as the marking and tracking of features of interest with exceptional spatiotemporal control,^[^ ^3^, ^4^, ^5^ ^]^ or the design of multifunctional tools such as light‐gated biosensors.^[^ ^6^, ^7^, ^8^ ^]^ Importantly, photosensitive fluorophores are foundational to super‐resolution microscopy,^[^ ^9^ ^]^ for modalities including SMLM (single molecule localization microscopy),^[^ ^1^, ^10^ ^]^ RESOLFT (reversible saturable optical linear fluorescence transition),^[^ ^11^, ^13^ ^]^ and more recently MINFLUX (minimal photon fluxes),^[^ ^14^, ^15^ ^]^ enabling the visualization of biomolecules below the diffraction limit of light. Generally, photosensitive fluorophores can be categorized in two main groups, depending on whether they undergo an irreversible transformation upon illumination (i.e., photoactivatable, photoconvertible), or a reversible transformation either thermally or upon illumination at a different wavelength (i.e., photoswitchable, photochromic). While many irreversible systems, based either on fluorescent proteins (FPs) or synthetic dye scaffolds, have been developed, photoswitchable systems that can be reactivated multiple times are lacking. To date, most photoswitchable reporters applied in biological samples are based on fluorescent proteins.^[^ ^12^, ^16^, ^17^, ^18^, ^19^, ^20^, ^21^, ^22^ ^]^ However, these inherit the limitations of conventional FPs, with generally low brightness and photostability, particularly in the deep‐red region of the spectrum, high pH sensitivity, and oxygen requirement. Accordingly, synthetic photoswitchable fluorophores are highly attractive, but their engineering has been notoriously difficult. Indeed, Förster Resonance Energy Transfer‐based photoswitchable fluorophores generally display low contrast between the OFF and ON forms, and can present stability issues due to differential bleaching of the donor and acceptor.^[^ ^23^, ^24^, ^25^ ^]^ Intrinsically fluorescent photoswitches such as diarylethenes and spiropyrans show coupled switching and excitation, which limits brightness or switching efficiency.^[^ ^26^, ^27^, ^28^ ^]^ A promising approach is the modulation of the electronic conjugation of workhorse fluorophores, as shown in rhodamine‐lactams,^[^ ^29^, ^30^, ^31^, ^32^ ^]^ in which the open‐close equilibrium can be indirectly affected by light. However, most of these systems require low wavelength light (≤ 405 nm) for activation, and their use in living cells with broadly applicable protein labeling methods remains scarce. Together, this highlights the need for novel approaches for fluorescence photocontrol, combining high brightness, efficient photoswitching using visible light, and live cell compatibility.

**Figure 1:**
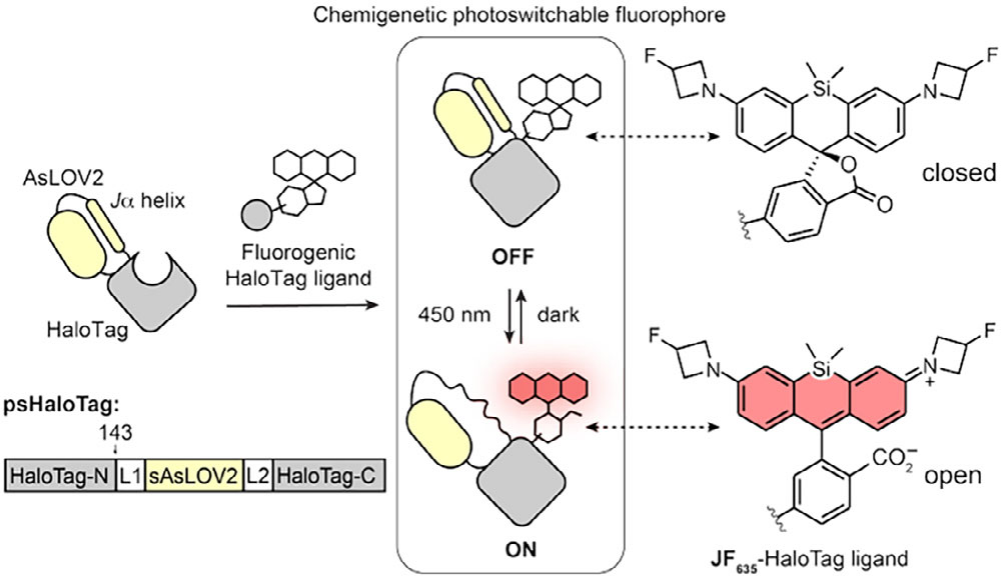
General principle of photoswitchable HaloTag (psHaloTag) and open‐closed equilibrium of **JF_635_**‐HaloTag ligand (HTL). L1 and L2 denote linkers.

Here, we introduce a “chemigenetic” approach for photoswitchable fluorescent systems, leveraging the HaloTag self‐labeling protein and bright fluorogenic rhodamine ligands. Fluorogenic rhodamines predominantly exist in a closed, non‐fluorescent state in solution.^[^ ^33^ ^]^ Upon binding to the HaloTag protein,^[^ ^34^, ^35^ ^]^ the change in dye environment shifts the equilibrium towards the open, fluorescent form, resulting in a large fluorescence turn‐on. This property has been recently exploited for the design of biosensors, based on genetically encoded sensing motifs fused to HaloTag in which an analyte‐dependent conformational change alters the equilibrium and hence the fluorescence of the dye.^[^ ^36^, ^37^, ^38^, ^39^ ^]^ Here, we repurpose this approach to engineer a photoswitchable fluorescent system by integrating a light‐responsive protein domain into the HaloTag protein (Figure 1). Upon illumination, the conformational change of the protein photoswitch alters the dye environment, shifting its equilibrium towards the open, fluorescent state. This yielded a tunable, photoswitchable HaloTag (psHaloTag), which can modulate the fluorescence of a bound ligand in response to light. This system retains the excellent photophysical properties of established rhodamines, with entirely electronically decoupled photoswitching and fluorescence processes. Advantageously, the fluorogenic dye becomes switchable only when bound to the protein tag, guaranteeing low background from unbound reporters. Our best performing psHaloTag variants exhibit robust deep‐red fluorescence turn‐on in vitro as well as in living cells across various subcellular targets with excellent spatiotemporal control, and enable light‐controlled SMLM imaging. Together, this work demonstrates the potential of the chemigenetic approach for fluorescence photocontrol in complex biological samples.

## Results and Discussion

### Engineering of a Photoswitchable HaloTag

To engineer a photoswitchable self‐labeling tag, we set out to introduce a photoswitchable domain into the HaloTag, in close proximity to the dye binding site. We reasoned that the incorporation of the light‐sensing motif into HaloTag would change its interaction with the fluorogenic dye ligand, resulting in low absorption/fluorescence in the dark. Upon illumination, the conformational change of the photo‐responsive domain would subsequently alter the environment of the bound fluorogenic dye, leading to a shift in the open‐closed equilibrium and concomitant absorption and fluorescence increase. As the genetically encoded photoswitch, we selected the light‐oxygen‐voltage 2 domain of *Avena sativa* phototropin 1 (AsLOV2).^[^ ^40^, ^41^ ^]^ This small 16.5 kDa protein uses flavin mononucleotide (FMN) as a cofactor, and comprises two terminal α‐helices, the small N‐terminal A’α and the large C‐terminal Jα helix. Exposure to 450 nm light induces the formation of a metastable photo‐adduct between a cysteine side chain and the FMN cofactor, which leads to structural rearrangements and undocking and unfolding of the Jα helix. This large conformational change has been extensively used to control the activity of effector molecules, and is fundamental for the design of optogenetic tools.^[^ ^40^ ^]^ The photo‐reaction is reversible, with backfolding to the thermally stable state occurring spontaneously in the dark.^[^ ^42^ ^]^

For our design, we used a truncated version of the AsLOV2 domain between residues 408 and 543, termed sAsLOV2, previously shown to be functional with improved conformational coupling when fused to nanobodies,^[^ ^43^, ^44^ ^]^ and added a glycine to each terminus of the domain to provide flexibility. Using the crystal structure of HaloTag bound to tetramethylrhodamine (PDB: 6Y7A),^[^ ^45^ ^]^ six residues on the surface of the HaloTag protein (143, 154, 160, 166, 178, and 180, Figure S1a) were selected for engineering due to their proximity to the bound fluorophore ligand. Accordingly, a series of 16 HaloTag‐sAsLOV2 chimeras was generated, either by insertion of sAsLOV2 into HaloTag or fusion of sAsLOV2 to the termini of a circularly permuted HaloTag (Figure S1b). We selected **JF_635_‐HTL** (λ_max_/λ_em_ = 635/652 nm) as the fluorophore ligand for engineering, due to its large fluorescence turn‐on upon binding to HaloTag (∼113 fold),^[^ ^46^ ^]^ and the success of this dye ligand for engineering sensitive chemigenetic biosensors based on conformational change.^[^ ^36^, ^37^, ^38^, ^39^ ^]^ The different constructs were evaluated in bacterial lysates (Figure S2), and we assessed their ability to bind **JF_635_‐HTL**, and their fluorescence intensity in the dark (Figure S1c,d). Among them, only constructs with HaloTag modified at positions 143 and 154 retained efficient binding to **JF_635_‐HTL** (i.e., near complete binding after 2 h of incubation at room temperature). This is in agreement with previous work, in which these HaloTag regions have been successfully targeted for engineering.^[^ ^36^, ^47^, ^48^ ^]^ Insertion of a flexible SG linker between the HaloTag and LOV domains led to faster binding kinetics for some of the slower‐binding constructs; however, they still did not reach complete binding after 2 h (e.g., at position 180, Figure S1d). The constructs generated from modification at position 154 showed a basal fluorescence in the dark comparable to that of HaloTag labeled with **JF_635_‐HTL**, while constructs generated from modifications at position 143 showed 8 − 35% of HaloTag fluorescence, indicating that the fluorophore is only partially open in those constructs. We then assessed the light‐response of the efficiently‐binding constructs by measuring the change in deep‐red absorption upon photoswitching of the LOV domain, elicited by illumination at 450 nm using a custom‐made LED device (Figure S3). Among them, 3 constructs showed an absorption change, reflecting modulation of the open‐closed equilibrium, all with a small decrease upon illumination at 450 nm, which was fully reversible when incubated in the dark (Figure S1e). Indeed, the insertion of sAsLOV2 after residues 143 (**#2**, Figure S1) or 154 (**#3**) of HaloTag led to A/A_0_ of 0.5 and 0.7, respectively. The terminal fusion of sAsLOV2 to HaloTag circularly permuted at the same positions did not lead to any absorption change. This could be explained by the fact that only one terminus of the LOV domain is attached to the tag in those constructs, resulting in limited conformational coupling. Surprisingly, however, construct **#1** with the sAsLOV2 fused directly at the N‐terminus of the unmodified HaloTag protein showed a small response, with a 10% decrease in absorption upon illumination. This might be due to the relative orientation of the two domains in this construct, which could bring them in close proximity to interact, as previously observed with EGFP‐HaloTag fusions.^[^ ^49^ ^]^ Based on this initial screening, we selected ins143HaloTag‐sAsLOV2 (construct **#2**) for further engineering, showing the largest change in absorption, and advantageously a lower absorption in the dark state, which suggests substantial room for engineering constructs displaying large fluorescence turn‐ons, by reversing the directionality of this scaffold.

To facilitate screening of a large number of mutants, we set out to slow down the thermal recovery. Indeed, the original, “wild‐type” AsLOV2 displays a t_1/2_ ∼1 min, too fast for robust measurements of the ON state properties using steady‐state UV–Vis and fluorescence spectroscopies, and we therefore introduced a point mutation known to slow‐down the thermal relaxation.^[^ ^42^, ^50^ ^]^ The V416L mutation (numbered according to the AsLOV2 sequence) led to an unstable protein; however, V416I led to a construct displaying efficient photoswitching, with a t_1/2_ ∼6.8 min at room temperature. This “slow‐relaxing” construct, ins143HaloTag‐sAsLOV2(V416I), was called psHaloTag0.1, and used as the starting point for improvement.

**Figure 2:**
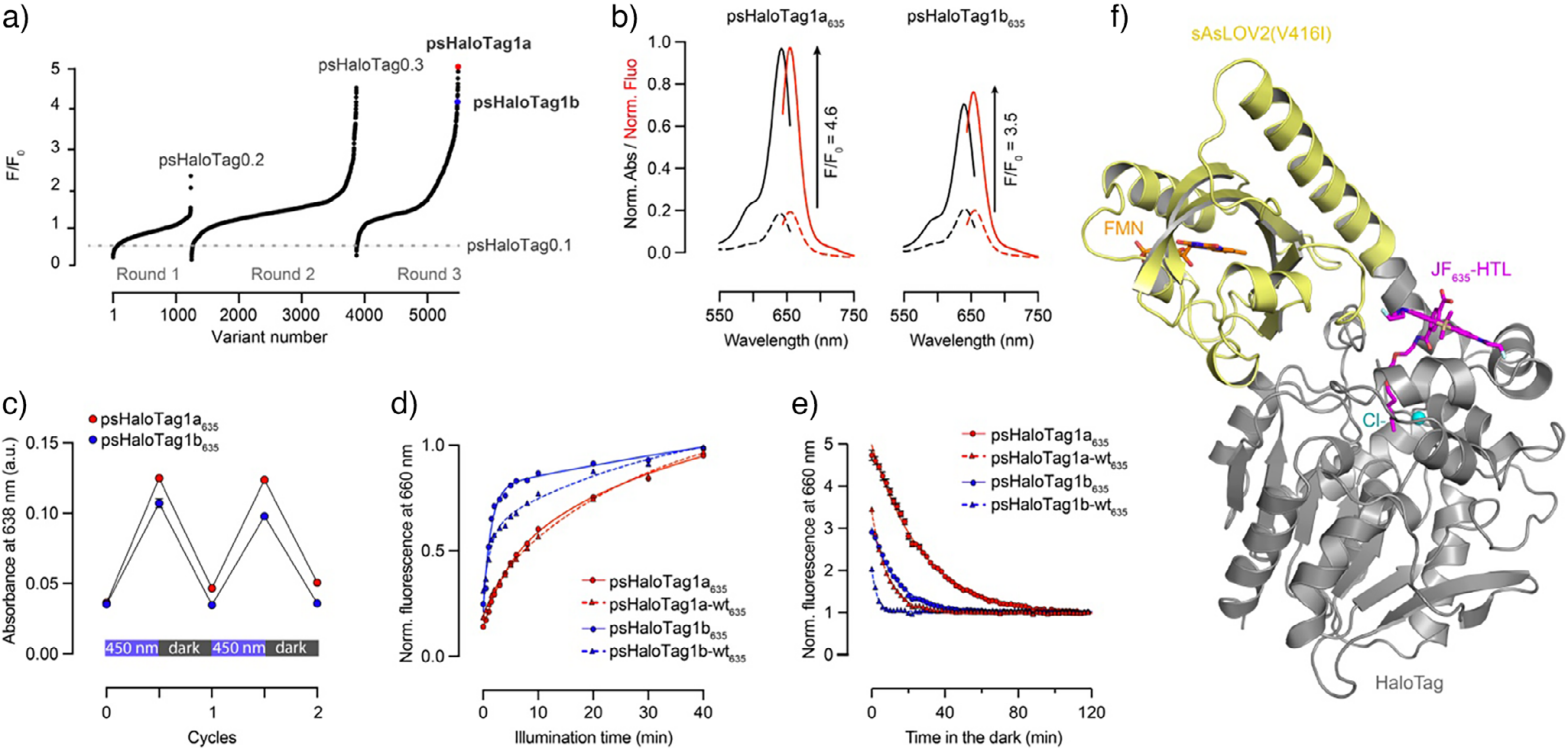
Engineering and characterization of psHaloTag. a) Sequential rounds of site‐saturation mutagenesis with selected mutants highlighted in each round. b) Normalized absorption (black) and fluorescence (red) spectra of psHaloTag1a_635_ ( = protein‐dye conjugate of psHaloTag1a and **JF_635_‐HTL**) and psHaloTag1b_635_ in the dark (dashed lines) and following illumination at 450 nm (solid lines). c) Absorbance at 638 nm of psHaloTag1a_635_ and psHaloTag1b_635_ during two cycles of illumination followed by recovery (dark, room temperature). d) Kinetics of the **JF_635_** fluorescence turn‐on upon illumination at 450 nm for the psHaloTag constructs. e) Kinetics of the **JF_635_** fluorescence turn‐off (dark, room temperature). f) Crystal structure of psHaloTag1a labeled with **JF_635_‐HTL** in the dark state (PDB: 9HKF).

Several rounds of protein engineering were then performed (Figures 2a and S4), with screening in *E. coli* lysates to quantify ligand binding efficiency, fluorescence turn‐on in response to light, and associated photoswitching kinetics (Figure S2). To reverse the response of the system, and therefore lead to a preferable fluorescence turn‐on upon illumination, we first investigated the effect of linker length between HaloTag and sAsLOV2, with a two amino acid linker extension on either side based on the HaloTag sequence (either FA at the N‐term, or PE at the C‐term of the sAsLOV2 domain, respectively, Figure S4). These insertions had no measurable effect on the photoswitching and fluorescence properties, and we therefore performed site‐saturation mutagenesis of selected residues within those linkers. This led to the identification of psHaloTag0.1‐G283P (psHaloTag0.2), which showed a small **JF_635_** fluorescence turn‐on upon illumination. To further increase the turn‐on, we next targeted residues in proximity to the fluorophore binding site, expected to be critical for function (20 amino acids, Figure S4), and performed site‐saturation mutagenesis. We identified mutation E143W, leading to a large improvement of fluorescence turn‐on upon illumination (psHaloTag0.3). An additional round of targeted site‐saturation mutagenesis in residues around the HaloTag surface (11 amino acids) led to two improved mutants, A285W (psHaloTag1a, F/F_0_ = 4.6) and A285L (psHaloTag1b, F/F_0_ = 3.5, Figure 2b). Gratifyingly, both constructs showed full reversibility in the dark (Figure 2c). While psHaloTag1b showed a smaller turn‐on, the photoswitching kinetics were substantially faster than psHaloTag1a, warranting its selection for further investigation (Figure 2d).

### Characterization of psHaloTag in vitro

psHaloTag1a, psHaloTag1b, and the hits isolated in each round of mutagenesis (Figure S4 and Table S1) were fully characterized as purified proteins after labeling with **JF_635_‐HTL** (the protein‐dye conjugates will be hereafter referred to as psHaloTag_635_). We measured their absorption and fluorescence spectra in the dark and after illumination at 450 nm, and recorded the kinetics of the photoactivation and thermal relaxation at room temperature (Table 1 and Figure S5). While psHaloTag0.1_635_ showed an absorption‐driven change in fluorescence (A/A_0_ = 0.4, F/F_0_ = 0.5), psHaloTag0.2_635_ displayed a small absorption turn‐off and fluorescence turn‐on (A/A_0_ = 0.95, F/F_0_ = 1.1). This suggests that, in this construct, the fluorescence modulation stems predominantly from a small change in fluorescence quantum yield, rather than a shift in open‐close equilibrium. Introduction of mutation E143W in psHaloTag0.3_635_ resulted in very different properties, recovering an equilibrium‐driven mechanism (A/A_0_ = 3.1, F/F_0_ = 3.4). This was retained in improved mutants psHaloTag1a_635_ (A/A_0_ = 4.7, F/F_0_ = 4.6) and psHaloTag1b_635_ (A/A_0_ = 3.1, F/F_0_ = 3.5). We confirmed that psHaloTag1a displayed fast binding to **JF_635_‐HTL** in the dark, with labeling complete in 15 min (Figure S6). psHaloTag1a_635_ showed low pH sensitivity over the range of pH 6−9 (Figure S7), similarly to other HaloTag/rhodamine‐based chemigenetic platforms.^[^ ^38^, ^51^ ^]^ Both psHaloTag1a_635_ and psHaloTag1b_635_ showed high Φ_F _>0.68, and displayed ∼40% of the brightness of HaloTag_635_ in their ON states due to lower extinction coefficients, which demonstrates that the dye does not fully open in those constructs. Although further improvements are certainly possible to shift the dye further towards the open form in the ON state, it is worth noting that psHaloTag1a_635_ is substantially brighter than other red‐ and deep‐red‐emitting photoswitchable proteins, e.g., 3‐fold brighter than PSmOrange (*λ*_max_/*λ*_em_ = 634/662 nm)^[^ ^19^ ^]^ and rsFusionred1 (λ_max_/λ_em_ = 577/605 nm),^[^ ^13^ ^]^ and 16‐fold brighter than rsCherry (*λ*_max_/*λ*_em_ = 572/610 nm).^[^ ^21^ ^]^

**Table 1:** Sequence information and properties of the psHaloTag variants labeled with **JF_635_‐HTL**. Wavelengths, extinction coefficients, and quantum yields correspond to the ON state of the constructs, measured after illumination at 450 nm. t_1/2, relax_ corresponds to the half‐life of the thermal relaxation of the **JF_635_** signal (measured in the dark at room temperature). n.m.: not measured.

Construct	L1	L2	mutations	*λ*_max_/*λ*_em_ (nm)	ε (M^−1^.cm^−1^)	Φ_Fl_	A/A_0_	F/F_0_	t_1/2,relax_ (min)
HaloTag	/	/	/	640/656	81 000	0.75	1	1	/
psHaloTag0.1	G	G	/	641/656	12 800	0.70	0.4	0.5	6.8
psHaloTag0.2	FAG	P	/	641/656	46 300	0.75	0.95	1.1	n.m.
psHaloTag0.3	FAG	P	E143W	640/654	30 900	0.76	3.1	3.4	12.6
psHaloTag1a	FAG	P	E143W, A285W	642/655	38 200	0.68	4.7	4.6	19.5
psHaloTag1a‐wt	FAG	P	E143W, A285W, I155V	641/656	25 200	n.m.	3.6	4.4	5.9
psHaloTag1b	FAG	P	E143W, A285L	639/655	30 200	0.74	3.1	3.5	9.7
psHaloTag1b‐wt	FAG	P	E143W, A285L, I155V	639/655	20 900	n.m.	2.4	3.1	1.8

In order to assess the composition of the ON state of psHaloTag1a_635_ and psHaloTag1b_635_, we generated “dark” and “lit” mimics of these constructs, introducing known mutations in the AsLOV2 domain (“dark mimic”: C189A, “lit mimic”: I271E, A275E in psHaloTag1a/1b, Figure S8).^[^ ^52^, ^53^ ^]^ Spectroscopic evaluation revealed that the dark mimic closely resembles the dark state of the parent constructs. The lit mimic shows slightly higher intensity in both absorption and fluorescence, with a small hypsochromic shift. While using these “mimics” to replicate the two extreme cases of the photocycle is an approximation, this suggests that the OFF state corresponds to ∼100% of folded proteins, and the ON state contains >85% of unfolded proteins for both psHaloTag1a and psHaloTag1b, therefore supporting efficient photoswitching.

We then examined the photoswitching kinetics of the different constructs, measuring absorption and fluorescence of both the FMN cofactor (λ_max_/λ_em_ = 450/525 nm) and **JF_635_** (λ_max_/λ_em_ = 640/660 nm) (Figure S5 and Table S2). Under illumination at 450 nm, the FMN absorption decreases, due to the formation of the covalent bond with the cysteine residue in the LOV scaffold, while the absorption of **JF_635_** increases as the Jα helix unfolds, which elicits a conformational change in the HaloTag protein close to the dye. The LOV‐FMN adduct formation occurred rapidly in all constructs, reaching a photostationary state after the first illumination. In contrast, the **JF_635_** turn‐on was substantially slower. This suggests a two‐steps process for fluorescence activation, which could be explained as the deep‐red fluorescence modulation arises from a conformational change that needs to be propagated from the Jα helix to the HaloTag, and likely requires important rearrangements while outcompeting the thermal relaxation process. This was also confirmed by light intensity titration, which shows two different regimes (Figure S9). At low light intensity (<10 µW⋅cm^−2^ here), the performance of the system is reduced due to incomplete LOV‐FMN photocycle. In contrast, the kinetics and dynamic range become completely intensity‐independent above a certain threshold (100 µW⋅cm^−2^), when the second phase of the fluorescence turn‐on becomes rate‐limiting. This infers that, above a certain threshold, the photoswitching kinetics become independent on light power and protein concentration, which constitutes an attractive feature of the system in the context of quantitative studies. psHaloTag1b_635_ showed ∼6‐fold faster turn‐on of the deep‐red signal than psHaloTag1a_635_. Similar observations were made for the thermal relaxation kinetics where psHaloTag1a_635_ and 1b_635_ showed similar t_1/2,relax_(FMN) of 6.7 and 7.9 min, but distinct deep‐red kinetics with t_1/2,relax_(JF_635_) of 19.5 and 9.7 min, respectively. The difference could be explained by the presence of the additional bulky tryptophane W285 at the hinge region in construct 1a, possibly slowing‐down conformational rearrangement. The larger F/F_0_ measured for 1a could be the direct result of the slower thermal relaxation, and could additionally stem from differences in the unfolded protein structure between 1a and 1b, shifting the equilibrium of the dye to different extents. Interestingly, the initial negative variant psHaloTag0.1_635_ showed a different behavior, with similar t_1/2,relax_ for both the FMN and **JF_635_** signals. These differences suggest that the conformational rearrangement of the protein scaffold is the rate‐limiting step in the fluorescence turn‐on of the system. To investigate this further, we reversed the initial “slow” mutation introduced in psHaloTag0.1 for screening purposes, leading to the “wild‐type” psHaloTag1a‐wt (psHaloTag1a(I155V), with I155V in the psHaloTag sequence corresponding to I416V in AsLOV2 sequence numbering) and psHaloTag1b‐wt (psHaloTag1b(I155V)) mutants. Both retained fluorescence turn‐on, albeit slightly smaller than the parent constructs with F/F_0_ of 4.4 and 3.1 for psHaloTag1a‐wt and psHaloTag1b‐wt, respectively. This could be explained by the competing faster thermal relaxation (Table 1, Table S2). Indeed, the t_1/2,ON_(JF_635_) were unchanged compared to the parent constructs, but the turn‐off kinetics were, as expected, substantially faster, with t_1/2_,_relax_(FMN) = 0.6 min for both constructs and t_1/2_,_relax_(JF_635_) = 5.9 and 1.8 min for psHaloTag1a‐wt_635_ and psHaloTag1b‐wt_635_, respectively (Figure 2d,e). While the deep‐red fluorescence modulation remains slower than the FMN photocycle, this demonstrates that faster kinetics can be achieved, with minimal impact on the ON/OFF contrast. Overall, t_1/2_,_relax_(FMN) values are conserved across psHaloTag variants containing the same AsLOV2 mutant (Table S2), and are in the same range as the reported values for the LOV domain alone (13 min for AsLOV2(V416I), 0.9 min for AsLOV2‐wt),^[^ ^42^, ^50^ ^]^ suggesting that HaloTag minimally impacts the FMN photocycle.

**Figure 3:**
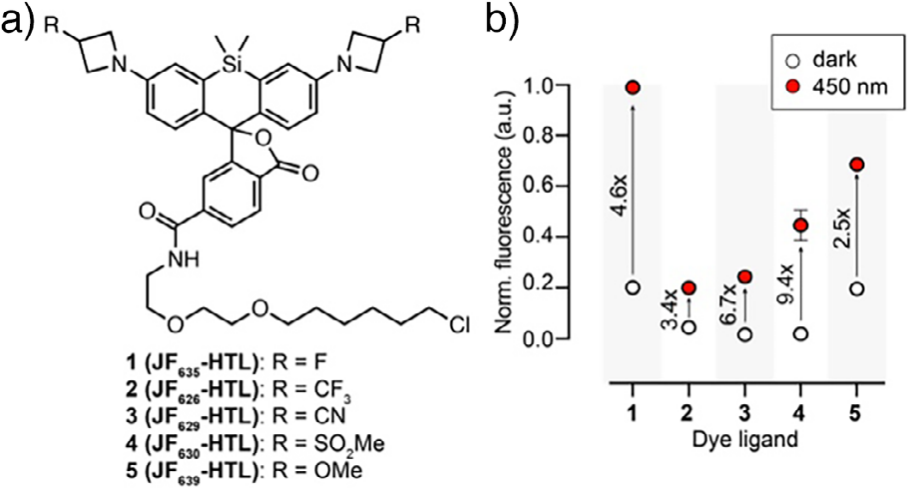
a) Chemical structure of Si‐rhodamine ligands. b) Normalized fluorescence in the OFF (white dots) and ON (red dots) states for psHaloTag1a labeled with dyes **1 − 5**. Numbers on the graph indicate F/F_0_. Fluorescence was normalized to the ON state of psHaloTag1a_635_.

A key property of chemigenetic systems is the high tunability afforded by modifying the dye ligand.^[^ ^54^ ^]^ Indeed, using a different fluorogenic HaloTag ligand can lead to a broad range of spectral properties, dynamic ranges and kinetics, while generally retaining function. We therefore tested a panel of dyes in combination with psHaloTag1a, including fluorogenic Janelia Fluor (JF) dyes and Max‐Planck (MaP) dyes (Figure S10).^[^ ^36^, ^46^, ^55^, ^56^, ^57^ ^]^ All dyes tested led to a fluorescence turn‐on upon illumination, albeit to different extent. The MaP dyes, as well as the O─, C─, and P─ bridged rhodamines, led to smaller turn‐ons, with F/F_0_ ≤ 2.0, whereas the other Si‐rhodamines, closely resembling **JF_635_**, led to substantially higher turn‐ons. In this dye series (compounds **1 − 5**, Figures 3 and S11), the trend followed the electron‐withdrawing capability of the azetidine substituents, and hence the open‐close equilibrium of the dyes, with dyes more closed generally resulting in lower fluorescence intensities in both the ON and OFF states. Both **JF_629_‐HTL** and **JF_630_‐HTL** led to larger fluorescence turn‐ons of 6.7 and 9.4 folds, respectively. Among compounds **1 − 5**, the kinetics were strongly dye‐dependent, with larger ON/OFF fluorescence ratios generally correlating with slower turn‐on kinetics, while the relaxation kinetics were varied with no particular trend. These results demonstrate that the psHaloTag system can be finely tuned by changing the dye ligand, with adjustable brightness, turn‐on ratio and kinetics, while retaining the advantageous deep‐red spectral features.

### Structure of psHaloTag

To gain insights into the mechanism of psHaloTag, we used AlphaFold for structure prediction.^[^ ^58^, ^59^ ^]^ The predicted structures indicate that the Jα helix is fused to one of the helices of HaloTag, resulting in an elongated helix (Figure S12). This structural feature may contribute to the mechanism, where illumination‐induced Jα‐helix unfolding is propagated to residues near the fluorophore ligand. The predicted structures of psHaloTag1a and 1b appear similar, with a slightly different bent in the extended helix. However, AlphaFold predicts uncertainty in these linker regions, which makes it difficult to unambiguously conclude on structural differences. In addition, structure prediction does not provide information on the position and state of the dye ligand or FMN cofactor. We therefore solved the crystal structure of psHaloTag1a bound to **JF_635_‐HTL**, which diffracted to 2.4 Å resolution (PDB: 9HKF), allowing us to place the ligands in the electron density. The crystals formed in space group C121 and contained two assemblies per asymmetric unit. The structure was determined by molecular replacement using a HaloTag crystal structure (PDB: 5Y2Y) as an initial model, followed by several cycles of building and refining. The two psHaloTag1a molecules adopt a near‐identical conformation with a root‐mean‐square deviation (RMSD) of 0.33 Å (for 431 Cα residues) (Figure S13a,b). The extended Jα helix is in a folded state in both molecules and consists of about 1/3 HaloTag and 2/3 LOV domain residues. Our experimentally determined structure is also in agreement with the AlphaFold prediction, with an RMSD of 1.64Å (for 371 Cα residues) (Figure S13c). The major difference between the structure and the prediction is attributed to a slightly different bend of the Jα helix, resulting in a shifted orientation of the LOV domain. The FMN cofactors, **JF_635_‐HTL** fluorophore ligands, and Cl^−^ ions were clearly visible in the electron density. Based on the density, both FMN molecules appear to be unbound to the adjacent C189 residues, and the folded helix of the LOV domain suggests crystallization of the OFF state (Figure S14). **JF_635_‐HTL** adopted the same position as analogous fluorophore ligands on HaloTag (Figure S14b–d), and while the electron density around the dyes does not allow for unambiguous identification of the open or closed conformation, the open conformation resulted in the best fit (Figure S13d). While we would expect a primarily closed conformation in solution for the OFF state, the observed open form could be explained by the tight crystal packing around the fluorophore (Figure S13a), as the surface‐exposed dyes from the two chains face each other in the center of the asymmetric unit. The two performance‐increasing mutations E143W and A285W are in proximity to each other, and A285W to the headgroup of the **JF_635_‐HTL** fluorophore. These tryptophane residues could form an extended hydrophobic network together with neighboring hydrophobic residues (I150, L147, and additional residues towards the center of the protein), which could improve the stabilization/interaction of the fluorophore and improve performance. Overall, both the predicted and the crystal structures show that the HaloTag and LOV domains are tightly arranged and connected by a long helix, which ensures efficient propagation of the conformational change to the HaloTag in close proximity to the fluorophore.

### Characterization in Living Cells

**Figure 4:**
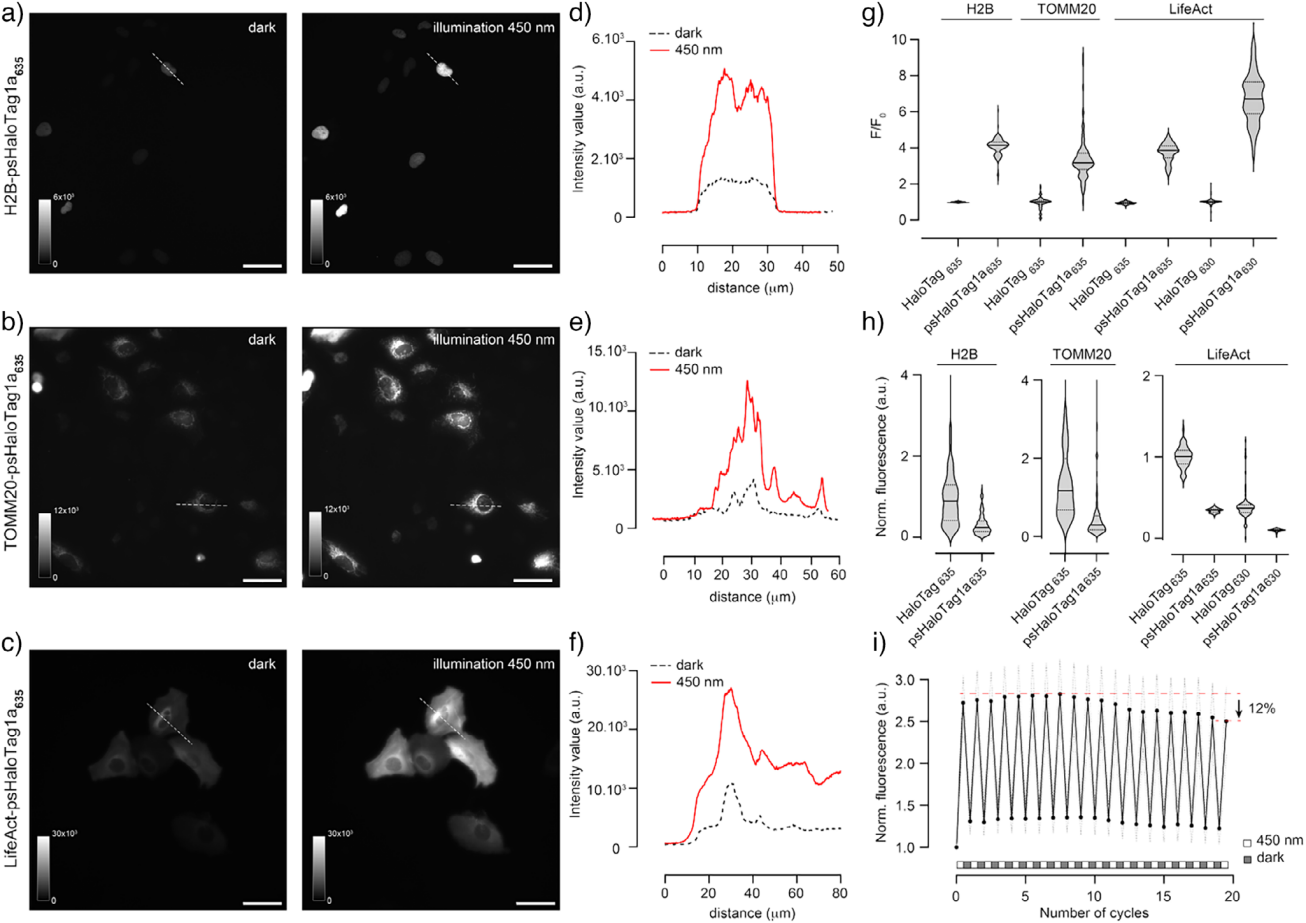
Characterization of psHaloTag1a in living cells. a–c) Representative images of U2OS cells expressing psHaloTag1a‐T2A‐EGFP targeted to the nucleus (a), mitochondria (b), or actin (c) and labeled with **JF_635_‐HTL**, in the dark and after illumination at 450 nm. Scale bars: 50 µm. d–f). Intensity line profiles corresponding to the images in (a–c), respectively. g) F/F_0_ for HaloTag and psHaloTag1a targeted to different cellular locations. h) Normalized fluorescence intensity for the same cells as in g). Fluorescence intensity is the ratio of deep‐red to EGFP fluorescence, and was normalized to the value of HaloTag_635_ for each respective localization. i) Normalized fluorescence intensity of cells expressing H2B‐psHaloTag1a and labeled with **JF_635_‐HTL**, upon cycles of illumination at 450 nm followed by incubation in the dark. To reduce measurement time, the dark incubation was shorter than for full recovery. Quantification in panels g–h). was performed on N>100 cells for each condition.

Next, we investigated the performance of psHaloTag1a in living cells to confirm that photoswitching was retained in the native cellular environment and that the system was broadly usable across various subcellular locations. U2OS cells expressing psHaloTag1a‐T2A‐EGFP targeted to the nucleus (H2B), mitochondria (TOMM20), or F‐actin (LifeAct) were stained with **JF_635_‐HTL** (1 µM, 2 h), washed, and imaged using widefield microscopy (Figure 4). Specific labeling of psHaloTag1a was confirmed by the dim deep‐red fluorescence in the OFF state, showing excellent colocalization with established cellular stains (Figure S15). No cell death due to cytotoxicity or phototoxicity was observed when cells were subjected to extended illumination (Figure S16).

Gratifyingly, a large fluorescence turn‐on was observed for all targets after illumination, while the HaloTag control showed no fluorescence change (Figure 4a–g). The dynamic range in cells was very consistent across subcellular targets, with F/F_0_ between 3.5 − 4.1 for psHaloTag1a_635_ (Figure 4g), and about 30% of the brightness of HaloTag_635_ (Figure 4h), in excellent alignment with the in vitro measurements. This supports that psHaloTag displays low sensitivity for the surrounding cellular environment. As expected, **JF_630_‐HTL** led to a significantly larger turn‐on, with F/F_0 _= 7.0 for LifeAct‐psHaloTag1a_630_ (Figure 4g, Figure S17). In the dark, the system was fully reversible (Figure S17c), and showed good fatigue resistance, with only a 12% decrease in performance for H2B‐psHaloTag1a_635_ after 20 cycles (Figure 4i, Movie S1).

**Figure 5:**
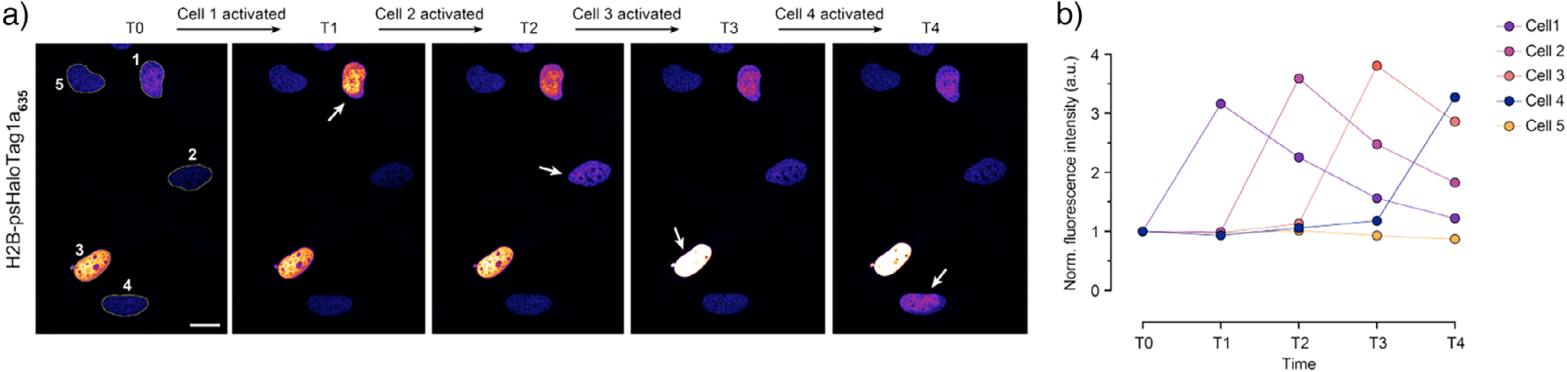
Sequential photoactivation of single cells. a) Representative confocal images of living U2OS cells expressing H2B‐psHaloTag1a and labeled with **JF_635_‐HTL** in the dark (T_0_) and consecutive single‐cell photoactivation at 450 nm for 6 min (T_1_‐T_4_). In each frame, the activated cell is indicated by a white arrow. Scale bar: 20 µm. b) Corresponding fluorescence intensity of individual cells. Each curve corresponds to one cell with corresponding numbering in frame T_0_.

Importantly, psHaloTag enables spatially and temporally defined fluorescence turn‐on of labeled targets in living cells. Living U2OS cells expressing H2B‐psHaloTag1a and labeled with **JF_635_‐HTL** were imaged by confocal microscopy, and spatially‐restricted photoactivation was performed using the microscope's 450 nm laser beam. Activation could be performed with excellent spatial and temporal control, evidenced by large fluorescence turn‐on of individual cells (Figures 5 and S18). The reversibility of the system is clearly visible, with the fluorescence of activated cells gradually decreasing over subsequent frames, demonstrating transient marking of subcellular features inside living cells.

### Single Molecule Imaging in Living Cells

**Figure 6:**
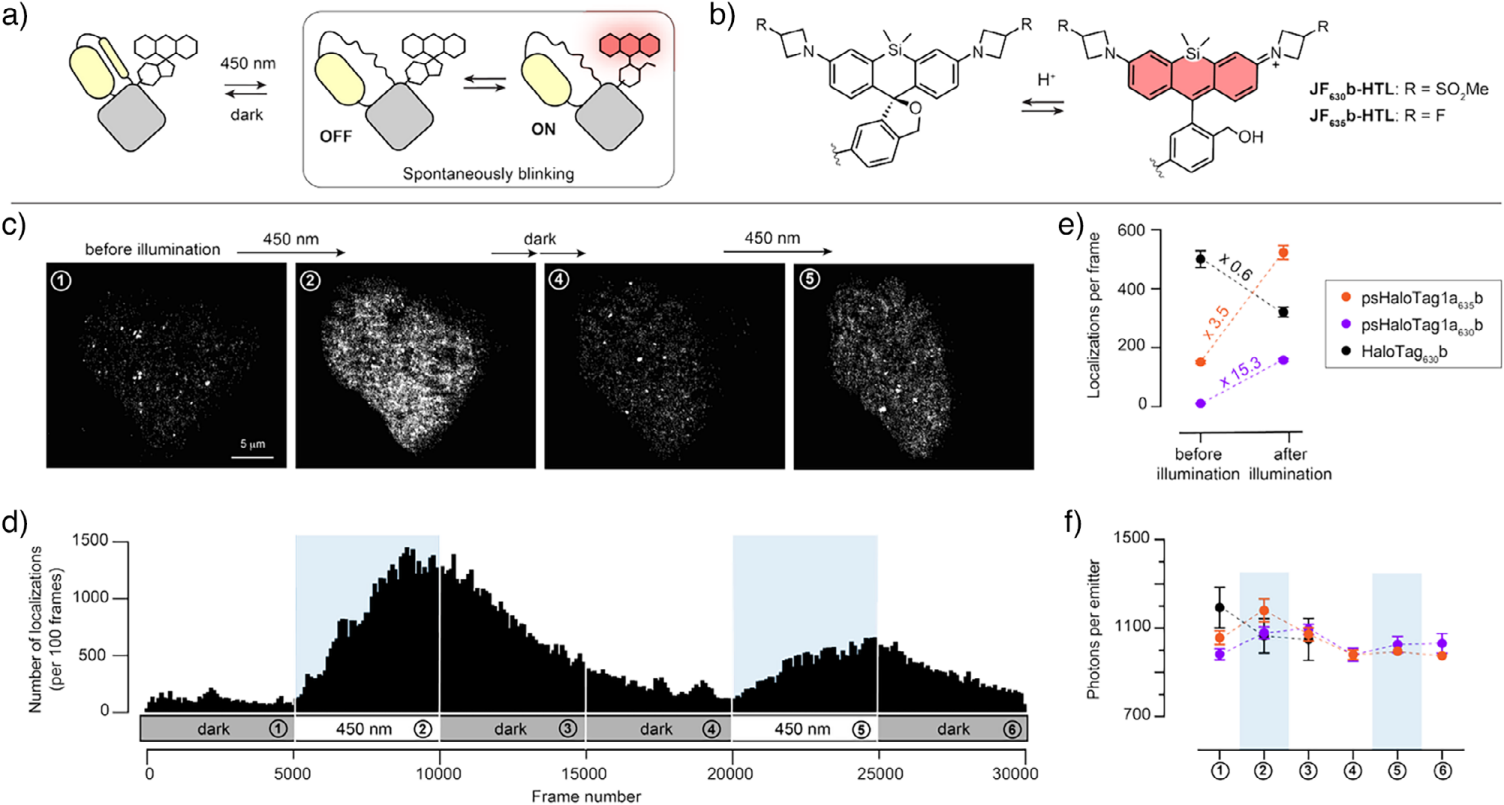
Single‐molecule localization microscopy. a) General principle of controllable blinking using psHaloTag and self‐blinking dyes. b) Structure and blinking of hydroxymethyl‐rhodamines **JF_630_b‐HTL** and **JF_635_b‐HTL**. c) Representative example of SMLM images of live U2OS cells expressing H2B‐psHaloTag1a and labeled with **JF_630_b‐HTL** (25 nM) before illumination, during illumination at 450 nm, after thermal recovery, and during a second illumination at 450 nm. Each reconstructed image was rendered from 5000 frames, acquired at 100 ms per frame. d) Number of localizations per 100 frames for the cell in panel c. during cycles of illumination/dark. e) Number of localizations per frame before and after illumination in live U2OS cells expressing psHaloTag1a or HaloTag labeled with **JF_630_b‐HTL** or **JF_635_b‐HTL**. f) Corresponding number of photons per localization during the acquisition. N = 4–6 individual cells from 3–4 independent samples, error bars indicate ± SEM.

Finally, we sought to demonstrate the potential of psHaloTag for single‐molecule imaging. Light‐responsive fluorophores are essential components of super‐resolution microscopy methods such as single‐molecule localization microscopy (SMLM), where photoactivation induces blinking events that can be localized to reconstruct images beyond the diffraction limit of light.^[^ ^1^ ^]^ The quality of SMLM images depends on obtaining isolated emitters, but at the same time, high enough emitter densities reduce the notoriously long acquisition time in SMLM imaging, which is especially pertinent for live cell imaging. Emitter densities of transiently binding fluorophores such as DNA‐PAINT^[^ ^60^ ^]^ and exchangeable HaloTag ligands^[^ ^61^ ^]^ can be optimized by varying dye concentration; however, the control of emitter densities of bound fluorophores is challenging. We reasoned that our system could be used to precisely control the blinking behavior of bound fluorophores upon illumination, thereby regulating the density of single‐molecule emitters over time (Figure 6a).^[^ ^30^, ^62^ ^]^ To this end, we focused on spontaneously blinking hydroxymethyl‐rhodamines, which undergo stochastic interconversion between open and closed forms (Figure 6b).^[^ ^55^, ^63^, ^64^, ^65^, ^66^ ^]^ These fluorophores enable SMLM imaging in living cells without the need for specialized buffers or illumination; however, their blinking behavior is uncontrollable, limiting broad applicability. Notably, the blinking behavior of such dyes is directly influenced by the protein environment of self‐labeling tags.^[^ ^65^ ^]^ Thus, photoswitching of psHaloTag should alter the environment surrounding the hydroxymethyl‐rhodamine ligand, enabling controlled modulation of its blinking kinetics. To confirm this hypothesis, we labeled living U2OS cells expressing H2B‐psHaloTag1a with the spontaneously blinking dyes **JF_630_b‐HTL** or **JF_635_b‐HTL** (Figure 6b).^[^ ^66^ ^]^ We performed SMLM using a 640 nm excitation laser and 450 nm photoactivation illumination. Briefly, 5000 frame movies (100 ms per frame) were recorded under 640 nm excitation for each illumination condition: 1) no activation, 2) 450 nm activation, then 3)&4) two recordings of the recovery phase without activation, 5) second 450 nm activation, and finally 6) second recovery. In the OFF state, psHaloTag1a_630_b displayed a very low number of emitters per frame (Figures 6c,d, and S19). Illumination at 450 nm led to a 15.3‐fold increase in localizations per frame (Figure 6e). This unambiguously demonstrates that psHaloTag can substantially modulate the blinking dynamics of bound fluorophores. In the dark, thermal relaxation and photobleaching lead to a gradual decrease in the number of emitters, and the system could be further reactivated, albeit to a smaller extent, likely due to cumulative bleaching over these extended acquisition times. In comparison, psHaloTag1a_635_b showed a substantially higher number of emitters in the dark state, and faster photobleaching (Figures 6e and S20). The turn‐on in the number of localizations was less pronounced (3.5‐fold, Figure 6e), but faster than for **JF_630_b‐HTL**, consistent with the trends observed with the parent non‐blinking dyes in vitro. In contrast, HaloTag showed no light‐response, and photobleaching was observed exclusively (Figure S21). The number of photons per emitter was light‐independent and comparable across dye: protein pairs (Figure 6f), suggesting that the modulation mainly affects kinetics and activation potential, while dye brightness is conserved.

Together, these results show that psHaloTag enables fine control over single‐molecule emitter density, a promising approach for adapting to diverse samples and targets. Moreover, the relatively slow ON and OFF kinetics of the system can be beneficial in this context, allowing precise dosage of emitter numbers with illumination duration, while the slow thermal relaxation ensures that only temporally sparse activation is required. While further studies will be needed to establish the full capabilities of psHaloTag, our findings demonstrate its strong potential for single‐molecule imaging in living cells.

## Conclusion

In this work, we developed a photoswitchable HaloTag (psHaloTag) that enables light‐controlled reversible fluorescence modulation of a deep‐red rhodamine fluorophore through a light‐driven conformational change. We systematically explored the insertion of a light‐sensitive AsLOV2 domain into the HaloTag protein, and improved the performance of the system through rationally guided site‐saturation mutagenesis. The psHaloTag platform leverages the superior properties of synthetic dyes, such as their high brightness, while conferring them a light‐responsive behavior. The system was largely tunable, and dynamic range, photoswitching, and thermal relaxation kinetics could be finely modified by introducing point mutations or varying the dye ligand. The best performing psHaloTag‐fluorophore combination shows close to 10‐fold fluorescence turn‐on in vitro, and is substantially brighter than existing red‐shifted photoswitchable proteins. We demonstrated that this system works robustly in living cells across multiple subcellular targets, showing reversible fluorescence turn‐on, with high spatiotemporal control. In addition, we demonstrate the usability of this system for SMLM, where the density of single‐molecule emitters can be precisely controlled via protein photoswitching.

Looking forward, we expect that continued directed evolution and further structure‐based engineering will further improve fluorescence turn‐on ratios and photoswitching kinetics. Furthermore, this first‐generation psHaloTag is exclusively thermally reversible, which imposes limitations for applications requiring rapid activation and reversibility. Future iterations of the platform could incorporate optically reversible light‐sensitive domains, enabling more versatile spatiotemporal control. Together, this work represents a step forward in integrating synthetic dyes and protein scaffolds for fluorescence photoswitching, with the potential to open new avenues in dynamic imaging and super‐resolution microscopy.

## Supporting Information

Supplementary figures and tables, materials, and methods (pdf).

Supplementary movie S1.

The crystal structure of psHaloTag1a labeled with **JF_635_‐HTL** has been deposited in the Protein Data Bank (PDB: 9HKF).

Plasmid pCoofy‐psHaloTag1a is available through Addgene (#232940).

The image analysis code used for segmentation and intensity quantification, along with test data, can be found at (CBA / psHaloTag-mitochondria-intensity-quantification · GitLab).

SMLM data, including 5000‐frame raw movies and localized files from Picasso for the datasets used in Figures 6, S19, S20, and S21, are available on Zenodo (https://zenodo.org/records/16941576).

## Conflict of Interests

The authors declare no conflict of interest.

## Supporting information



Supporting Information

Supporting Information

## Data Availability

The data that support the findings of this study are available from the corresponding author upon reasonable request.

## References

[1] F. M. Jradi , L. D. Lavis , ACS Chem. Biol. 2019, 14, 1077–1090, 10.1021/acschembio.9b00197. 30997987

[2] M. Olesińska‐Mönch , C. Deo , Chem. Commun. (Camb) 2023, 59, 660–669. 36622788 10.1039/d2cc05870g

[3] I. Nemet , P. Ropelewski , Y. Imanishi , Photochem. Photobiol. Sci. 2015, 14, 1787–1806, 10.1039/c5pp00174a. 26345171 PMC4589530

[4] F. Pennacchietti , J. Alvelid , R. A. Morales , M. Damenti , D. Ollech , O. S. Oliinyk , D. M. Shcherbakova , E. J. Villablanca , V. V. Verkhusha , I. Testa , Nat. Commun. 2023, 14, 8402, 10.1038/s41467-023-44054-9. 38114484 PMC10730883

[5] D. M. Chudakov , V. V. Verkhusha , D. B. Staroverov , E. A. Souslova , S. Lukyanov , K. A. Lukyanov , Nat. Biotechnol. 2004, 22, 1435–1439, 10.1038/nbt1025. 15502815

[6] Z. Zou , Z. Luo , X. Xu , S. Yang , Z. Qing , J. Liu , R. Yang , TrAC, Trends Anal. Chem. 2020, 125.

[7] K. Mishra , J. P. Fuenzalida‐Werner , F. Pennacchietti , R. Janowski , A. Chmyrov , Y. Huang , C. Zakian , U. Klemm , I. Testa , D. Niessing , V. Ntziachristos , A. C. Stiel , Nat. Biotechnol. 2022, 40, 598–605, 10.1038/s41587-021-01100-5. 34845372 PMC9005348

[8] X. Chai , H. H. Han , A. C. Sedgwick , N. Li , Y. Zang , T. D. James , J. Zhang , X. L. Hu , Y. Yu , Y. Li , Y. Wang , J. Li , X. P. He , H. Tian , J. Am. Chem. Soc. 2020, 142, 18005–18013, 10.1021/jacs.0c05379. 32955867

[9] T. J. Chozinski , L. A. Gagnon , J. C. Vaughan , FEBS Lett. 2014, 588, 3603–3612, 10.1016/j.febslet.2014.06.043. 25010263

[10] K. Kikuchi , L. D. Adair , J. Lin , E. J. New , A. Kaur , Angew. Chem. Int. Ed. Engl. 2023, 62, e202204745, 10.1002/anie.202204745. 36177530 PMC10100239

[11] J. Kwon , J. Hwang , J. Park , G. R. Han , K. Y. Han , S. K. Kim , Sci. Rep. 2015, 5, 17804, 10.1038/srep17804. 26639557 PMC4671063

[12] T. Grotjohann , I. Testa , M. Reuss , T. Brakemann , C. Eggeling , S. W. Hell , S. Jakobs , Elife 2012, 1, e00248. 23330067 10.7554/eLife.00248 PMC3534202

[13] F. Pennacchietti , E. O. Serebrovskaya , A. R. Faro , Shemyakina, II, N. G. Bozhanova , A. A. Kotlobay , N. G. Gurskaya , A. Boden , J. Dreier , D. M. Chudakov , K. A. Lukyanov , V. V. Verkhusha , A. S. Mishin , I. Testa , Nat. Methods 2018, 15, 601–604, 10.1038/s41592-018-0052-9. 29988095

[14] A. Aktalay , T. A. Khan , M. L. Bossi , V. N. Belov , S. W. Hell , Angew. Chem. Int. Ed. Engl. 2023, 62, e202302781, 10.1002/anie.202302781. 37555720

[15] R. Lincoln , M. L. Bossi , M. Remmel , E. D'Este , A. N. Butkevich , S. W. Hell , Nat. Chem. 2022, 14, 1013–1020, 10.1038/s41557-022-00995-0. 35864152 PMC9417988

[16] T. Brakemann , A. C. Stiel , G. Weber , M. Andresen , I. Testa , T. Grotjohann , M. Leutenegger , U. Plessmann , H. Urlaub , C. Eggeling , M. C. Wahl , S. W. Hell , S. Jakobs , Nat. Biotechnol. 2011, 29, 942–947, 10.1038/nbt.1952. 21909082

[17] R. Ando , H. Hama , M. Yamamoto‐Hino , H. Mizuno , A. Miyawaki , Proc. Natl. Acad. Sci. USA 2002, 99, 12651–12656. 12271129 10.1073/pnas.202320599 PMC130515

[18] M. Andresen , A. C. Stiel , J. Folling , D. Wenzel , A. Schonle , A. Egner , C. Eggeling , S. W. Hell , S. Jakobs , Nat. Biotechnol. 2008, 26, 1035–1040, 10.1038/nbt.1493. 18724362

[19] O. M. Subach , G. H. Patterson , L. M. Ting , Y. Wang , J. S. Condeelis , V. V. Verkhusha , Nat. Methods 2011, 8, 771–777, 10.1038/nmeth.1664. 21804536 PMC3164916

[20] F. V. Subach , L. Zhang , T. W. Gadella , N. G. Gurskaya , K. A. Lukyanov , V. V. Verkhusha , Chem. Biol. 2010, 17, 745–755, 10.1016/j.chembiol.2010.05.022. 20659687 PMC2911641

[21] A. C. Stiel , M. Andresen , H. Bock , M. Hilbert , J. Schilde , A. Schonle , C. Eggeling , A. Egner , S. W. Hell , S. Jakobs , Biophys. J. 2008, 95, 2989–2997, 10.1529/biophysj.108.130146. 18658221 PMC2527278

[22] C. Gregor , S. C. Sidenstein , M. Andresen , S. J. Sahl , J. G. Danzl , S. W. Hell , Sci. Rep. 2018, 8, 2724, 10.1038/s41598-018-19947-1. 29426833 PMC5807511

[23] K. Torii , S. Benson , Y. Hori , M. Vendrell , K. Kikuchi , Chem. Sci. 2024, 15, 1393–1401, 10.1039/D3SC04953A. 38274070 PMC10806661

[24] Y. Xiong , P. Rivera‐Fuentes , E. Sezgin , A. Vargas Jentzsch , C. Eggeling , H. L. Anderson , Org. Lett. 2016, 18, 3666–3669, 10.1021/acs.orglett.6b01717. 27456166 PMC5010358

[25] N. Soh , K. Yoshida , H. Nakajima , K. Nakano , T. Imato , T. Fukaminato , M. Irie , Chem. Commun. (Camb) 2007, 5206, 10.1039/b713663c. 18060143

[26] K. Uno , M. L. Bossi , T. Konen , V. N. Belov , M. Irie , S. W. Hell , Adv. Opt. Mater. 2019, 7.

[27] K. Uno , M. L. Bossi , M. Irie , V. N. Belov , S. W. Hell , J. Am. Chem. Soc. 2019, 141, 16471–16478, 10.1021/jacs.9b08748. 31542923

[28] L. Wu , Y. Dai , X. Jiang , C. Petchprayoon , J. E. Lee , T. Jiang , Y. Yan , G. Marriott , PLoS One 2013, 8, e64738, 10.1371/journal.pone.0064738. 23755140 PMC3674008

[29] F. M. Jradi , B. P. English , T. A. Brown , J. Aaron , S. Khuon , J. A. Galbraith , C. G. Galbraith , L. D. Lavis , bioRxiv 2024, 10.1101/2024.05.12.593749.

[30] A. Eordogh , A. Martin , P. Rivera‐Fuentes , Chemistry 2022, 28, e202202832. 36125781 10.1002/chem.202202832 PMC10092635

[31] Q. Qi , W. Chi , Y. Li , Q. Qiao , J. Chen , L. Miao , Y. Zhang , J. Li , W. Ji , T. Xu , X. Liu , J. Yoon , Z. Xu , Chem. Sci. 2019, 10, 4914–4922, 10.1039/C9SC01284B. 31160962 PMC6510312

[32] J. Folling , V. Belov , R. Kunetsky , R. Medda , A. Schonle , A. Egner , C. Eggeling , M. Bossi , S. W. Hell , Angew. Chem. Int. Ed. Engl. 2007, 46, 6266–6270, 10.1002/anie.200702167. 17640007

[33] D. Si , Q. Li , Y. Bao , J. Zhang , L. Wang , Angew. Chem. Int. Ed. Engl. 2023, 62, e202307641, 10.1002/anie.202307641. 37483077

[34] L. P. Encell , R. Friedman Ohana , K. Zimmerman , P. Otto , G. Vidugiris , M. G. Wood , G. V. Los , M. G. McDougall , C. Zimprich , N. Karassina , R. D. Learish , R. Hurst , J. Hartnett , S. Wheeler , P. Stecha , J. English , K. Zhao , J. Mendez , H. A. Benink , N. Murphy , D. L. Daniels , M. R. Slater , M. Urh , A. Darzins , D. H. Klaubert , R. F. Bulleit , K. V. Wood , Curr. Chem. Genomics 2012, 6, 55–71, 10.2174/1875397301206010055. 23248739 PMC3520037

[35] A. Cook , F. Walterspiel , C. Deo , ChemBioChem 2023, 24, e202300022. 36815462 10.1002/cbic.202300022

[36] C. Deo , A. S. Abdelfattah , H. K. Bhargava , A. J. Berro , N. Falco , H. Farrants , B. Moeyaert , M. Chupanova , L. D. Lavis , E. R. Schreiter , Nat. Chem. Biol. 2021, 17, 718–723, 10.1038/s41589-021-00775-w. 33795886

[37] M. S. Frei , S. A. Sanchez , X. He , L. Liu , F. Schneider , Z. Wang , H. Hakozaki , Y. Li , A. C. Lyons , T. V. Rohm , J. M. Olefsky , L. Shi , J. Schoneberg , S. E. Fraser , S. Mehta , Y. Wang , J. Zhang , Nat. Biotechnol. 2025, 10.1038/s41587-025-02642-8. PMC13193679 40258957

[38] D. Cheng , Z. Ouyang , X. He , Y. Nasu , Y. Wen , T. Terai , R. E. Campbell , J. Am. Chem. Soc. 2024, 146, 35117–35128, 10.1021/jacs.4c10917. 39601449

[39] J. D. Lee , A. Nguyen , Z. R. Jin , A. Moghadasi , C. E. Gibbs , S. J. Wait , K. M. Evitts , A. Asencio , S. B. Bremner , S. Zuniga , V. Chavan , A. Williams , N. Smith , M. Regnier , J. E. Young , D. Mack , E. Nance , P. M. Boyle , A. Berndt , bioRxiv 2024 10.1101/2024.02.06.579232.

[40] S. P. Zimmerman , B. Kuhlman , H. Yumerefendi , Methods Enzymol 2016, 580, 169–190. 27586333 10.1016/bs.mie.2016.05.058 PMC5369018

[41] S. M. Harper , L. C. Neil , K. H. Gardner , Science 2003, 301, 1541–1544, 10.1126/science.1086810. 12970567

[42] B. D. Zoltowski , B. Vaccaro , B. R. Crane , Nat. Chem. Biol. 2009, 5, 827–834, 10.1038/nchembio.210. 19718042 PMC2865183

[43] A. A. Gil , C. Carrasco‐Lopez , L. Zhu , E. M. Zhao , P. T. Ravindran , M. Z. Wilson , A. G. Goglia , J. L. Avalos , J. E. Toettcher , Nat. Commun. 2020, 11, 4044, 10.1038/s41467-020-17836-8. 32792536 PMC7426870

[44] C. Carrasco‐López , E. M. Zhao , A. A. Gil , N. Alam , J. E. Toettcher , J. L. Avalos , Nat. Commun. 2020, 11, 4045, 10.1038/s41467-020-17837-7. 32792484 PMC7427095

[45] J. Wilhelm , S. Kuhn , M. Tarnawski , G. Gotthard , J. Tunnermann , T. Tanzer , J. Karpenko , N. Mertes , L. Xue , U. Uhrig , J. Reinstein , J. Hiblot , K. Johnsson , Biochemistry 2021, 60, 2560–2575, 10.1021/acs.biochem.1c00258. 34339177 PMC8388125

[46] J. B. Grimm , A. K. Muthusamy , Y. Liang , T. A. Brown , W. C. Lemon , R. Patel , R. Lu , J. J. Macklin , P. J. Keller , N. Ji , L. D. Lavis , Nat. Methods 2017, 14, 987–994, 10.1038/nmeth.4403. 28869757 PMC5621985

[47] M. C. Huppertz , J. Wilhelm , V. Grenier , M. W. Schneider , T. Falt , N. Porzberg , D. Hausmann , D. C. Hoffmann , L. Hai , M. Tarnawski , G. Pino , K. Slanchev , I. Kolb , C. Acuna , L. M. Fenk , H. Baier , J. Hiblot , K. Johnsson , Science 2024, 383, 890–897, 10.1126/science.adg0812. 38386755

[48] W. Zhu , S. Takeuchi , S. Imai , T. Terada , T. Ueda , Y. Nasu , T. Terai , R. E. Campbell , Nat. Chem. Biol. 2023, 19, 38–44, 10.1038/s41589-022-01134-z. 36138142

[49] L. Hellweg , A. Edenhofer , L. Barck , M. C. Huppertz , M. S. Frei , M. Tarnawski , A. Bergner , B. Koch , K. Johnsson , J. Hiblot , Nat. Chem. Biol. 2023, 19, 1147–1157, 10.1038/s41589-023-01350-1. 37291200 PMC10449634

[50] F. Kawano , Y. Aono , H. Suzuki , M. Sato , PLoS One 2013, 8, e82693, 10.1371/journal.pone.0082693. 24367542 PMC3867380

[51] A. Cook , N. Kaydanov , B. Ugarte‐Uribe , J. C. Boffi , G. B. Kamm , R. Prevedel , C. Deo , J. Am. Chem. Soc. 2024, 146, 23963–23971, 10.1021/jacs.4c07080. 39158696 PMC11363013

[52] S. M. Harper , J. M. Christie , K. H. Gardner , Biochemistry 2004, 43, 16184–16192, 10.1021/bi048092i. 15610012

[53] M. Salomon , J. M. Christie , E. Knieb , U. Lempert , W. R. Briggs , Biochemistry 2000, 39, 9401–9410, 10.1021/bi000585+. 10924135

[54] K. K. Tsao , S. Imai , M. Chang , S. Hario , T. Terai , R. E. Campbell , Cell Chem. Biol. 2024, 31, 1652–1664, 10.1016/j.chembiol.2024.08.002. 39236713 PMC11466441

[55] Q. Zheng , A. X. Ayala , I. Chung , A. V. Weigel , A. Ranjan , N. Falco , J. B. Grimm , A. N. Tkachuk , C. Wu , J. Lippincott‐Schwartz , R. H. Singer , L. D. Lavis , ACS Cent. Sci. 2019, 5, 1602–1613, 10.1021/acscentsci.9b00676. 31572787 PMC6764213

[56] J. B. Grimm , A. N. Tkachuk , L. Xie , H. Choi , B. Mohar , N. Falco , K. Schaefer , R. Patel , Q. Zheng , Z. Liu , J. Lippincott‐Schwartz , T. A. Brown , L. D. Lavis , Nat. Methods 2020, 17, 815–821, 10.1038/s41592-020-0909-6. 32719532 PMC7396317

[57] L. Wang , M. Tran , E. D'Este , J. Roberti , B. Koch , L. Xue , K. Johnsson , Nat. Chem. 2020, 12, 165–172, 10.1038/s41557-019-0371-1. 31792385

[58] J. Jumper , R. Evans , A. Pritzel , T. Green , M. Figurnov , O. Ronneberger , K. Tunyasuvunakool , R. Bates , A. Zidek , A. Potapenko , A. Bridgland , C. Meyer , S. A. A. Kohl , A. J. Ballard , A. Cowie , B. Romera‐Paredes , S. Nikolov , R. Jain , J. Adler , T. Back , S. Petersen , D. Reiman , E. Clancy , M. Zielinski , M. Steinegger , M. Pacholska , T. Berghammer , S. Bodenstein , D. Silver , O. Vinyals , et al., Nature 2021, 596, 583–589, 10.1038/s41586-021-03819-2. 34265844 PMC8371605

[59] M. Mirdita , K. Schütze , Y. Moriwaki , L. Heo , S. Ovchinnikov , M. Steinegger , Nat. Methods 2022, 19, 679–682, 10.1038/s41592-022-01488-1. 35637307 PMC9184281

[60] R. Jungmann , C. Steinhauer , M. Scheible , A. Kuzyk , P. Tinnefeld , F. C. Simmel , Nano Lett. 2010, 10, 4756–4761, 10.1021/nl103427w. 20957983

[61] J. Kompa , J. Bruins , M. Glogger , J. Wilhelm , M. S. Frei , M. Tarnawski , E. D'Este , M. Heilemann , J. Hiblot , K. Johnsson , J. Am. Chem. Soc. 2023, 145, 3075–3083, 10.1021/jacs.2c11969. 36716211 PMC9912333

[62] E. A. Halabi , D. Pinotsi , P. Rivera‐Fuentes , Nat. Commun. 2019, 10, 1232, 10.1038/s41467-019-09217-7. 30874551 PMC6420572

[63] S. N. Uno , M. Kamiya , T. Yoshihara , K. Sugawara , K. Okabe , M. C. Tarhan , H. Fujita , T. Funatsu , Y. Okada , S. Tobita , Y. Urano , Nat. Chem. 2014, 6, 681–689, 10.1038/nchem.2002. 25054937

[64] S. N. Uno , M. Kamiya , A. Morozumi , Y. Urano , Chem. Commun. (Camb) 2017, 54, 102–105, 10.1039/C7CC07783A. 29214255

[65] R. Tachibana , M. Kamiya , A. Morozumi , Y. Miyazaki , H. Fujioka , A. Nanjo , R. Kojima , T. Komatsu , T. Ueno , K. Hanaoka , T. Yoshihara , S. Tobita , Y. Urano , Chem. Commun. 2020, 56, 13173–13176, 10.1039/D0CC05126H. 33020769

[66] K. L. Holland , S. E. Plutkis , T. A. Daugird , A. Sau , J. B. Grimm , B. P. English , Q. Zheng , S. Dave , F. Rahman , L. Xie , P. Dong , A. N. Tkachuk , T. A. Brown , R. H. Singer , Z. Liu , C. G. Galbraith , S. M. Musser , W. R. Legant , L. D. Lavis , bioRxiv preprint 2024, 10.1101/2024.02.23.581625.

