# Supplementary material for: A Photoswitchable HaloTag for Spatiotemporal Control of Fluorescence in Living Cells
Source: Angew Chem Int Ed Engl. 2025 Oct 23;64(50):e202424955. doi: 10.1002/anie.202424955 (PMC12684303; doi:10.1002/anie.202424955)
Supplement: Supplementary file 1 — Supporting Information [file ANIE-64-e202424955-s002.pdf]

SUPPLEMENTARY INFORMATION

A photoswitchable HaloTag for spatiotemporal control of fluorescence in living cells

Franziska Walterspiel◊, Begoña Ugarte-Uribe◊, Jonas Weidenhausen, Merrin Vincent, Kaarjel K. Narayanasamy, Anna Dimitriadi, Arif Ul Maula Khan, Martin Fritsch, Christoph W. Müller, Timo Zimmermann, Claire Deo*

European Molecular Biology Laboratory. Meyerhofstraße 1, 69117 Heidelberg, Germany.

◊These authors contributed equally

Email: claire.deo@embl.de

Table of Contents

Supplementary Figures 2
Supplementary Tables 28
General Experimental Information 34
UV-Vis and Fluorescence Spectroscopy 34
Cloning, Screening, Protein Expression and Purification 36
Structure Prediction 39
X-Ray crystallography 39
Cell Culture and Microscopy 40
References 43

Supplementary Figures

Figure S1. Engineering of psHaloTag0.1. **a**. Crystal structure of HaloTag bound to tetramethylrhodamine HaloTag ligand (PDB: 6Y7A).[1] The yellow spheres indicate positions selected for introduction of the sAsLOV2 domain. **b**. Schematic representation of the different types of constructs generated including insertion of sAsLOV2 into HaloTag, and N- or C-terminal fusion of sAsLOV2 to circularly permutated HaloTag. “XXX” in the name of the constructs denotes the amino acid position of the insertion or circular permutation. White boxes in the scheme depict the different linkers (G, glycine; cpL, circular permutation linker $(GGTGGS)_{x3}$) **c**. Performance of the HaloTag-sAsLOV2 constructs tested, showing the normalized fluorescence in the dark vs the bound fraction of **JF_{635}-HTL** after 2 hours of incubation. Values were normalized to $HaloTag_{635}$. While screening in lysate leads to high variability in measurements, constructs could be classified between “non-binding”, “slow binding” or “fast binding”, and among the fast-binding constructs, separated whether the **JF_{635}** fluorophore is mostly open or closed. The red-colored dots indicate the three constructs that showed a fluorescence change upon illumination at 450 nm (#1, #2, #3). **d.** Characterization of the generated HaloTag-sAsLOV2 proteins generated labelled with **JF_{635}-HTL**. The fluorescence intensity, binding capability, and deep-red absorption change upon illumination at 450 nm were measured. **e**. Absorption spectra of constructs #1, #2, #3 labelled with **JF_{635}-HTL** (100 nM), measured as purified protein in the dark and after illumination at 450 nm. n.m.: not measured.

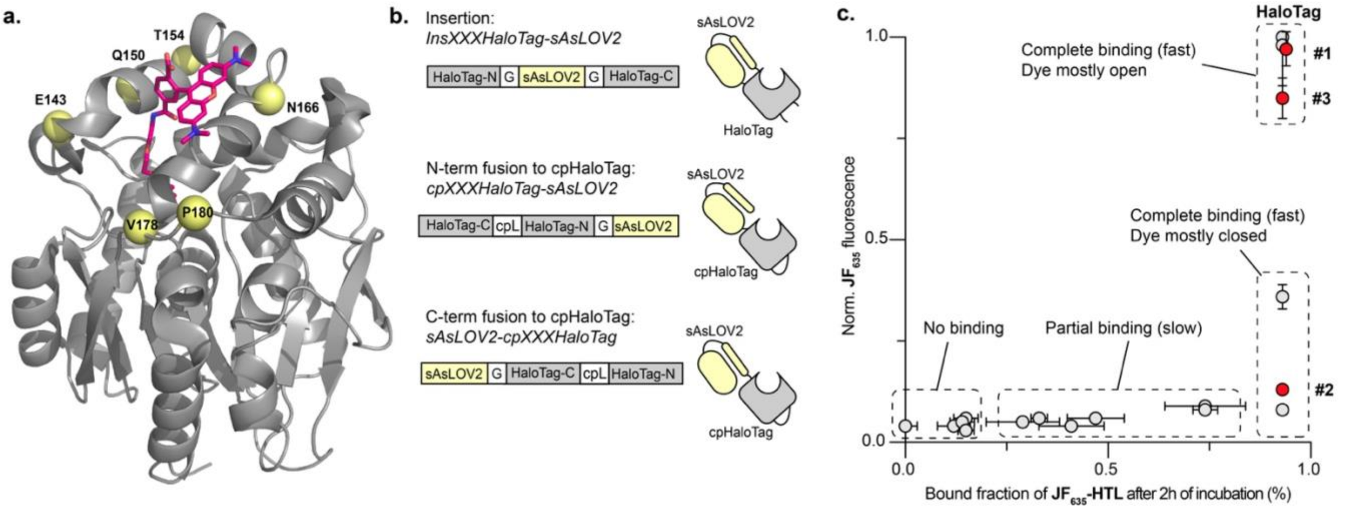

d.

Position	#	Construct	Norm. JF_{635} fluorescence	Bound fraction of JF_{635}-HTL (%)	A/A_0
NA		HaloTag	1	93 ± 1	n.m.
NA		sAsLOV2	0.05 ± 0.01	14 ± 3	n.m.
NA	#1	sAsLOV2-HaloTag	0.97 ± 0.04	94 ± 1	0.9
143	#2	ins143HaloTag-sAsLOV2	0.13 ± 0.01	93 ± 1	0.5
143		sAsLOV2-cp143HaloTag	0.36 ± 0.03	93 ± 1	0
143		cp143HaloTag-sAsLOV2	0.08 ± 0.01	93 ± 1	0
150		ins150HaloTag-sAsLOV2	0.04 ± 0.01	12 ± 4	n.m.
154	#3	ins154HaloTag-sAsLOV2	0.85 ± 0.05	93 ± 1	0.7
154		sAsLOV2-cp154HaloTag	0.98 ± 0.1	93 ± 1	0
166		ins166HaloTag-sAsLOV2	0.03 ± 0.01	15 ± 2	n.m.
166		sAsLOV2-cp166HaloTag	0.04 ± 0.01	0 ± 3	n.m.
178		ins178HaloTag-sAsLOV2	0.06 ± 0.01	15 ± 3	n.m.
178		sAsLOV2-cp178HaloTag	0.06 ± 0.01	33 ± 2	n.m.
178		cp178HaloTag-sAsLOV2	0.04 ± 0.01	41 ± 8	0
180		ins180HaloTag-sAsLOV2	0.06 ± 0.01	47 ± 7	0
180		sAsLOV2-cp180HaloTag	0.05 ± 0.01	29 ± 9	n.m.
180		ins180Halo-SG-sAsLOV2	0.09 ± 0.01	74 ± 10	0
180		ins180Halo-sAsLOV2-SG	0.08 ± 0.01	74 ± 3	0

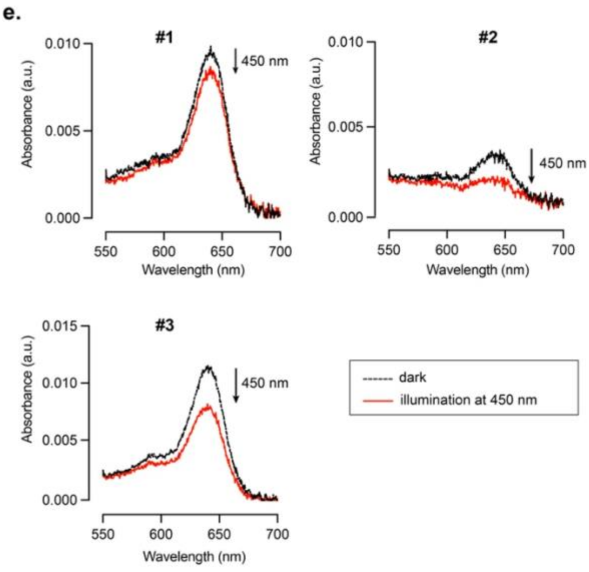

Figure S2. Schematic of the *in vitro* screening assay used for the engineering of psHaloTag. Following generation of a plasmid library through site-saturation mutagenesis and transformation in *E. coli*, individual colonies were manually picked in a 96 well-plate. After expression and lysis as described in the methods, **JF_{635}-HTL** (50 nM) was added to each well and incubated for 2 hours at room temperature in the dark. The labelled lysate was divided into two. Half was used to measure the binding efficiency, by addition of excess purified HaloTag protein to capture all unbound **JF_{635}-HTL**, and calculate the bound-fraction using fluorescence measurements. The second half of the lysate was used to perform the photoswitching assay, by three cycles of illumination at 450 nm (3 minutes for each cycle). Fluorescence measurements enabled to calculate F/F_0 of each construct, and additionally assess the photoswitching kinetics. r.t.: room temperature.

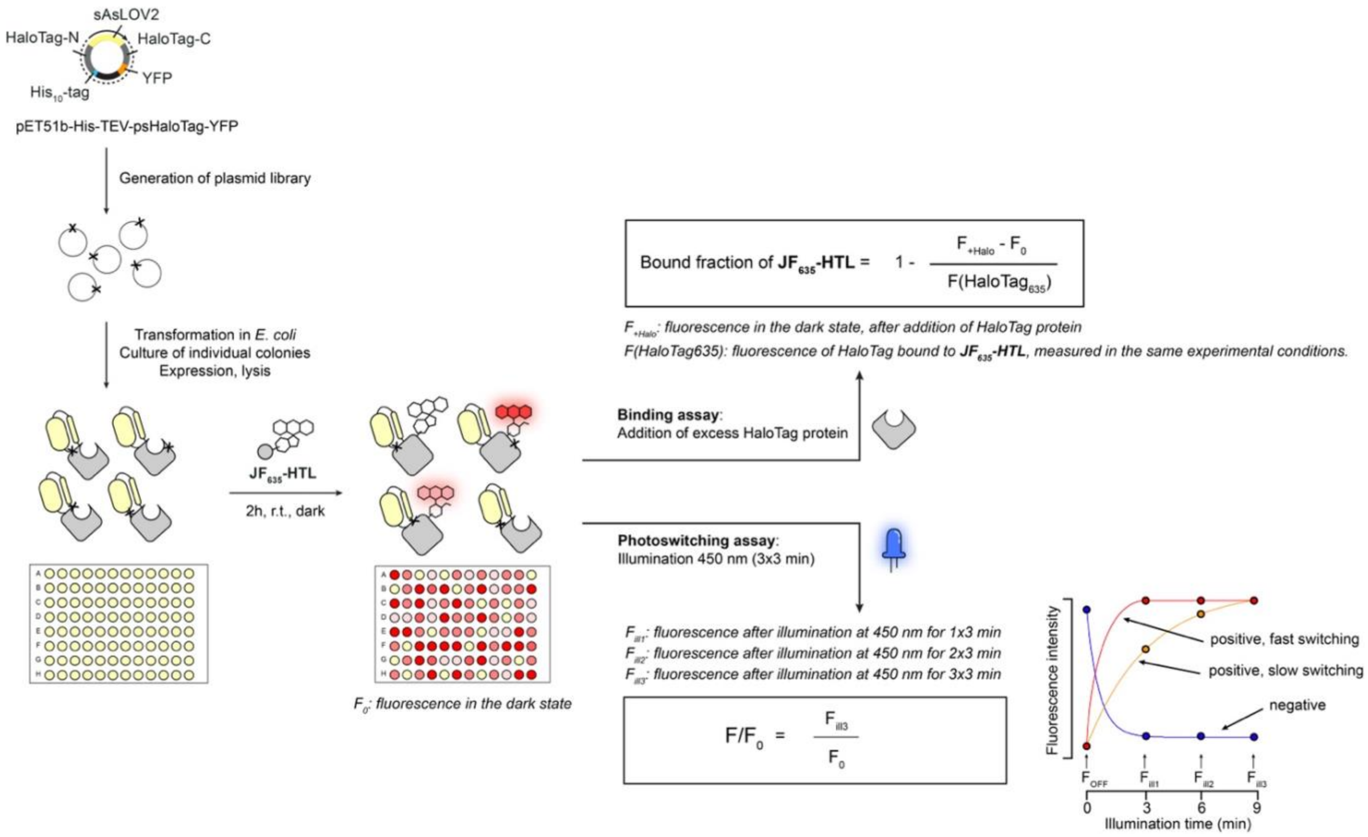

Figure S3. Picture of the custom-built illumination device used for inducing photoswitching of psHaloTag *in vitro* and *in cellulo*. The illumination box was made to fit a 96-well plate, and comprises 24 LEDs at 450 nm, and a controller to trigger illumination.

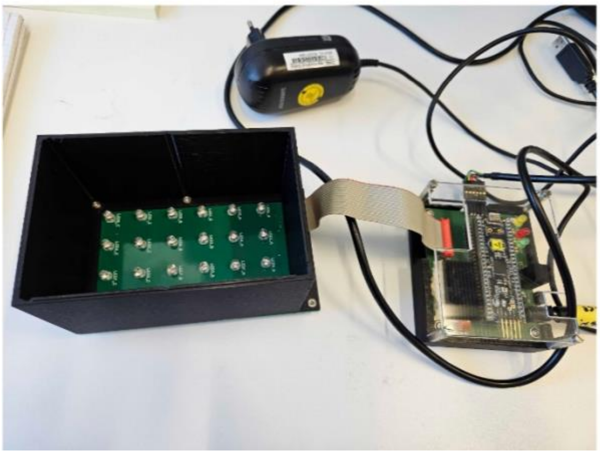

Figure S4. Stepwise engineering of psHaloTag1a and 1b, showing detail of the positions saturated in each round and schematic sequence representation of isolated variants. The first round of mutagenesis was performed in parallel on 3 constructs: psHaloTag0.1, psHaloTag0.1 with insertion of PE in the linker at the C-side of sAsLOV2, and psHaloTag0.1 with insertion of FA in the linker at the N-side of sAsLOV2. Full sequences of the isolated constructs in each round can be found in Table S1.

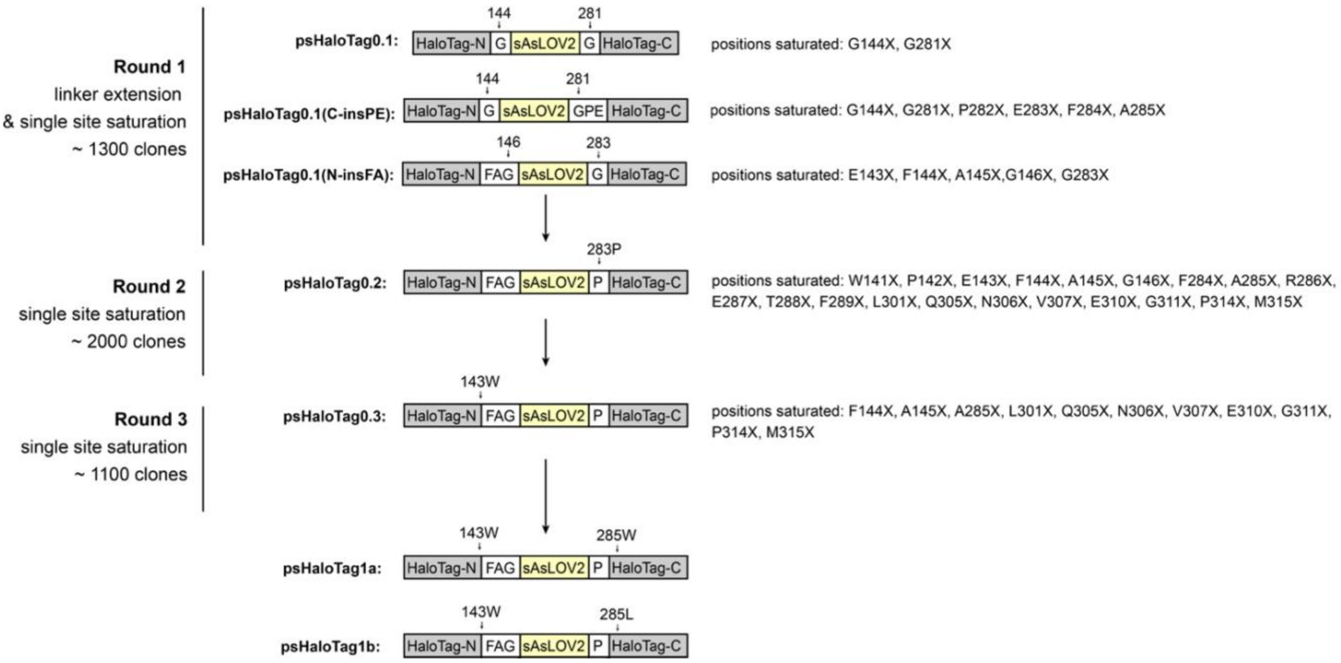

Figure S5. Absorption (left panel) and emission (center left panel) spectra of selected psHaloTag variants labelled with **JF_{635}-HTL**, in the dark (dashed black) and after illumination at 450 nm (red). Kinetics of the photoswitching (turn-on under 450 nm illumination, center right panel) and thermal relaxation (turn-off in the dark, right panel) of the FMN cofactor (yellow) and **JF_{635}** (red) of selected variants. Absorption measurements were performed at 5 μM dye ligand and 7.5 μM protein, and fluorescence measurements at 1 μM dye ligand and 1.5 μM protein, in triplicate. Kinetic profiles are plotted as mean and SEM, and fitted to a single exponential curve. n.m.: not measured.

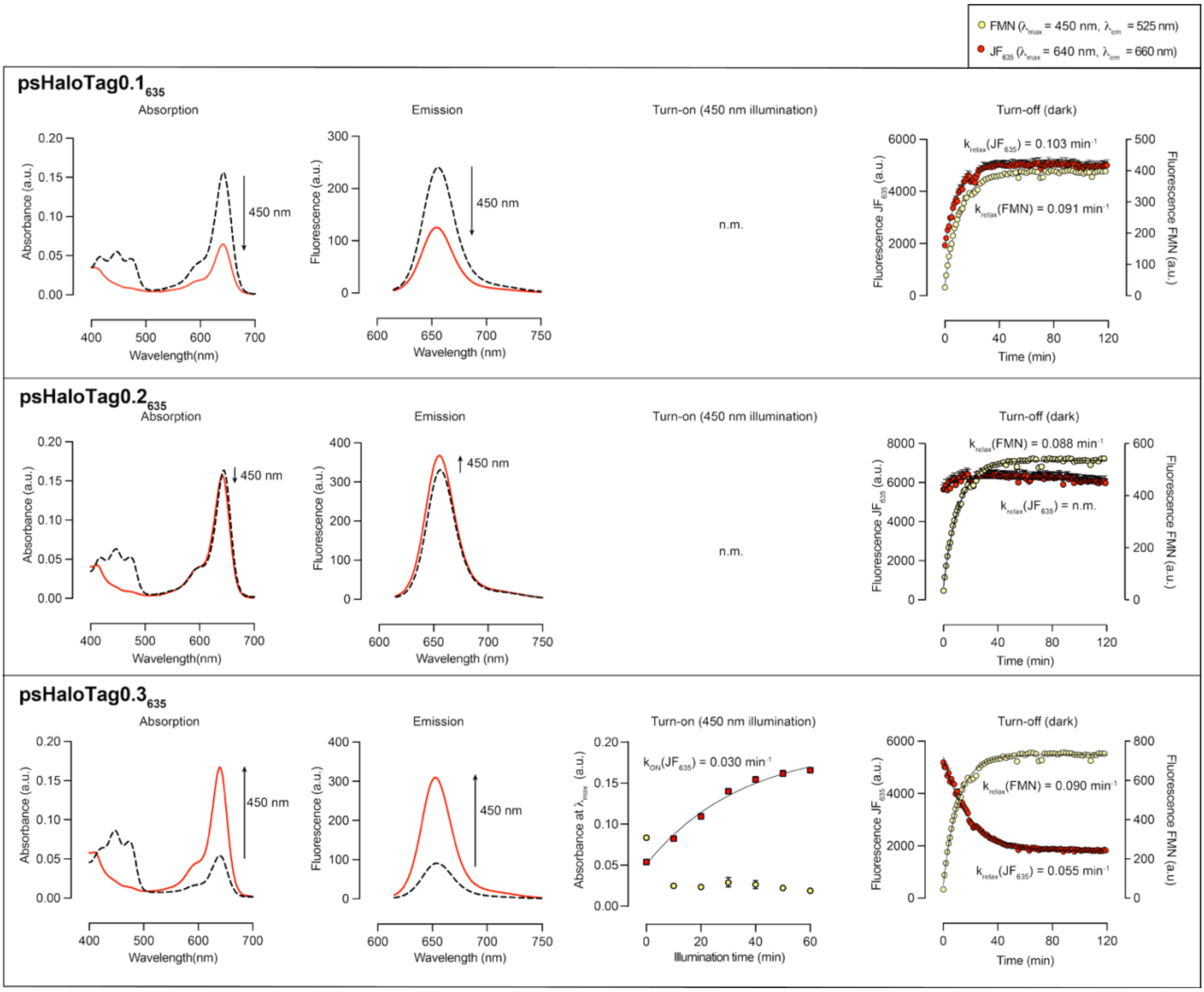

Figure S5 – Continued.

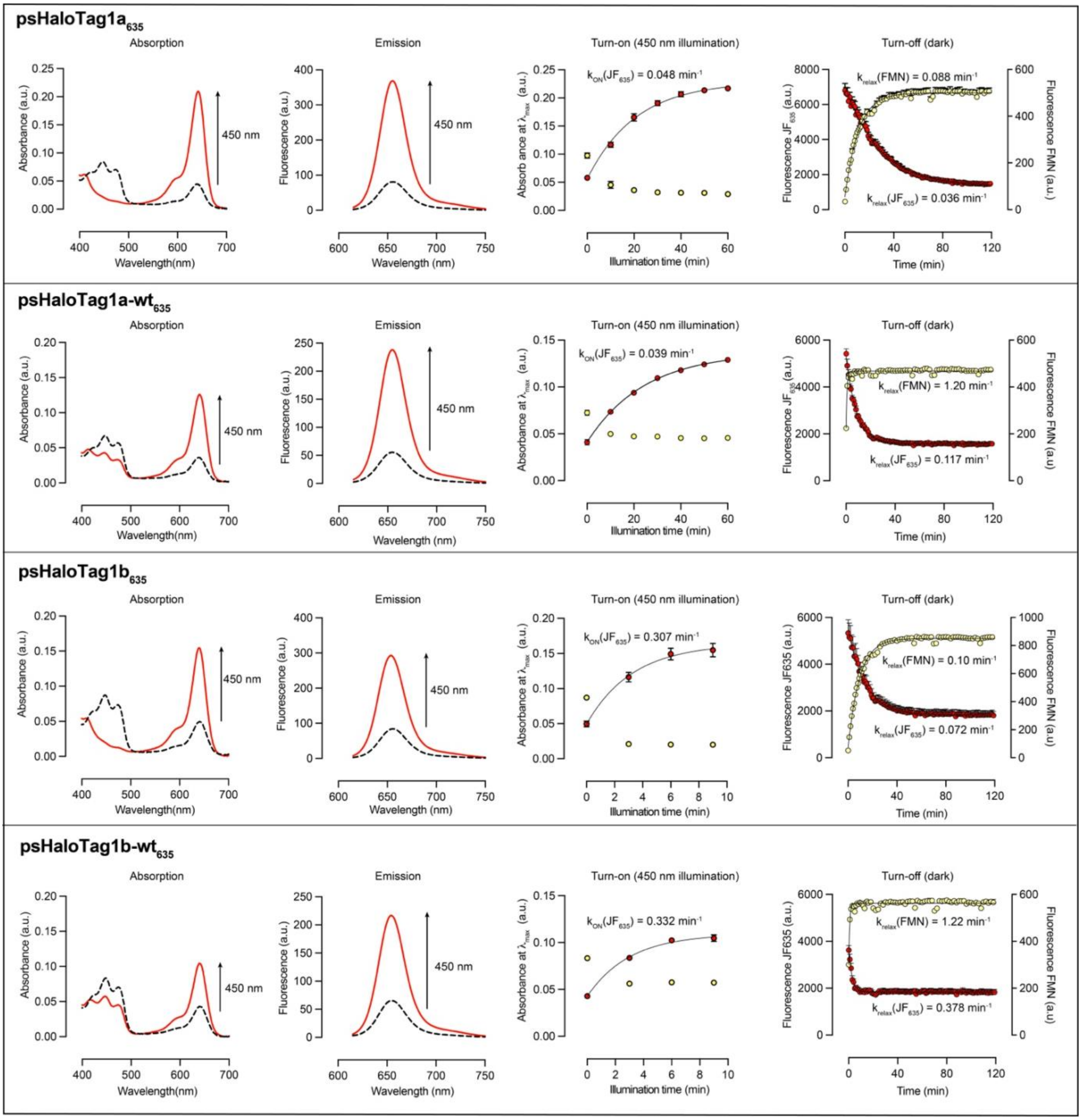

Figure S6. Labelling of psHaloTag1a (7.5 μM) with **JF_{635}-HTL** (5 μM) measured by the increase in absorbance at 640 nm when incubated in the dark. Labelling was complete after 15 minutes. Mean and SEM for 3 replicates.

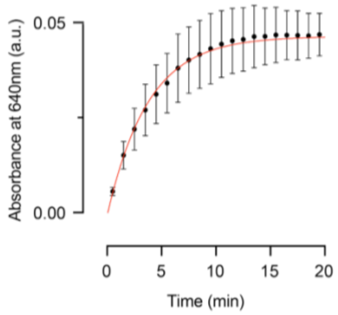

Figure S7. pH-dependence of the fluorescence and photoswitching properties of psHaloTag1a$_{635}$. **a**. Fluorescence intensity ($\lambda_{ex}/\lambda_{em}$ = 640/660 nm) of psHaloTag1a$_{635}$ at different pHs in the dark and after illumination at 450 nm. **b**. F/F_0 as a function of pH for psHaloTag1a$_{635}$. **c**. Apparent relaxation rate constant at room temperature in the dark for the FMN signal and the **JF_{635}** signal for psHaloTag1a$_{635}$. Plots represent mean and SEM for 3 replicates.

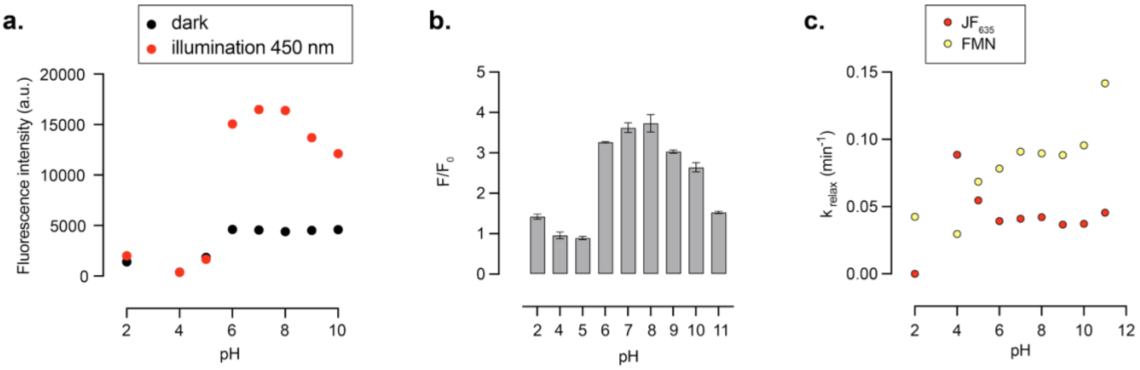

Figure S8. Absorption and fluorescence spectra of psHaloTag1a635 (**a**.), psHaloTag1b635 (**b**.) and psHaloTag1a630 (**c**.) in the dark (solid black line) and following illumination at 450 nm (solid red line). Absorption and emission spectra of the “dark” mimic (mutant C189A removing the reactive cysteine and abolishing the photocycle, dashed black line) and “lit” mimic (double mutant I271E, A275E, dashed red line) for the same constructs labelled with the same fluorophore ligands.

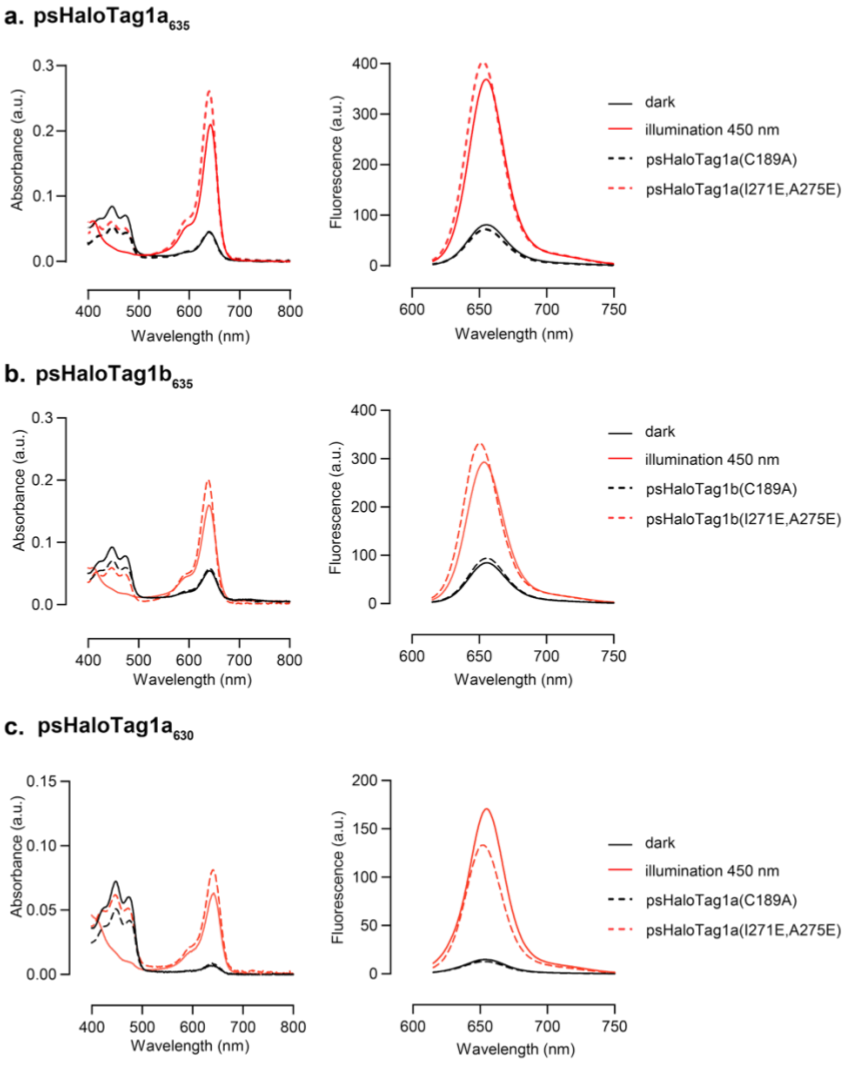

Figure S9. Fluorescence of a solution of purified psHaloTag1a$_{635}$ (1 µM **JF$_{635}$-HTL**, 2.5 µM psHaloTag1a) over time upon illumination at 450 nm at different light intensity. Above 0.1 mW·cm^{-2}, the photoconversion is intensity-independent (the light-independent conformational change is the rate-limiting step), whereas at 0.01 mW·cm^{-2}, the process is light-limited and the sample cannot reach maximal fluorescence state.

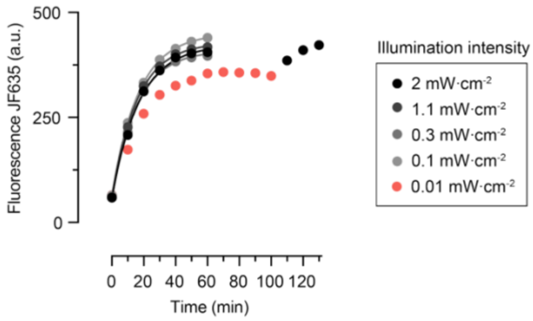

Figure S10. a. General structure and open close equilibrium of fluorogenic HaloTag ligands. **b**. Chemical structure of fluorogenic HaloTag ligands tested, and properties of psHaloTag1a when labelled with those dye ligands. n.m.: not measured.

a.

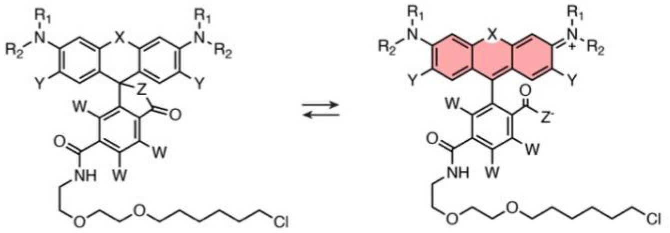

b.

	X	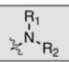	Y	Z	W	$\lambda_{max}/\lambda_{em}$ (nm)	A/A$_0$	F/F$_0$	$t_{1/2, ON}$(JF$_{635}$) (min)	$t_{1/2, relax}$(FMN) (min)	$t_{1/2, relax}$(JF$_{635}$) (min)
JF$_{525}$-HTL	O	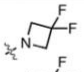	H	O	H	525/549	n.m	1.2	n.m.	n.m.	n.m.
JF$_{526}$-HTL	O	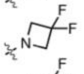	F	O	H	526/550	n.m.	1.2	n.m.	n.m.	n.m.
JF$_{585}$-HTL	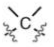	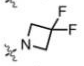	H	O	H	585/609	n.m.	2.0	n.m.	n.m.	n.m.
MaP$_{618}$-HTL	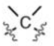	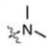	H	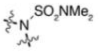	H	618/635	n.m.	1.2	n.m.	n.m.	n.m.
JF$_{626}$-HTL (2)	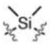	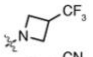	H	O	H	626/638	2.1	3.4	5.5	9.8	23.1
JF$_{629}$-HTL (3)	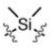	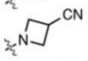	H	O	H	629/648	4.0	6.7	11.3	10.5	16.1
JF$_{630}$-HTL (4)	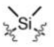	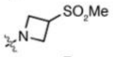	H	O	H	630/649	9.2	9.4	28.9	13.3	26.6
JF$_{635}$-HTL (1)	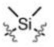	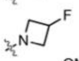	H	O	H	635/652	4.7	4.6	14.3	7.9	19.5
JF$_{639}$-HTL (5)	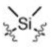	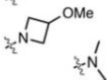	H	O	H	639/656	n.m.	2.5	n.m.	n.m.	n.m.
MaP$_{700}$-HTL	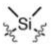	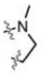		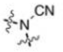	H	700/720	n.m.	1.6	n.m.	n.m.	n.m.
JF$_{711}$-HTL	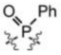	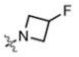	H	O	F	711/732	n.m.	1.2	n.m.	n.m.	n.m.

Note: **JF$_{635}$-HTL** was synthesized as previously described (see characterization in Figure S22).[2] The HaloTag ligands of **JF$_{525}$**,[2] **JF$_{526}$**,[3] **JF$_{585}$**,[2] **JF$_{626}$**,[4] **JF$_{629}$**,[4] **JF$_{630}$**,[4] **JF$_{639}$**,[4] **JF$_{711}$**[5] were gifted by the Lavis group (Janelia Research Campus, HHMI). The HaloTag ligands of **MaP$_{618}$**[6] and **MaP$_{700}$**[6] were gifted by the Johnsson group (Max Planck Institute for Medical Research).

Figure S11. Absorption (left panel) and emission (center left panel) spectra psHaloTag1a labelled with **JF_{626}-HTL**, **JF_{629}-HTL** or **JF_{630}-HTL** in the dark (dashed black) and after illumination at 450 nm (red). Absorption kinetics measured at λ_{max} of the photoswitching (turn-on under 450 nm illumination, center right panel) and thermal relaxation (turn-off in the dark, right panel) for the signal of the FMN cofactor (yellow) and dye (red) of selected variants. Absorption measurements were performed at 5 µM dye ligand and 7.5 µM protein, and fluorescence measurements at 1 µM dye ligand and 1.5 µM protein, in triplicate. Kinetics curves are plotted as mean and SEM, and fitted to a single exponential curve. λ_{max} = 637 nm for **JF_{626}**; 638 nm for **JF_{629};** 640 nm for **JF_{630}**; 450 nm for FMN.

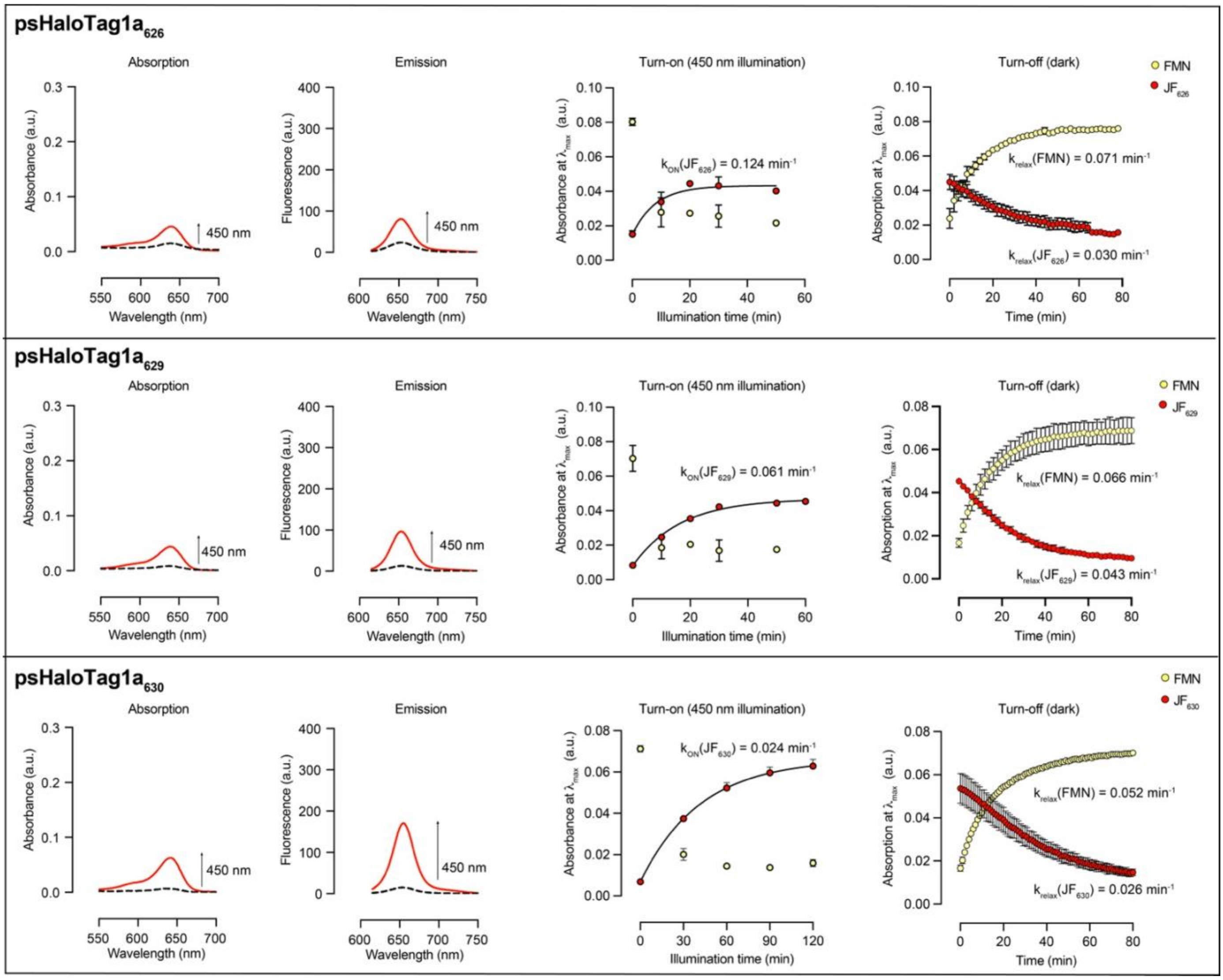

Figure S12. AlphaFold predicted structures (**a,c.**) and corresponding pLDDT maps (**b,d.**) of psHaloTag1a (**a,b.**) and psHaloTag1b (**c,d.**). The HaloTag domain is in grey and sAsLOV2 domain in yellow in **a**,**c**. The amino acids corresponding to mutations introduced during rounds of mutagenesis are depicted as sticks.

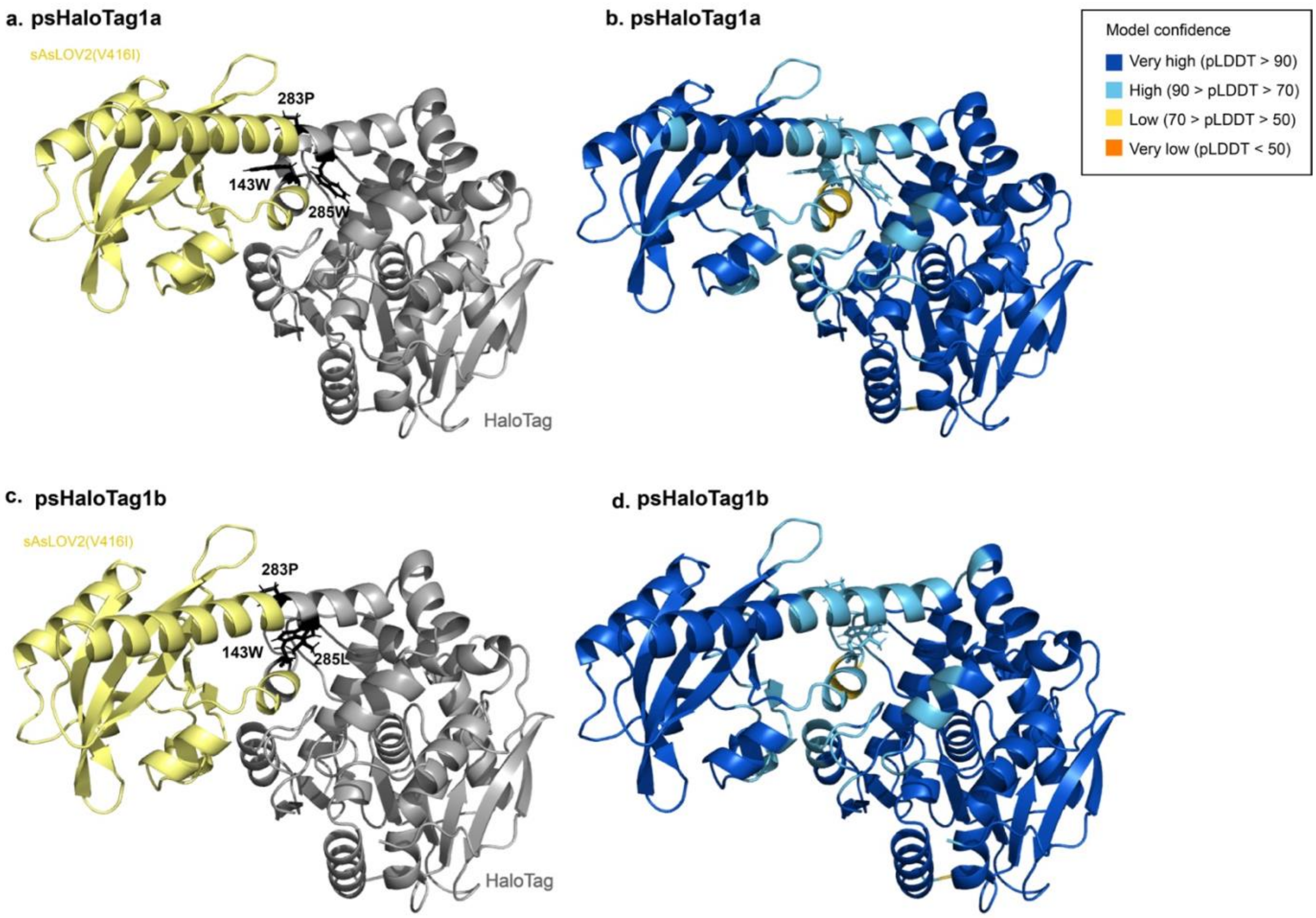

Figure S13. a. The crystal structure of psHaloTag1a with **JF$_{635}$-HTL** and FMN contains two chains with one molecule of each psHaloTag1a, **JF$_{635}$-HTL** (magenta), FMN (orange), and Cl^- (cyan) in its asymmetric unit. The two rhodamines (magenta) from chain A (green) and chain B (salmon) are facing each other in the center. **b.** Superimposition of the two assemblies reveals a near identical structure for both protein chains and positioning of the ligands with a root-mean-square deviation (RMSD) of 0.33 Å (for 431 Cα residues). **c.** Comparison of an AlphaFold model (blue) with chain A (green) shows similar folds for the HaloTag and LOV domain part with an RMSD of 1.64 Å (for 371 Cα residues). Here, the HaloTag domain was used for superimposition. A slightly different conformation in the prediction of the Jα helix and the linker region before the Jα helix leads to an offset between the predicted and experimentally determined position of the LOV domains. **d.** Fitting of both FMN molecules into the electron density maps (grey mesh) and the potential covalent binding partner Cys189. Based on the electron density, both FMN molecules do not seem covalently bound. **e.** Fitting of both **JF$_{635}$-HTL** dyes with the released Cl^- and the covalently linked Asp106 residues into the electron density maps (grey mesh). Based on the electron density, the open conformation of the dye fitted best in both chains. The $2mF_{obs}$-DF_{calc} map is at a contour level of 1 σ.

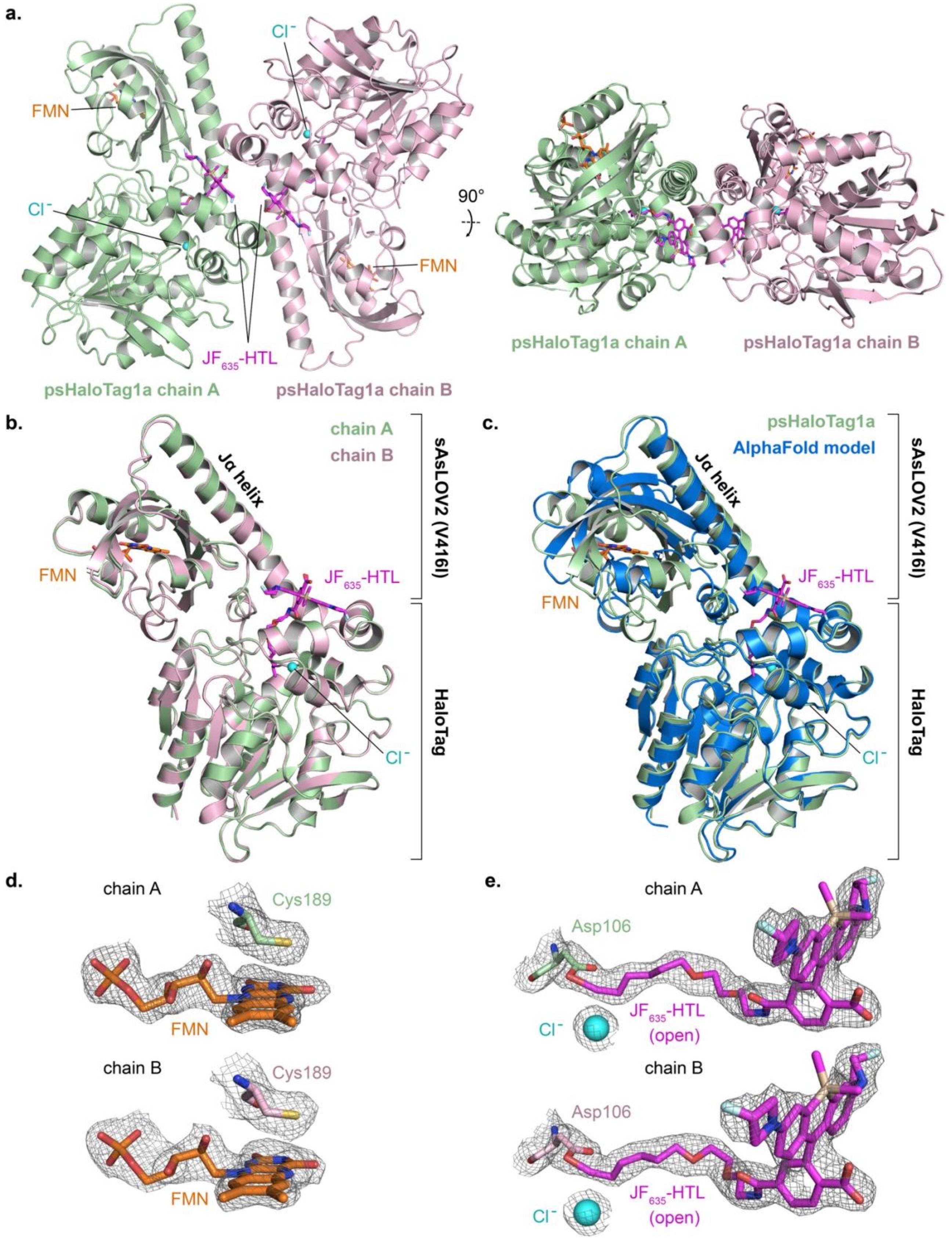
a.
Cl⁻
FMN
Cl⁻
FMN
JF635-HTL
psHaloTag1a chain A
psHaloTag1a chain B
90°
psHaloTag1a chain A
psHaloTag1a chain B
b.
chain A
chain B
Jα helix
FMN
JF635-HTL
Cl⁻
sAsLOV2 (V416I)
HaloTag
c.
psHaloTag1a
AlphaFold model
Jα helix
JF635-HTL
FMN
Cl⁻
sAsLOV2 (V416I)
HaloTag
d.
chain A
Cys189
FMN
chain B
Cys189
FMN
e.
chain A
Asp106
JF635-HTL
(open)
Cl⁻
chain B
Asp106
JF635-HTL
(open)
Cl⁻

Figure S14. a. The crystal structure of psHaloTag1a with **JF_{635}-HTL** and FMN compares well with available crystal structures of HaloTag7 bound to **JF_{669}-HTL** (sand, PDB: 8SW8)[7] and the dark state of AsLOV2 domain (brown, PDB: 2V1A).[8] Superimposition resulted in root-mean-square deviations (RMSD) of 0.50 Å (for 293 Cα residues) for the HaloTag moiety and 1.04 Å (for 137 Cα residues) for the LOV domain, respectively. **b.** Side-view of the superimposed psHaloTag1a structure (green) with HaloTag7 (sand) bound to **JF_{669}-HTL**. The position of the rhodamine moieties of **JF_{635}-HTL** (magenta) and **JF_{669}-HTL** (grey) is not identical, highlighting flexibility. However, as the dyes are surface-exposed, different crystal contacts could affect the different positions. **c.** Electrostatic surface potential representation of psHaloTag1a with the same view as in (b.) displays a negative surface potential around the surface-exposed rhodamine moiety of **JF_{635}-HTL**. Electrostatics are displayed in a gradient from negative (red, -5 kT/e) to positive (blue, +5 kT/e) surface potential. **d.** Electrostatic surface potential representation of HaloTag7 with bound **JF_{669}-HTL** with the same view as in (b.) displays a negative surface potential around the surface-exposed rhodamine moiety of **JF_{669}-HTL**. There is more surface exposure compared to psHaloTag1a (c.) because of the non-present LOV domain. Electrostatics are displayed in a gradient from negative (red, -5 kT/e) to positive (blue, +5 kT/e) surface potential.

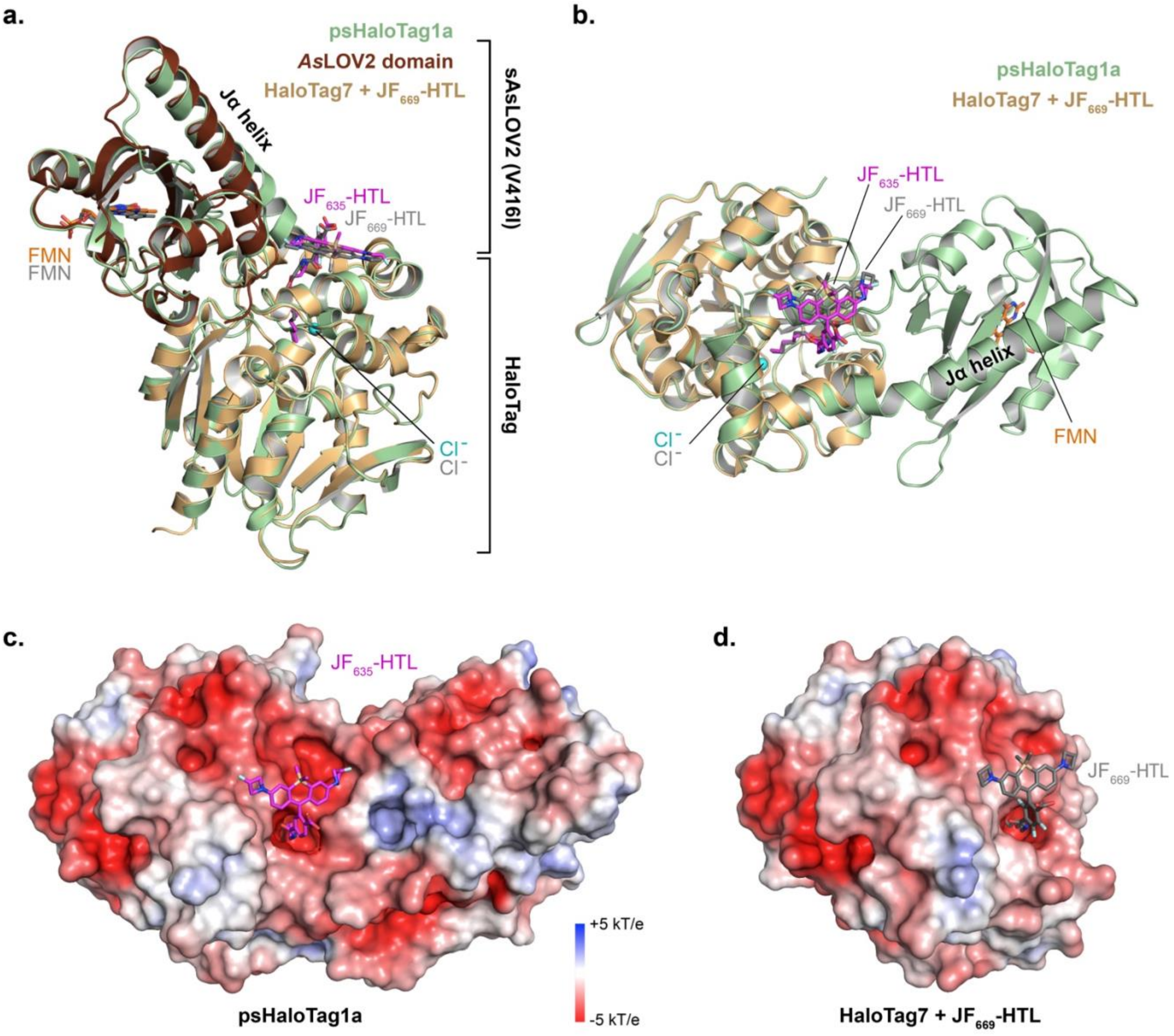

Figure S15. Cellular localization of H2B-psHaloTag1a$_{635}$ and TOMM20-psHaloTag1a$_{635}$ in U2OS cells. **a-b**. Representative images of U2OS cells expressing H2B-psHaloTag1a-T2A-EGFP (**a**.) and TOMM20-psHaloTag1a-T2A-EGFP (**b**.), labelled with **JF$_{635}$-HTL** and a specific intracellular marker (Hoeschst 33342 for nucleus in **a**.; LumiTracker Mito Orange CMTMRos for mitochondria in **b**.) and Pearson correlation coefficients (right panels) using ImageJ/Fiji software and coloc2 plugin (N = 50 transfected cells for single labelling to discard possible bleed-through; N = 100 transfected cells with both labels). Scale bars: 50 µm. Image color code: grey (EGFP), green (intracellular marker), **JF$_{635}$-HTL** (magenta). **c-d**. Representative images of a transfected cell expressing H2B-psHaloTag1a-T2A-EGFP (**c**.) or TOMM20-psHaloTag1a-T2A-EGFP (**d**.), labelled with **JF$_{635}$-HTL** (magenta) and the corresponding specific cellular marker (green: Hoeschst 33342 for nucleus in **c**.; LumiTracker Mito Orange CMTMRos for mitochondria in **d**.). For co-localization analysis, the EGFP channel (grey) was selected for to draw a ROI corresponding to a single cell (yellow line), to calculate the Pearson correlation coefficient (PCC) using ImageJ/Fiji software and coloc2 plugin.

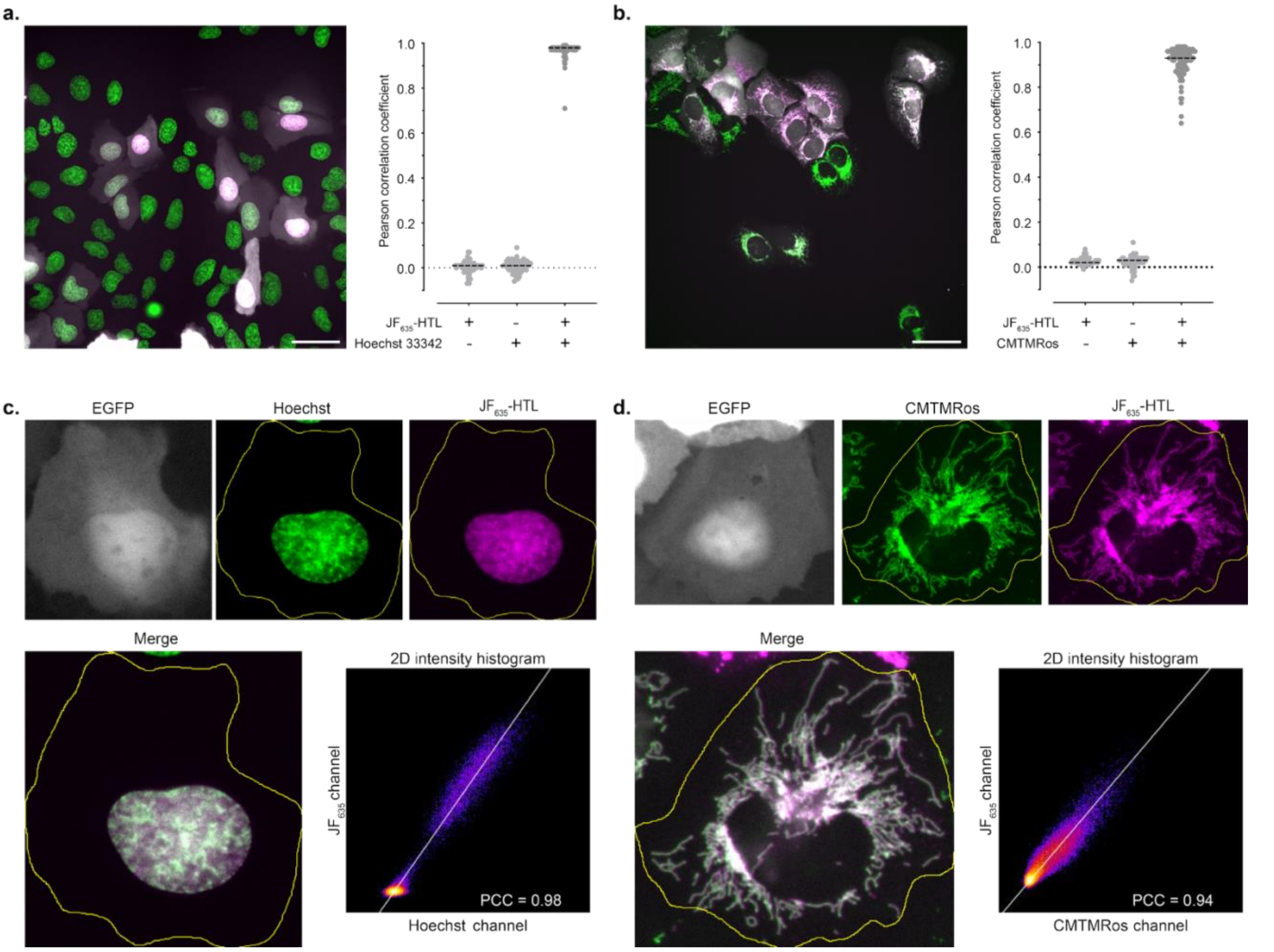

Figure S16. Cell viability of nontransfected or transfected U2OS cells expressing H2B-psHaloTag1a, LifeAct-psHaloTag1a or TOMM20-psHaloTag1a after incubation with 1 µM **JF_{635}-HTL,** in the dark or under continuous illumination (450 nm, 2 mW·cm^{-2}, 1 h). The data shown correspond to 3 independent experiments performed in triplicates where unpaired *t* test showed no significant p values ($p > 0.005$ in all cases).

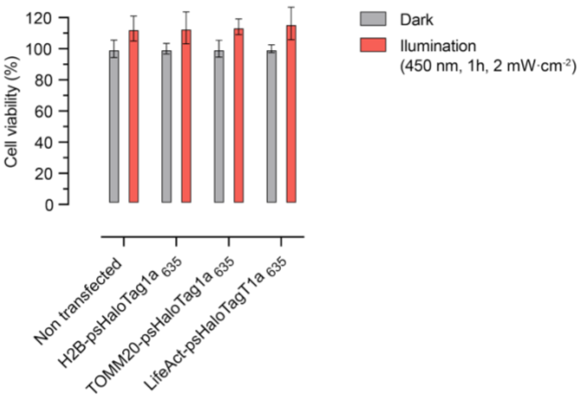

Figure S17. a. Representative images of U2OS cells expressing LifeAct-psHaloTag1a-T2A-EGFP and labelled with **JF_{630}-HTL**, in the dark (left) and after illumination at 450 nm for 5 minutes (right). Scale bars: 50 µm. **b**. Intensity line profiles corresponding to the images in **a**. **c**. Normalized fluorescence intensity of cells expressing LifeAct-psHaloTag1a-T2A-EGFP and labelled with either **JF_{635}-HTL** or **JF_{630}-HTL**, upon cycles of illumination at 450 nm followed incubation in the dark. Quantification in panel **c.** was performed on N > 100 cells for each condition.

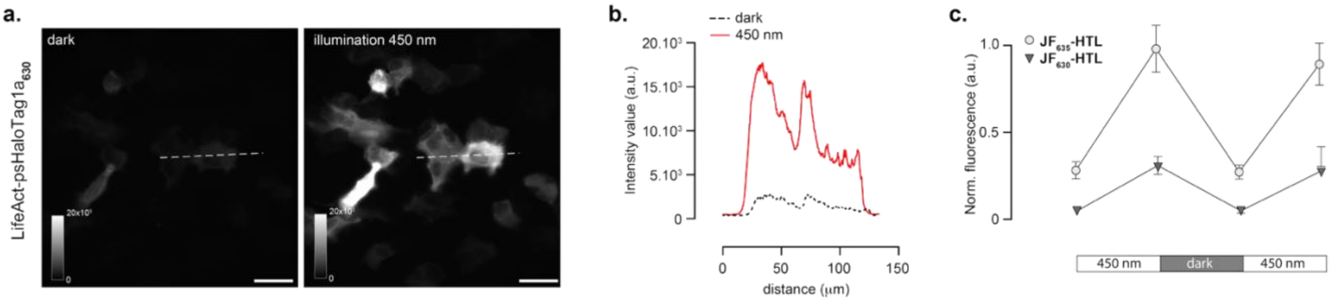

Figure S18. Additional example of sequential photoactivation of single cells. **a,c**. Representative confocal images of living U2OS cells expressing H2B-psHaloTag1a (**a**.) or H2B-HaloTag1a (**c**.) and labelled with **JF_{635}-HTL** in the dark (T_0) and following photoactivation of a single cell in the field of view (T_1-T_3). In each frame, the activated cell is indicated by a white arrow. Scale bars: 50 µm. **b,d**. Corresponding fluorescence intensity of individual cells for the frames in **a**. and **c**., respectively. Each curve corresponds to one cell with corresponding numbering in frame T_0.

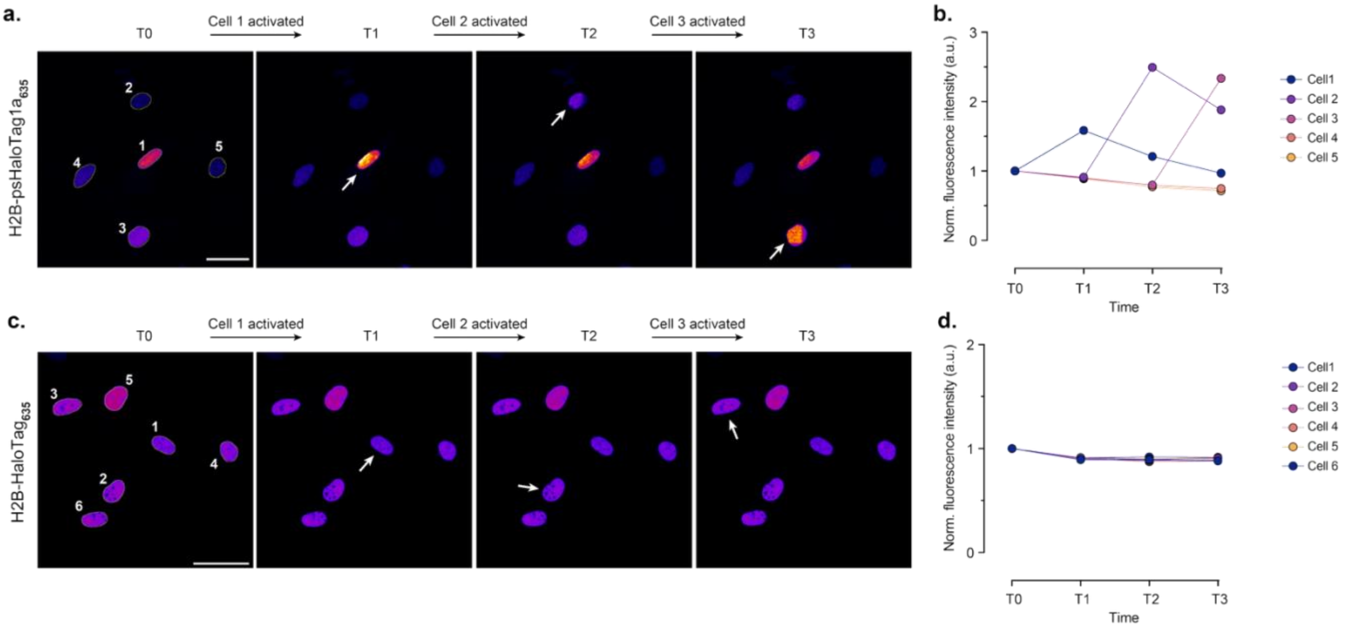

Figure S19. Additional representative example of SMLM imaging with psHaloTag1a$_{630}$b. **a**. Representative example of SMLM images of live U2OS cells expressing H2B-psHaloTag1a and labelled with **JF$_{630}$b-HTL** (25 nM) before illumination, during illumination at 450 nm, after thermal recovery and during a second illumination at 450 nm. Each reconstructed image was rendered from 5000 frames, acquired at 100 ms per frame. **b**. Number of localizations per 100 frames for the cell in panel a. **c**. Photon count per localization for each of the sequential acquisition frames in b.

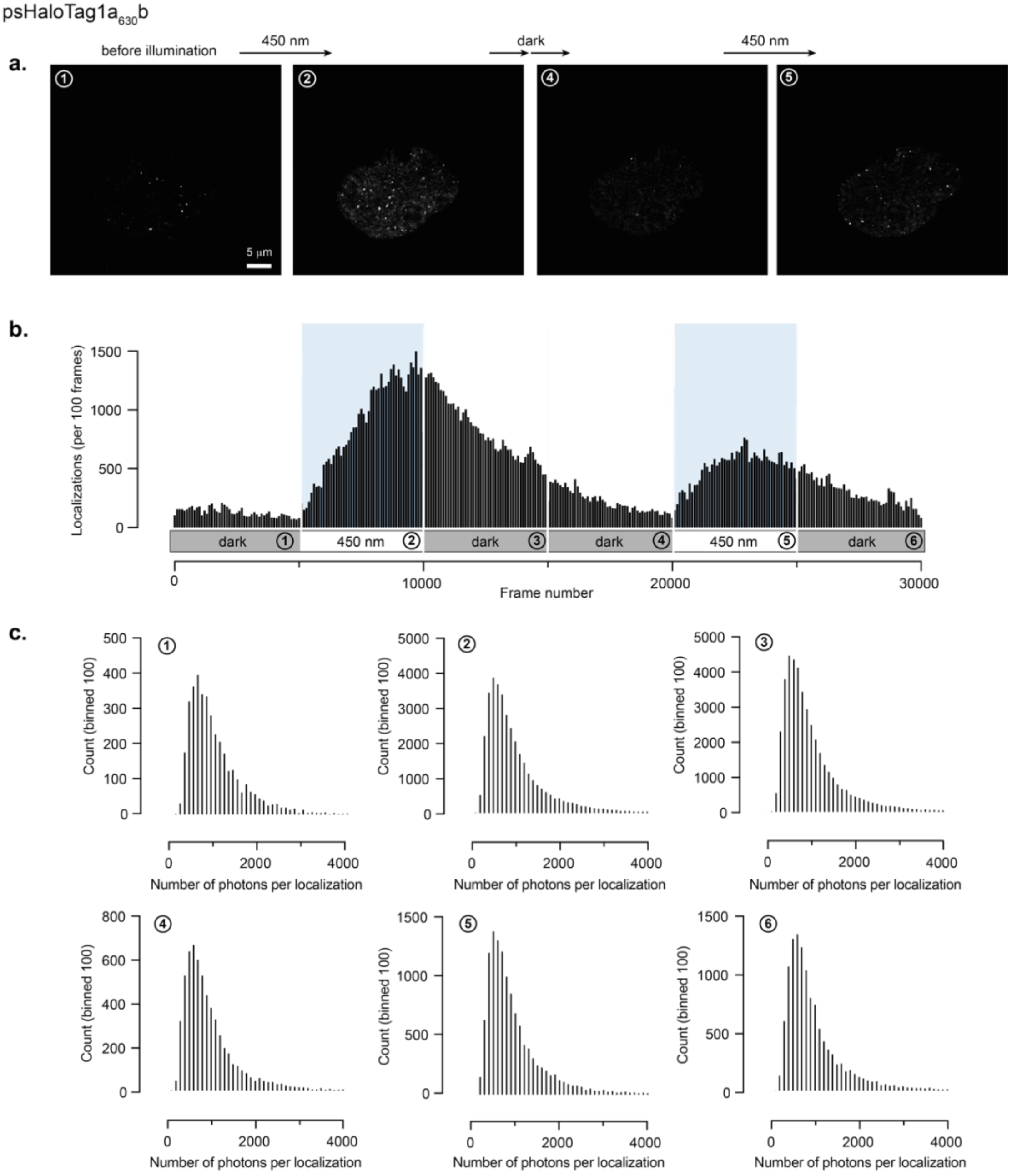

Figure S20. Representative example of SMLM imaging with psHaloTag1a$_{635}$b. **a**. Representative example of SMLM images of live U2OS cells expressing H2B-psHaloTag1a and labelled with **JF$_{635}$b-HTL** (25 nM) before illumination, during illumination at 450 nm, after thermal recovery and during a second illumination at 450 nm. Each reconstructed image was rendered from 5000 frames, acquired at 100 ms per frame. **b**. Number of localizations per 100 frames for the cell in panel a. **c**. Photon count per localization for each of the sequential acquisition frames in b.

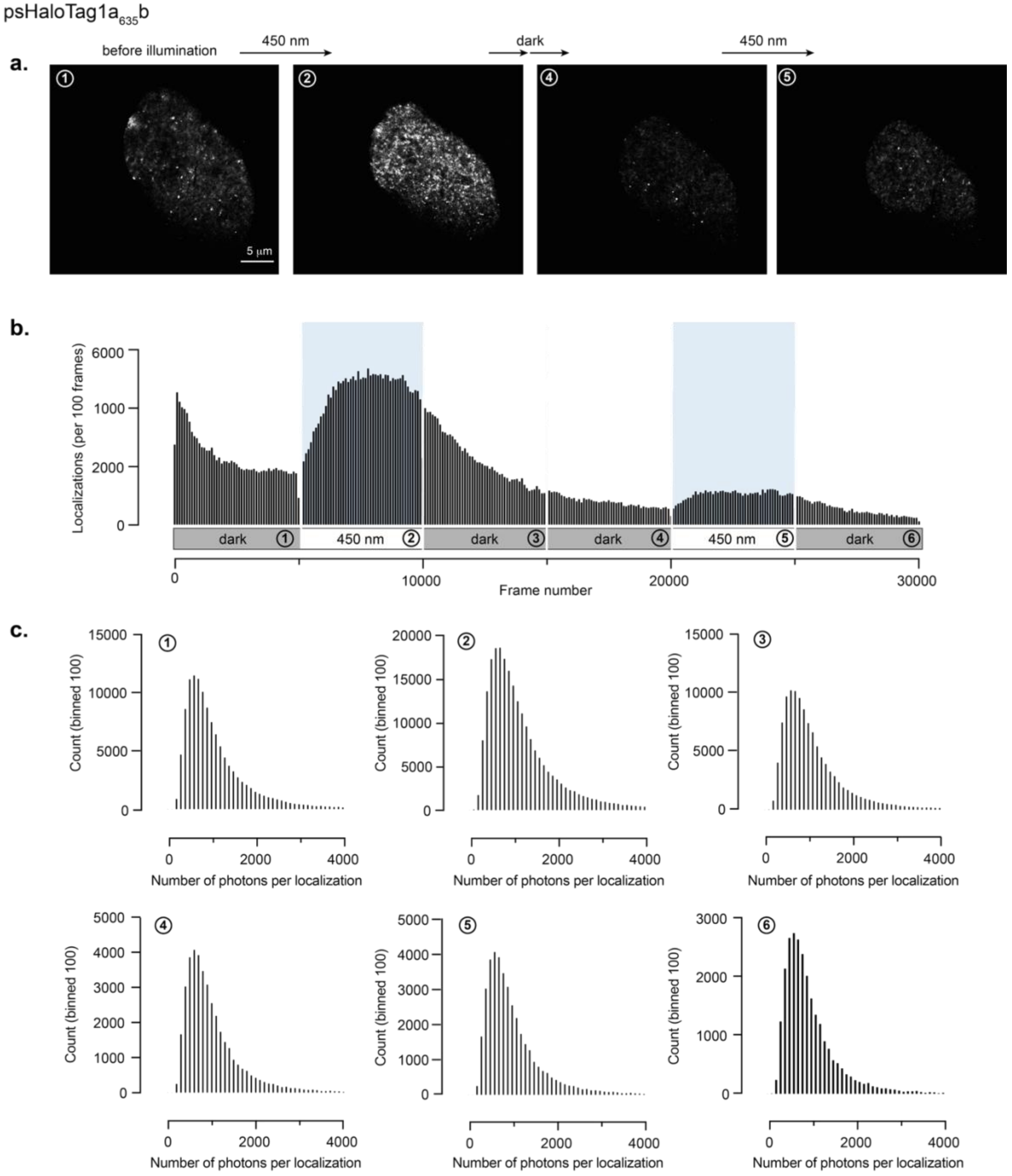

Figure S21. Representative example of SMLM imaging with HaloTag$_{630}$b. **a**. Representative example of SMLM images of live U2OS cells expressing H2B-HaloTag and labelled with **JF$_{630}$b-HTL** (25 nM) before illumination, during illumination at 450 nm, and after thermal recovery. Each reconstructed image was rendered from 5000 frames, acquired at 100 ms per frame. **b**. Number of localizations per 100 frames for the cell in panel a. **c**. Photon count per localization for each of the sequential acquisition frames in b.

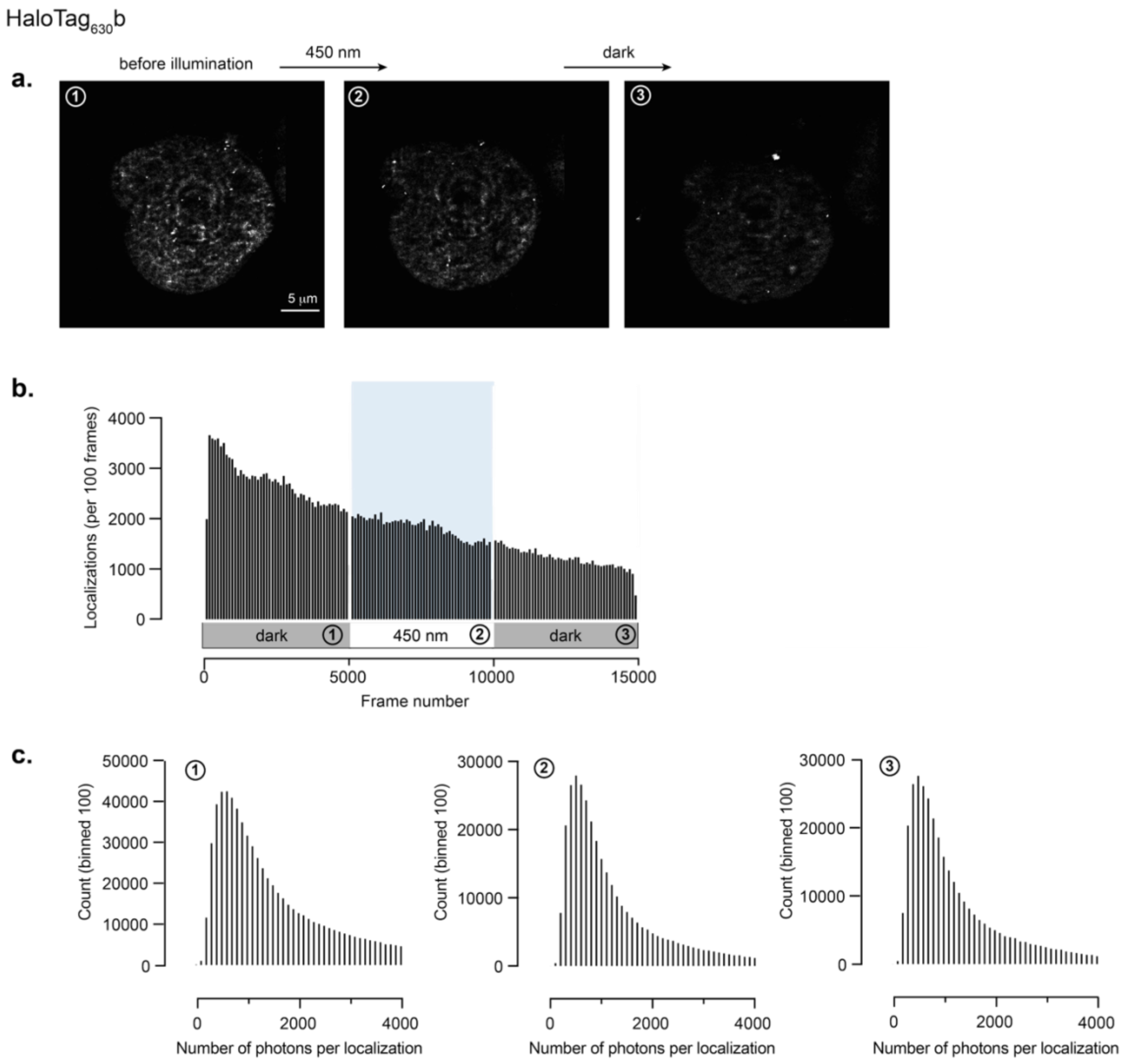

Figure S22. a. ^{1}H NMR (400 MHz, $CDCl_3$) spectrum and **b.** HPLC trace for **JF_{635}-HTL**. The compound was synthesized following the published procedure, and the characterization data is as reported.[2]

a. ^{1}H NMR (400 MHz, $CDCl_3$)

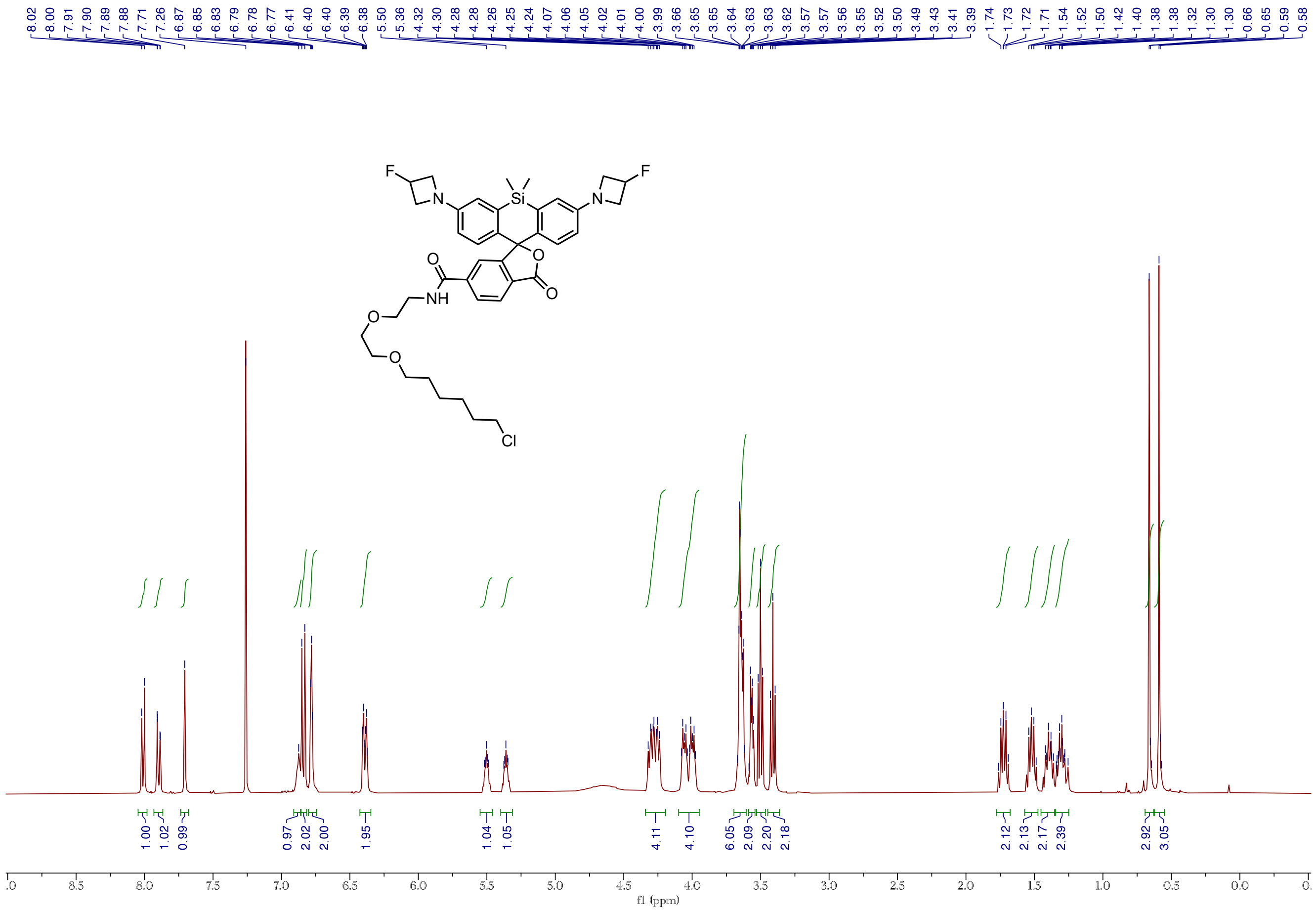

b. Analytical HPLC: 5–95% CH_3CN/H_2O gradient with constant 0.1% formic acid additive, 8 min run, 0.7 mL/min flow, UV detection at 254 nm. Purity >98%

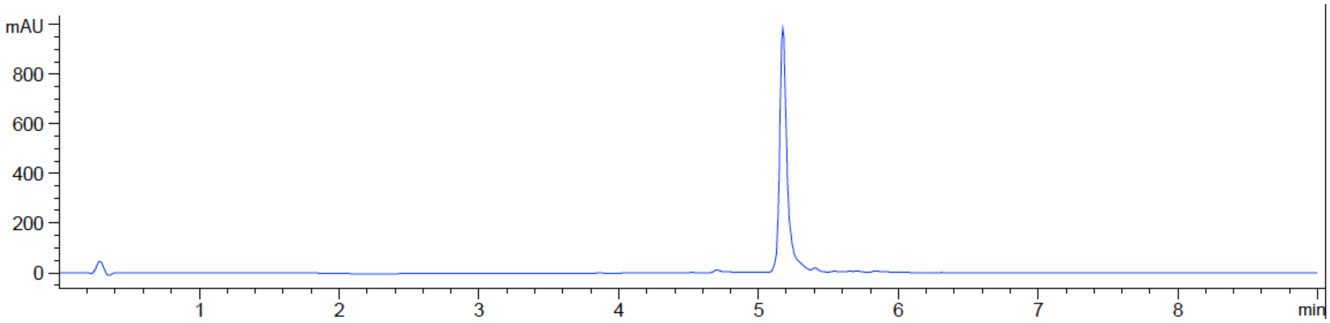

c. Mass spectrometry data corresponding to panel b.

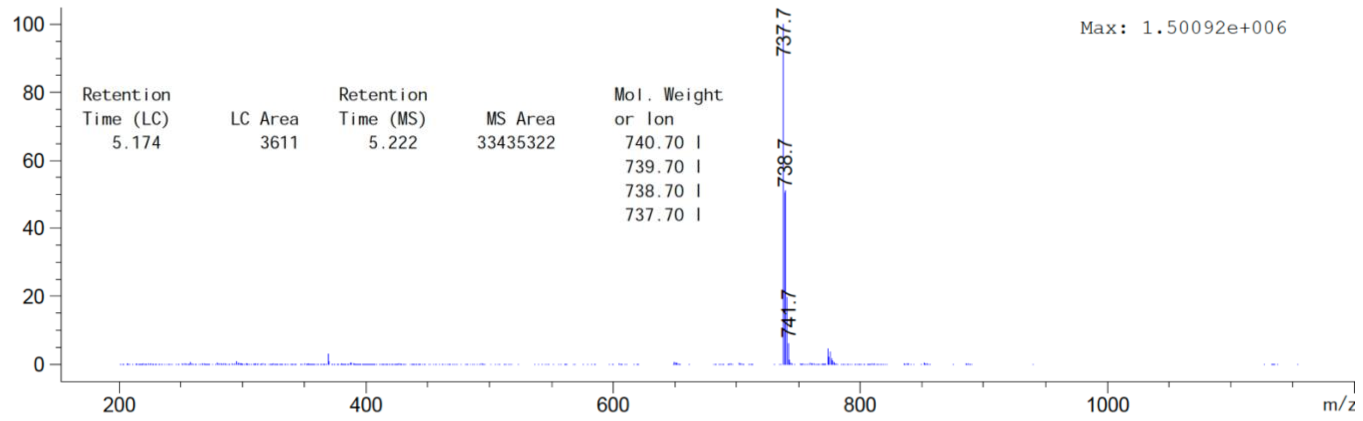

Calculated m/z for **JF$_{635}$-HTL:** 737.29 (100.0%), 738.29 (48.0%), 739.28 (35.4%), 740.29 (16.2%), 739.29 (12.4%), 741.29 (4.3%), 740.28 (2.0%), 741.28 (1.3%), 740.30 (1.2%), 738.28 (1.1%)

Figure S23. SDS-PAGE gel of purified HaloTag (36 kDa) and psHaloTag (52 kDa) proteins.

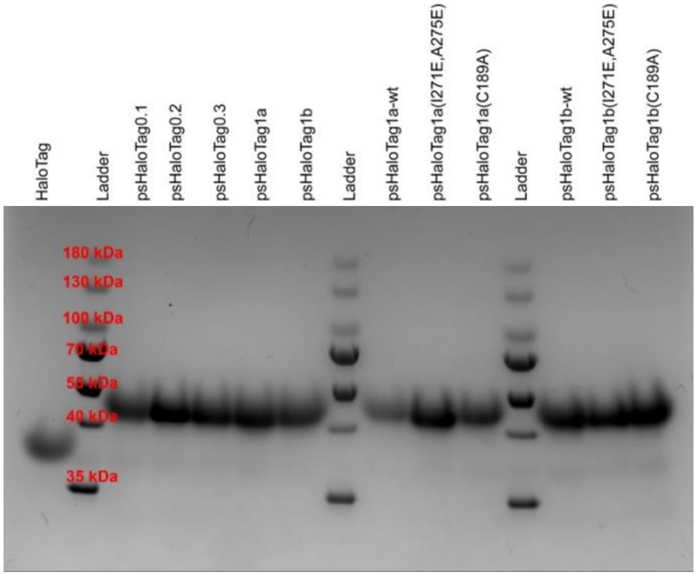

Figure S24. Mass photometry of purified psHaloTag variants.

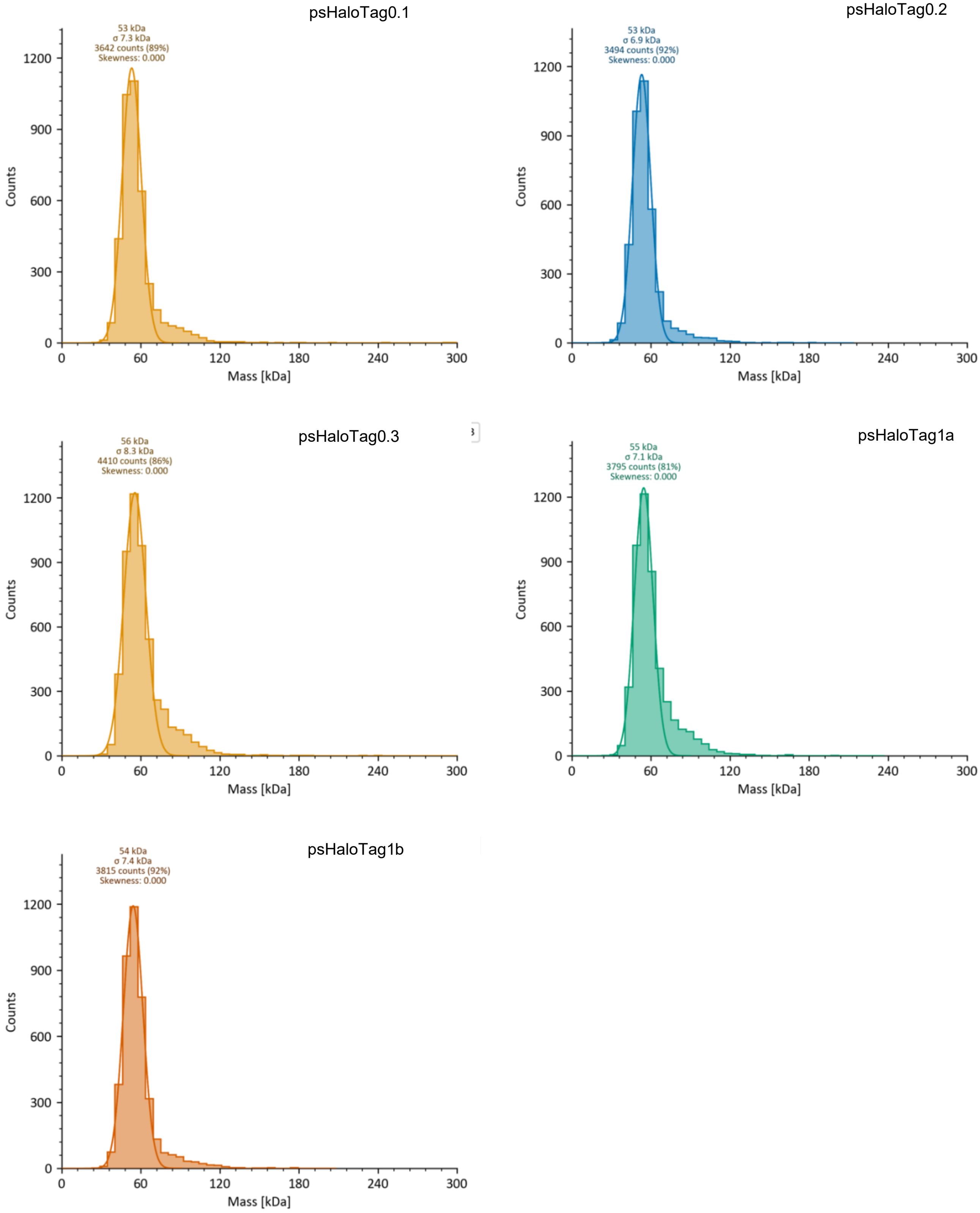

Figure S24. continued

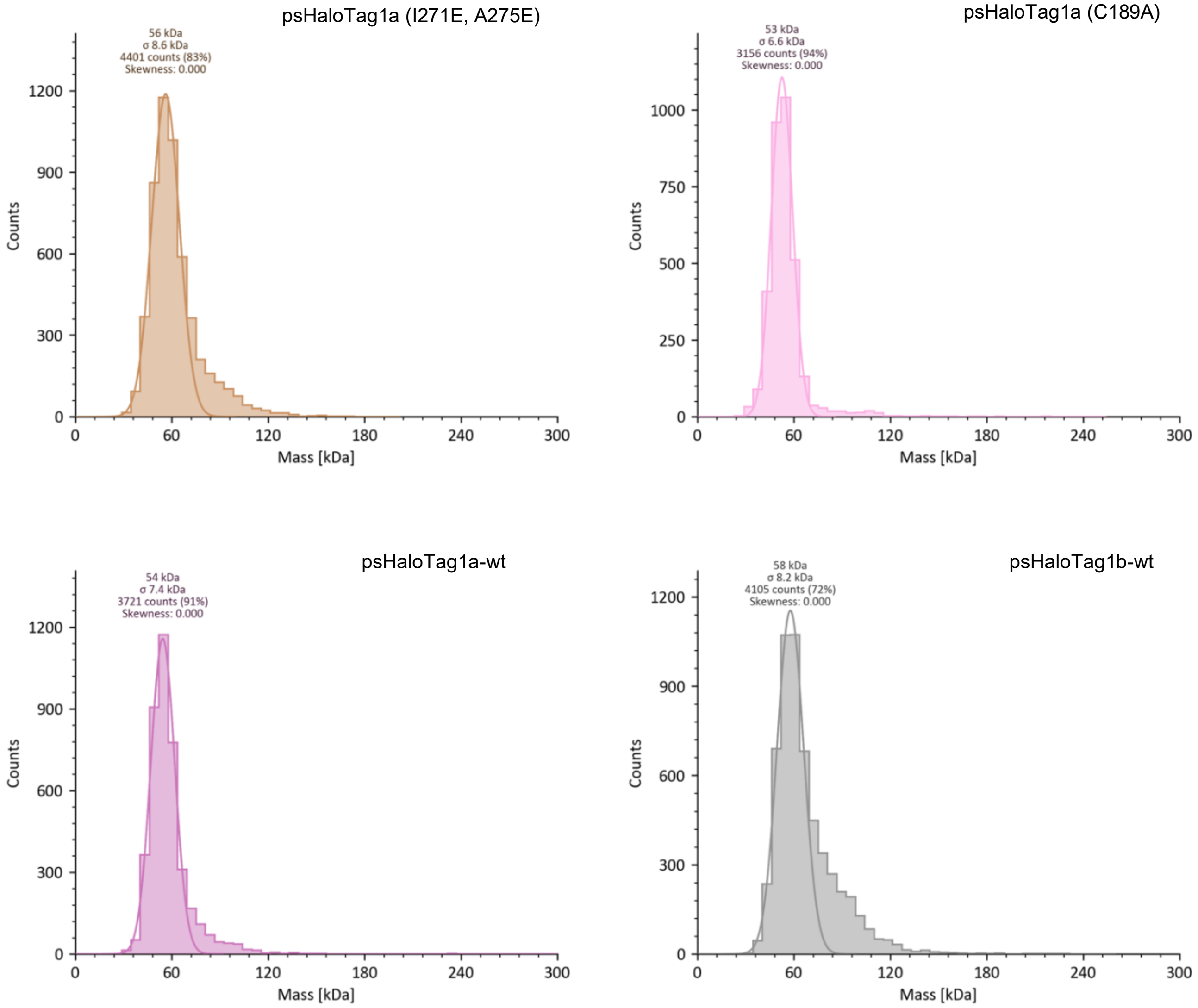

Supplementary Tables

Table S1. Amino acid sequences of psHaloTag constructs. Black = HaloTag, yellow = sAsLOV2, green = inserted amino acid, red = mutation, blue = linker

**HaloTag7**:<br>GIGTGFPFDPHYVEVLGERMHYVDVGPRDGTPVLFLHGNPTSSYVWRNIIPHVAPTHRCIAPDLIGMGKSDKPDLGYFFDDHVRFMDAFIEALG<br>LEEVVLVIHDWGSALGFHWAKRNPERVKGIAFMEFIRPIPTWDEWPEFARETFQAFRTTDVGRKLIIDQNVFIEGTLPMGVVRPLTEVEMDHYR<br>EPFLNPVDREPLWRFPNELPIAGEPANIVALVEEYMDWLHQSPVPKLLFWGTPGVLIPPAEAARLAKSLPNCKAVDIGPGLNLLQEDNPDLIGSEI<br>ARWLSTLEI
**psHaloTag0.1:**<br>GIGTGFPFDPHYVEVLGERMHYVDVGPRDGTPVLFLHGNPTSSYVWRNIIPHVAPTHRCIAPDLIGMGKSDKPDLGYFFDDHVRFMDAFIEALG<br>LEEVVLVIHDWGSALGFHWAKRNPERVKGIAFMEFIRPIPTWDEWPEGLERIEKNFIITDPRLPDNPIIFASDSFLQLTEYSREEILGRNCRFLQGP<br>ETDRATVRKIRDAIDNQTEVTVQLINYTKSGKKFWNLFHLQPMRDQKGDVQYFIGVQLDGTEHVRDAAEREGVMLIKKTAENIDEAAGFARETF<br>QAFRTTDVGRKLIIDQNVFIEGTLPMGVVRPLTEVEMDHYREPFLNPVDREPLWRFPNELPIAGEPANIVALVEEYMDWLHQSPVPKLLFWGTP<br>GVLIPPAEAARLAKSLPNCKAVDIGPGLNLLQEDNPDLIGSEIARWLSTLEI
**psHaloTag0.2:**<br>GIGTGFPFDPHYVEVLGERMHYVDVGPRDGTPVLFLHGNPTSSYVWRNIIPHVAPTHRCIAPDLIGMGKSDKPDLGYFFDDHVRFMDAFIEALG<br>LEEVVLVIHDWGSALGFHWAKRNPERVKGIAFMEFIRPIPTWDEWPEFAGLERIEKNFIITDPRLPDNPIIFASDSFLQLTEYSREEILGRNCRFLQ<br>GPETDRATVRKIRDAIDNQTEVTVQLINYTKSGKKFWNLFHLQPMRDQKGDVQYFIGVQLDGTEHVRDAAEREGVMLIKKTAENIDEAAPFARE<br>TFQAFRTTDVGRKLIIDQNVFIEGTLPMGVVRPLTEVEMDHYREPFLNPVDREPLWRFPNELPIAGEPANIVALVEEYMDWLHQSPVPKLLFWG<br>TPGVLIPPAEAARLAKSLPNCKAVDIGPGLNLLQEDNPDLIGSEIARWLSTLEI
**psHaloTag0.3**:<br>GIGTGFPFDPHYVEVLGERMHYVDVGPRDGTPVLFLHGNPTSSYVWRNIIPHVAPTHRCIAPDLIGMGKSDKPDLGYFFDDHVRFMDAFIEALG<br>LEEVVLVIHDWGSALGFHWAKRNPERVKGIAFMEFIRPIPTWDEWPWFAGLERIEKNFIITDPRLPDNPIIFASDSFLQLTEYSREEILGRNCRFLQ<br>GPETDRATVRKIRDAIDNQTEVTVQLINYTKSGKKFWNLFHLQPMRDQKGDVQYFIGVQLDGTEHVRDAAEREGVMLIKKTAENIDEAAPFARE<br>TFQAFRTTDVGRKLIIDQNVFIEGTLPMGVVRPLTEVEMDHYREPFLNPVDREPLWRFPNELPIAGEPANIVALVEEYMDWLHQSPVPKLLFWG<br>TPGVLIPPAEAARLAKSLPNCKAVDIGPGLNLLQEDNPDLIGSEIARWLSTLEI
**psHaloTag1a**:<br>GIGTGFPFDPHYVEVLGERMHYVDVGPRDGTPVLFLHGNPTSSYVWRNIIPHVAPTHRCIAPDLIGMGKSDKPDLGYFFDDHVRFMDAFIEALG<br>LEEVVLVIHDWGSALGFHWAKRNPERVKGIAFMEFIRPIPTWDEWPWFAGLERIEKNFIITDPRLPDNPIIFASDSFLQLTEYSREEILGRNCRFLQ<br>GPETDRATVRKIRDAIDNQTEVTVQLINYTKSGKKFWNLFHLQPMRDQKGDVQYFIGVQLDGTEHVRDAAEREGVMLIKKTAENIDEAAPFWRE<br>TFQAFRTTDVGRKLIIDQNVFIEGTLPMGVVRPLTEVEMDHYREPFLNPVDREPLWRFPNELPIAGEPANIVALVEEYMDWLHQSPVPKLLFWG<br>TPGVLIPPAEAARLAKSLPNCKAVDIGPGLNLLQEDNPDLIGSEIARWLSTLEI
**psHaloTag1a-wt:**<br>GIGTGFPFDPHYVEVLGERMHYVDVGPRDGTPVLFLHGNPTSSYVWRNIIPHVAPTHRCIAPDLIGMGKSDKPDLGYFFDDHVRFMDAFIEALG<br>LEEVVLVIHDWGSALGFHWAKRNPERVKGIAFMEFIRPIPTWDEWPWFAGLERIEKNFVITDPRLPDNPIIFASDSFLQLTEYSREEILGRNCRFL<br>QGPETDRATVRKIRDAIDNQTEVTVQLINYTKSGKKFWNLFHLQPMRDQKGDVQYFIGVQLDGTEHVRDAAEREGVMLIKKTAENIDEAAPFWR<br>ETFQAFRTTDVGRKLIIDQNVFIEGTLPMGVVRPLTEVEMDHYREPFLNPVDREPLWRFPNELPIAGEPANIVALVEEYMDWLHQSPVPKLLFW<br>GTPGVLIPPAEAARLAKSLPNCKAVDIGPGLNLLQEDNPDLIGSEIARWLSTLEI
**psHaloTag1b:**<br>GIGTGFPFDPHYVEVLGERMHYVDVGPRDGTPVLFLHGNPTSSYVWRNIIPHVAPTHRCIAPDLIGMGKSDKPDLGYFFDDHVRFMDAFIEALG<br>LEEVVLVIHDWGSALGFHWAKRNPERVKGIAFMEFIRPIPTWDEWPWFAGLERIEKNFIITDPRLPDNPIIFASDSFLQLTEYSREEILGRNCRFLQ<br>GPETDRATVRKIRDAIDNQTEVTVQLINYTKSGKKFWNLFHLQPMRDQKGDVQYFIGVQLDGTEHVRDAAEREGVMLIKKTAENIDEAAPFLRE<br>TFQAFRTTDVGRKLIIDQNVFIEGTLPMGVVRPLTEVEMDHYREPFLNPVDREPLWRFPNELPIAGEPANIVALVEEYMDWLHQSPVPKLLFWG<br>TPGVLIPPAEAARLAKSLPNCKAVDIGPGLNLLQEDNPDLIGSEIARWLSTLEI

psHaloTag1b-wt:
GIGTGFPFDPHYVEVLGERMHYVDVGPRDGTPVLFLHGNPTSSYVWRNIIPHVAPTHRCIAPDLIGMGKSDKPDLGYFFDDHVRFMDAFIEALG
LEEVVLVIHDWGSALGFHWAKRNPERVKGIAFMEFIRPIPTWDEWPWFAGLERIEKNFVITDPRLPDNPIIFASDSFLQLTEYSREEILGRNCRFL
QGPETDRATVRKIRDAIDNQTEVTVQLINYTKSGKKFWNLFHLQPMRDQKGDVQYFIGVQLDGTEHVRDAAEREGVMLIKKTAENIDEAAPFLR
ETFQAFRTTDVGRKLIIDQNVFIEGTLPMGVVRPLTEVEMDHYREPFLNPVDREPLWRFPNELPIAGEPANIVALVEEYMDWLHQSPVPKLLFW
GTPGVLIPPAEAARLAKSLPNCKAVDIGPGLNLLQEDNPDLIGSEIARWLSTLEI

psHaloTag1a(C189A):
GIGTGFPFDPHYVEVLGERMHYVDVGPRDGTPVLFLHGNPTSSYVWRNIIPHVAPTHRCIAPDLIGMGKSDKPDLGYFFDDHVRFMDAFIEALG
LEEVVLVIHDWGSALGFHWAKRNPERVKGIAFMEFIRPIPTWDEWPWFAGLERIEKNFIITDPRLPDNPIIFASDSFLQLTEYSREEILGRNARFLQ
GPETDRATVRKIRDAIDNQTEVTVQLINYTKSGKKFWNLFHLQPMRDQKGDVQYFIGVQLDGTEHVRDAAEREGVMLIKKTAENIDEAAPFWRE
TFQAFRTTDVGRKLIIDQNVFIEGTLPMGVVRPLTEVEMDHYREPFLNPVDREPLWRFPNELPIAGEPANIVALVEEYMDWLHQSPVPKLLFWG
TPGVLIPPAEAARLAKSLPNCKAVDIGPGLNLLQEDNPDLIGSEIARWLSTLEI

psHaloTag1a(I271E, A275E):
GIGTGFPFDPHYVEVLGERMHYVDVGPRDGTPVLFLHGNPTSSYVWRNIIPHVAPTHRCIAPDLIGMGKSDKPDLGYFFDDHVRFMDAFIEALG
LEEVVLVIHDWGSALGFHWAKRNPERVKGIAFMEFIRPIPTWDEWPWFAGLERIEKNFIITDPRLPDNPIIFASDSFLQLTEYSREEILGRNCRFLQ
GPETDRATVRKIRDAIDNQTEVTVQLINYTKSGKKFWNLFHLQPMRDQKGDVQYFIGVQLDGTEHVRDAAEREGVMLEKKTEENIDEAAPFWR
ETFQAFRTTDVGRKLIIDQNVFIEGTLPMGVVRPLTEVEMDHYREPFLNPVDREPLWRFPNELPIAGEPANIVALVEEYMDWLHQSPVPKLLFW
GTPGVLIPPAEAARLAKSLPNCKAVDIGPGLNLLQEDNPDLIGSEIARWLSTLEI

psHaloTag1b(C189A):
GIGTGFPFDPHYVEVLGERMHYVDVGPRDGTPVLFLHGNPTSSYVWRNIIPHVAPTHRCIAPDLIGMGKSDKPDLGYFFDDHVRFMDAFIEALG
LEEVVLVIHDWGSALGFHWAKRNPERVKGIAFMEFIRPIPTWDEWPWFAGLERIEKNFIITDPRLPDNPIIFASDSFLQLTEYSREEILGRNARFLQ
GPETDRATVRKIRDAIDNQTEVTVQLINYTKSGKKFWNLFHLQPMRDQKGDVQYFIGVQLDGTEHVRDAAEREGVMLIKKTAENIDEAAPFLRE
TFQAFRTTDVGRKLIIDQNVFIEGTLPMGVVRPLTEVEMDHYREPFLNPVDREPLWRFPNELPIAGEPANIVALVEEYMDWLHQSPVPKLLFWG
TPGVLIPPAEAARLAKSLPNCKAVDIGPGLNLLQEDNPDLIGSEIARWLSTLEI

psHaloTag1b(I271E, A275E):
GIGTGFPFDPHYVEVLGERMHYVDVGPRDGTPVLFLHGNPTSSYVWRNIIPHVAPTHRCIAPDLIGMGKSDKPDLGYFFDDHVRFMDAFIEALG
LEEVVLVIHDWGSALGFHWAKRNPERVKGIAFMEFIRPIPTWDEWPWFAGLERIEKNFIITDPRLPDNPIIFASDSFLQLTEYSREEILGRNCRFLQ
GPETDRATVRKIRDAIDNQTEVTVQLINYTKSGKKFWNLFHLQPMRDQKGDVQYFIGVQLDGTEHVRDAAEREGVMLEKKTEENIDEAAPFLRE
TFQAFRTTDVGRKLIIDQNVFIEGTLPMGVVRPLTEVEMDHYREPFLNPVDREPLWRFPNELPIAGEPANIVALVEEYMDWLHQSPVPKLLFWG
TPGVLIPPAEAARLAKSLPNCKAVDIGPGLNLLQEDNPDLIGSEIARWLSTLEI

Table S2. Apparent kinetic rate constants of the photoswitching (upon illumination at 450 nm) and thermal relaxation (in the dark at room temperature) of psHaloTag variants labelled with **JF_{635}-HTL**, for both the FMN cofactor signal and **JF_{635}** signal. The corresponding full kinetic profiles can be found in Figure S5. n.m.: not measured.

	Illumination at 450 nm		Thermal relaxation (dark, room temperature)			
Construct	$k_{ON}(JF_{635})$ (min^{-1})	$t_{1/2, ON}(JF_{635})$ (min)	k_{relax}(FMN) (min^{-1})	$t_{1/2,relax}$(FMN) (min)	$k_{relax}(JF_{635})$ (min^{-1})	$t_{1/2,relax}(JF_{635})$ (min)
psHaloTag0.1	n.m.	n.m.	0.091	7.6	0.103	6.8
psHaloTag0.2	n.m.	n.m.	0.088	7.9	n.m.	n.m.
psHaloTag0.3	0.030	24.1	0.090	7.7	0.055	12.7
psHaloTag1a	0.048	14.3	0.088	7.9	0.036	19.5
psHaloTag1a-wt	0.039	17.0	1.20	0.6	0.117	5.9
psHaloTag1b	0.307	2.3	0.104	6.7	0.072	9.7
psHaloTag1b-wt	0.332	2.1	1.22	0.6	0.378	1.8

Table S3. Crystallographic data and refinement statistics. Data for highest-resolution shell are shown parenthesis.

	**psHaloTag1a$_{635}$**
**Data collection**	
Space group	C121
Resolution (Å)	70.36 – 2.4 (2.49 – 2.4)
Unique reflection	40131 (3989)
a, b, c (Å)	167.74, 78.02, 81.81
α, β, γ (°)	90.0, 103.9, 90.0
R_{merge}	0.262 (1.228)
R_{pim}	0.106 (0.491)
Mean (I/σ(I))	5.6 (1.04)
Multiplicity	7.0 (7.1)
Completeness (%)	99.55 (99.60)
$CC_{1/2}$	0.99 (0.58)
**Refinement**	
R_{work} (%)	21.31 (30.16)
R_{free} (%)	25.23 (32.81)
RMSD Bond length (Å)	0.009
RMSD Bond angle (°)	1.35
Ramachandran favored (%)	95.35
Ramachandran allowed (%)	4.65
Ramachandran outliers (%)	0.0
Rotamer outliers (%)	2.26
Clashscore	7.01
Average B factor (Å^2)	39.91
Protein	39.75
Ligands	43.83
Solvent	40.50
PDB identifier	9HKF

Table S4. List and composition of buffers used in this work.

Buffer	Composition
Spectroscopy buffer	20 mM Tris-HCl pH 7.4, 100 mM NaCl, 0.1 mg·ml^{-1} CHAPS
Lysis buffer	20 mM Tris-HCl pH 7.4, 300 mM NaCl, 1 mM PMSF, 0.01 mg·mL^{-1} DNase [for screening assays 2 mg·mL^{-1} lysozyme/for protein purification ½ tablet of protease inhibitor (cOmplete)]
Ni-NTA chromatography wash buffer	20 mM Tris HCl pH 7.4, 300 mM NaCl, 10 mM imidazole
Ni-NTA chromatography elution buffer	20 mM Tris-HCl pH 7.4, 300 mM NaCl, 500 mM imidazole (150 mM imidazole for protein crystallization)
Dialysis buffer	20 mM Tris-HCl pH 7.4, 100 mM NaCl, 5 mM imidazole, 2 mM β-mercaptoethanol
Size exclusion chromatography (SEC) buffer/Storage buffer	20 mM Tris-HCl pH 7.4, 100 mM NaCl
DMEM	DMEM high glucose (4.5 g·L^{-1}), 10% fetal bovine serum, 2 mM L-glutamine, 1 mM sodium pyruvate, 100 U·mL^{-1} penicillin, 100 µg·mL^{-1} streptomycin
Imaging buffer (microscopy)	DMEM without phenol red high glucose (4.5 g·L^{-1}), 10% fetal bovine serum, 1 mM sodium pyruvate, 1 mM L-glutamine

Table S5. Thermal stability of purified proteins determined by differential scanning fluorimetry.

Protein variant	T_{onset} (°C)	T_m (°C)	T_{agg} (°C)
psHaloTag0.1	34.8	46.4	42.5
psHaloTag0.2	37.9	48.5	41.2
psHaloTag0.3	35.3	46.3	40.6
psHaloTag1a	40.8	48.6	39.7
psHaloTag1b	37.6	48.6	40.7
psHaloTag1a-wt	42.2	49.8	41.9
psHaloTag1b-wt	39.1	48.7	40.7
psHaloTag1a (I271E, A275E)	35.4	45.2	40.4
psHaloTag1a (C189A)	41.4	49.0	40.2
psHaloTag1b (I271E, A275E)	33.9	45.0	44.3
psHaloTag1b (C189A)	37.9	47.6	42.4

Table S6. Plasmids used for cell culture and microscopy.

Cellular target	Construct	Addgene #
Nucleus	pCDNA5_FRT_TO_H2B_HaloTag_T2A_EGFP	135444
	pCDNA5_FRT_TO_H2B_psHaloTag1a_T2A_EGFP	-
	HaloTag-H2B-N-10	-
	psHaloTag1a-H2B-N-10	-
Mitochondria	pCDNA5_FRT_TO_TOMM20_HaloTag_T2A_EGFP	135443
	pCDNA5_FRT_TO_TOMM20_ psHaloTag1a _T2A_EGFP	-
Actin cytoskeleton	pCDNA5_FRT_TO_LifeAct_ HaloTag _T2A_EGFP	135445
	pCDNA5_FRT_TO_LifeAct_ psHaloTag1a _T2A_EGFP	-

General Experimental Information

Chemicals, reagents and buffers

JF_{635}-HTL was synthesized as previously described, and ^{1}H NMR and HPLC confirmed identity and purity of the compound (Figure S22).[2] The HaloTag ligands **JF_{525}-HTL**,[2] **JF_{526}-HTL**,[3] **JF_{585}-HTL**,[2] **JF_{626}-HTL**,[4] **JF_{629}-HTL**,[4] **JF_{630}-HTL**,[4] **JF_{639}-HTL**,[4] **JF_{711}-HTL**[5] were gifted by the Lavis group (Janelia Research Campus, HHMI). The HaloTag ligands **MaP_{618}-HTL**[6] and **MaP_{700}-HTL**[6] were gifted by the Johnsson group (Max Planck Institute for Medical Research). The self-blinking **JF_{630}b-HTL**[9] and **JF_{635}b-HTL**[9] were gifted by the Lavis Group (Janelia Research Campus, HHMI).

Commercial reagents were obtained from reputable suppliers and used as received.
The composition of buffers used in this work can be found in Table S4.

UV-Vis and Fluorescence Spectroscopy

All measurements were performed at room temperature (23 ± 2°C). Fluorophore-ligands were initially prepared as 1 mM stock solutions in DMSO (spectroscopy grade) and subsequently diluted in appropriate solvents and buffers to ensure that the final DMSO concentration did not exceed 1% v/v. Unless otherwise specified, spectroscopic measurements with purified proteins were performed in aqueous buffer containing 20 mM Tris-HCl and 100 mM NaCl at pH 7.4, to which 0.1 mg·mL^{-1} 3-((3-cholamidopropyl)dimethylammonio)-1-propanesulfonate (CHAPS) was added. Spectroscopic measurements were conducted using 1-cm path-length quartz cuvettes (Hellma Suprasil Quartz), or in 96-well plates (Nunc™ MicroWell™ 96-Well, Nunclon Delta-Treated, Flat-Bottom Microplate).

Absorption spectra were recorded using a Cary Model 60 spectrophotometer (Agilent). Absorption spectra, including maximum absorption wavelength (λ_{max}), extinction coefficient (ε), and maximum emission wavelength (λ_{em}), were measured in triplicates. Spectra were recorded at 5 µM dye concentration, unless otherwise stated. For measurements in the presence of proteins, the dye ligand was incubated with 1.5 equivalents (eq) of purified protein for at least 3 h in the dark at room temperature.

Fluorescence spectra were recorded using a JASCO spectrofluorometer (FP-8500), at 1 µM dye concentration of and 1.5 µM protein concentration, unless otherwise stated. Fluorescence intensity measurements at a single wavelength were performed on a plate reader (TECAN Infinite M1000 Pro fluorescence plate reader or TECAN Spark microplate reader) equipped with appropriate filters and a monochromator.

Data analysis and graph plotting were carried out using Prism (GraphPad).

The following notations are used:

λ_{max} (nm)	Wavelength at maximal absorption
λ_{em} (nm)	Wavelength at maximal fluorescence emission
ε (M^{-1}·cm^{-1})	Molar extinction coefficient
Φ_F	Fluorescence quantum yield
A/A_0	Change in absorbance

F/F_0	Change in fluorescence
k_{ON} (min^{-1})	Apparent rate constant of the turn-on photoinduced process
k_{relax} (min^{-1})	Apparent rate constant of thermal relaxation
$t_{1/2,ON}$ (min)	Half-life of the turn-on, photoinduced process. Determined as $t_{1/2,ON} = ln2/ k_{ON}$.
$t_{1/2, relax}$ (min)	Half-life of the thermal relaxation. Determined as $t_{1/2, relax} = ln2/k_{relax}$.

Extinction coefficient measurements

Purified protein solutions were labelled with 0.7 eq **JF$_{635}$-HTL** in aqueous buffer (20 mM Tris-HCl, 100 mM NaCl, 0.1 mg·mL^{-1} CHAPS, pH 7.4) for 3 h at room temperature, in the dark. The absorption spectra of labelled protein solutions at different concentrations (1 µM, 1.25 µM, 1.5 µM, 2.0 µM, 2.5 µM, 3 µM, 4 µM, 5 µM, and 6 µM) were recorded before and after illumination at 450 nm. The spectra were baseline corrected, and the maximum absorption values were plotted against concentration. The extinction coefficient (ε) at λ_{max} was determined by linear curve fitting using the Beer-Lambert law.

Quantum yield measurements

Fluorescence quantum yields (Φ_F) were measured using an absolute quantum yield measurement system (Quantaurus, C11347, Hamamatsu). Measurements were carried out using dilute samples (A < 0.1). Self-absorption corrections were performed using the Quantaurus software. Purified protein solutions (1.5 µM) were labelled with **JF$_{635}$-HTL** (1 µM) in aqueous buffer (20 mM Tris-HCl, 100 mM NaCl, 0.1 mg·mL^{-1} CHAPS, pH 7.4) for 3 h at room temperature in the dark before measurements. Afterward, the samples were illuminated at 450 nm until complete photoswitching was achieved (assessed by UV-Vis spectroscopy), and the quantum yield of the ON state was measured.

Photoswitching

Photoswitching was performed using a LED illumination device, designed and built by the Electronical Workshop at EMBL Heidelberg (Figure S3). They consist of two parts: the control board and the LED board, comprised of 24 LEDs at 450 nm (LED450-03, Roithner LaserTechnik). The illumination device could be controlled using the RealTerm Serial terminal program, with the following parameters adjustable: power intensity, exposure time ON and OFF (in ms), and number of ON/OFF cycles. For all experiments, the maximum power intensity of the system was used, corresponding to 2.6 mW·cm^{-2} in our setup.

Thermal relaxation kinetics

Thermal relaxation kinetics measurements were performed in a microplate reader (TECAN Infinite M1000 microplate reader) at room temperature, in the dark, in 96-well plates, in aqueous buffer (20 mM Tris-HCl, 100 mM NaCl, 0.1 mg·mL^{-1} CHAPS, pH 7.4). The plate was illuminated at 450 nm until the maximal ON state has been reached (assessed by fluorescence measurement). The fluorescence intensity values of the fluorophore ($\lambda_{max}/\lambda_{em}$ = 640/660 nm) and the FMN ($\lambda_{max}/\lambda_{em}$ = 450/525 nm) were recorded every minute for a duration of 2 h. The resulting experimental data were fitted to a one-phase exponential decay function to determine the FMN fluorescence increase rate and the dye fluorescence decrease rate as a proxy for the FMN-Cys adduct lifetime and the conformational relaxation of the HaloTag binding cavity. The following equation was used:

$$F(t) = (F_{MAX} - F_0) + exp(-k_{relax} * t) + F_0$$

Where:

t is the time in min.
F(t) is the fluorescence intensity of the sample at t.
F_{MAX} is the fluorescence intensity at t = 0 (illuminated state).
F_0 is the fluorescence intensity in the dark state.
k_{relax} is the apparent rate constant of the thermal relaxation, in min^{-1}.

pH stability

Aqueous buffers of varying pH (2-11) were prepared using Na_2HPO_4 (200 mM) and sodium citrate (100 mM) for pH 2-7 and Na_2CO_3 (100 mM) and $NaHCO_3$ (100 mM) for pH 8-11, respectively. Purified psHaloTag 1a prelabelled with **JF_{635}-HTL** (1 µM) was incubated with the respective pH-adjusted buffer for 3 h and the emissions of FMN ($\lambda_{exc}/\lambda_{em}$ = 450/525 nm) and **JF_{635}** ($\lambda_{exc}/\lambda_{em}$ = 640/660 nm) before and after illumination (50 min, 450 nm, 2.6 mW·cm^{-2}) were measured on a microplate reader (TECAN Spark). Following illumination, the thermal relaxation was determined by recording the emission of FMN and **JF_{635}** in time intervals of 5 min for a total duration of 90 min. The thermal relaxation constants were determined as described above.

Binding kinetics

Binding kinetics measurements were performed in Cary Model 60 spectrophotometer (Agilent) at room temperature, in the dark, 1-cm path-length quartz cuvettes (Hellma Suprasil Quartz), in aqueous buffer (20 mM Tris-HCl, 100 mM NaCl, 0.1 mg·mL^{-1} CHAPS, pH 7.4). To **JF_{635}-HTL** (5 µM) in aqueous buffer was psHaloTag1a (7.5 µM) added in the dark and quickly mixed. The absorption spectra were recorded every minute for a duration of 60 minutes. The resulting experimental data were fitted to a mono exponential function. The following equation was used:

$$F(t) = (F_{MAX} - F_0) * (1 - exp(-k_{app} * t)) + F_0$$

Where:
t is the time in min.
F(t) is the absorption intensity of the sample at t.
F_{MAX} is the absorption intensity in the fully bound state
F_0 is the absorption intensity prior binding
k_{app} is the apparent rate constant of binding, in min^{-1}.

Cloning, Screening, Protein Expression and Purification

Cloning

The pET51b(+) vector (pET51b(+)-HaloTag7, Addgene #167266) was kindly provided by K. Johnsson (Max Planck Institute for Medical Research) and was used for cloning and protein expression in *E. coli* BL21(DE3). Proteins were N-terminally tagged with Hisx10, followed by a tobacco etch virus (TEV) protease cleavage site (ENLYFQ|G). For *in vitro* screening, YFP was fused to the C-terminus of psHaloTag constructs. For crystallization purposes, the pCoofy1 vector (Addgene #43974, kindly provided by the Protein Expression and Purification Core Facility at EMBL Heidelberg) was selected. In this case, proteins were N-terminally tagged with Hisx6, followed by the HRV 3C protease cleavage site (LEVLFQ|GP). For mammalian cell experiments, the pcDNA5/FRT/TO vector (ThermoFisher Scientific) containing HaloTag7

fused to different subcellular targeting motifs (H2B, TOMM20, or LifeAct) were used for cloning and imaging (kindly provided by K. Johnsson (Max Planck Institute for Medical Research)).

The amino acid sequences of the isolated psHaloTag variants can be found in Table S1. For cloning, the psHaloTag nucleic acid sequence was amplified by polymerase chain reaction (PCR) and inserted into the desired vector backbone via Gibson assembly (New England Biolabs) and purified by DNA Clean & Concentrator-5 (Zymo Research). In case of point mutations (site-directed or site-saturation mutagenesis for library generation) the desired codon or a NNS degenerate codon was used at the 5´-of the forward primer for construct amplification by PCR. Generated products were then ligated using KLD mix (New England Biolabs) and purified by DNA Clean & Concentrator-5 (Zymo Research). Plasmid DNA was subsequently electroporated in *E. coli* Turbo cells (New England Biolabs) plated on LB agar plates with appropriate antibiotics and incubated at 37°C overnight. In all cases, plasmid DNA was extracted from individual bacterial colonies (QIAwave Plasmid Miniprep Kit, Qiagen), and the sequences of generated plasmids were verified by Sanger sequencing (Eurofins Genomics).

Site-saturation mutagenesis (SSM) for library generation

Site-saturation mutagenesis was performed using NNS degenerate codon at the 5´-of the forward primer for construct amplification by PCR. Generated products were then ligated using KLD mix (New England Biolabs) and purified by DNA Clean & Concentrator-5 (Zymo Research). Generated DNA constructs by site-saturation mutagenesis were electroporated into *E. coli* strain BL21(DE3). The cells were plated on agar LB plates bearing 100 µg·mL^{-1} ampicillin and 200 µM isopropyl-β-D-thiogalactopyranoside (IPTG) and incubated at 37°C overnight. Diversity was proven by plasmid extraction from a selective liquid LB culture (in autoinduction medium containing 100 µg·mL^{-1} ampicillin) grown at 37°C overnight. The plasmid libraries were sequenced using the Sanger method (Eurofins Genomics) to verify the proper incorporation of random codons at the desired position. Single colonies were used to inoculate 1 mL selective auto-induction medium containing 100 µg·mL^{-1} ampicillin in a U-bottom-shaped 96-deep well plates (ThermoFisher Scientific). The autoinduction medium was prepared in-house.[10]

Three wells of the 96-well plate were reserved for parental HaloTag7 as a positive control, three wells for the parent psHaloTag construct as a comparison for F/F_0, and three wells for sAsLOV2 as a negative control for the binding assay. The bacterial cultures were incubated at room temperature for 48 h with shaking at 350 rpm. Subsequently, 40 µL of the cultures were used to inoculate 40 µL of sterile LB medium containing 30% glycerol in a 384-well plate and kept at -70°C as archives. The rest of the cultures were harvested by centrifugation (3500 rpm, 15 min, 4°C). The bacterial pellets in each well were resuspended in 500 µL lysis buffer (20 mM Tris-HCl, 300 mM NaCl, 2 mg·mL^{-1} lysozyme, 0.01 mg·mL^{-1} DNase) and were submitted to five cycles of freeze/thawing with liquid nitrogen before resuspension and incubating at room temperature overnight at 350 rpm shaking. The obtained cell lysate was cleared by centrifugation (3500 rpm, 20 min, 4°C). The cleared supernatant used for the screening assay.

Screening assay

Cleared supernatants from cell lysates (95 µL) were transferred into a 96-well plate (Nunc™ MicroWell™ 96-Well, Nunclon Delta-Treated, Flat-Bottom Microplate). Basal **JF$_{635}$** ($\lambda_{exc}/\lambda_{em}$ = 640/660 nm) and YFP ($\lambda_{exc}/\lambda_{em}$ = 515/530 nm) fluorescence were measured in a microplate reader (TECAN Infinite M1000 microplate reader) before adding **JF$_{635}$-HTL** in each well (5 µL of DMSO stock solution, 50 nM final concentration). Wells with a protein concentration < 100 nM were discarded from the analysis. After incubation at room temperature in the dark for 4 h while shaking, fluorescence intensities were measured to obtain the F_0 values. The plate was then illuminated three times for 3 min at 450 nm (LED box, 2.6

mW·cm^{-2}), and the fluorescence intensities were measured subsequently after each illumination. To verify and quantify binding of the fluorophore to the protein, a second plate was prepared in the same way but instead of illuminating it, purified HaloTag protein (100 nM final concentration) was added. After 30 min incubation, the fluorescence intensities were measured again. Efficient ligand binding was confirmed if no increase in fluorescence intensity was observed following addition of HaloTag protein, ensuring that no free ligand was left unbound in the lysate. The control wells were used to determine the mean and SD of YFP and **JF$_{635}$** fluorescence intensities of the parental proteins as well as F/F_0 upon illumination. Wells with **JF$_{635}$** fluorescence intensities showing a higher F/F_0 upon illumination than the control were selected for further characterization. A representation of the screening pipeline can be found in Figure S2.
Plasmids of selected wells were retrieved from stored bacterial archives and sequenced. Their photoswitching properties were then confirmed, with selected variants expressed and lysed from 50 mL selective LB cultures (as described above). Each protein (1 µM) was labelled with **JF$_{635}$-HTL** (50 nM) in 100 µL buffer in a 96-well plate and incubated for 4 h at room temperature in the dark. The **JF$_{635}$** and YFP fluorescence intensities were measured as previously described. Measurements were performed in triplicates. Mean and 90% confidence intervals were calculated for every variant and compared with those of the parental protein. Variants with significant changes in **JF$_{635}$** fluorescence intensity upon illumination (450 nm, 2.6 mW·cm^{-2}) compared with the parental protein were selected for further purification and characterization.

Protein expression and purification

Proteins were expressed using the pET51b(+) plasmids in *E. coli* BL21(DE3) cells. LB cultures containing 100 µg·mL^{-1} ampicillin were grown at 37°C to an optical density at 600 nm (OD600) of 0.4-0.6, induced by the addition of IPTG (1 mM final concentration) for protein expression and further grown at 18°C overnight (shaking 200 rpm). The cells were harvested by centrifugation (6000 rpm, 20 min, 4°C) and lysed by microfluidizer (Microfluidics Microfluidizer, M-110L FluidProcessor) in lysis buffer (20 mM Tris-HCl pH 7.4, 300 mM NaCl, 1 mM PMSF, ½ tablet of protease inhibitor, 0.01 mg·mL^{-1} DNase). The cell lysate was cleared by ultra-centrifugation (35000 rpm, 45 min, 4°C). Proteins were purified using affinity-tag Ni-NTA agarose (Qiagen) and the eluted fractions (20 mM Tris-HCl pH 7.4, 300 mM NaCl, 500 mM imidazole) were then pooled. Size exclusion chromatography (SEC) (HiLoad 16/600 Superdex 200 pg, Cytiva) was performed to obtain the monomeric fraction with elution using SEC buffer (20 mM Tris-HCl, 100 mM NaCl, pH 7.4). This final protein fraction was concentrated (Amicon Ultra Centrifugal Filter, 30 kDa MWCO, Merck) to 100-170 µM and stored in 100 µL aliquots at −70°C. These proteins were also further characterized by NanoDSF (Prometheus NT.Flex, see details below and Table S5), SDS-PAGE gel (Bio-Rad, Mini-PROTEAN® TGX™ stain-free gels, Figure S23), and mass photometry (Refeyn Two^{MP} from Refeyn, Figure S24). NanoDSF and mass photometry measurements were performed by Karine Lapouge at the Protein Expression and Purification Core Facility (EMBL).

Protein stability experiments

The temperature-dependent unfolding, as a proxy for thermal stability, was determined by using differential scanning fluorimetry (DSF), by employing a Prometheus NT.Flex (NanoTemper Technologies GmbH, Munich, Germany) instrument. DSF monitors the unfolding of proteins by detecting temperature-dependent changes in aromatic amino acid fluorescence. Purified psHaloTag samples (10 µL, 0.4–0.5 mg·mL^{-1}) were subjected to a linear temperature increase (20–95°C, 1°C·min^{-1}). The intrinsic tryptophan fluorescence of the protein was monitored continuously (18 data points per minute) at 330 and 350 nm. Unfolding transition midpoints (expressed as melting temperature, Tm) were determined from the first derivative of the

fluorescence ratio (F350/F330) by using the RT.ThermControl Software (NanoTemper Technologies GmbH). Values can be found in Table S5.

Structure Prediction

ColabFold is an accessible tool for predicting protein structures that combines the fast homology search capabilities of MMseqs2 with AlphaFold2 or RoseTTAFold algorithms.[11] ColabFold also gives important confidence measures for evaluating the predicted protein structure: pLDDT (predicted Local Distance Difference Test) and PAE (Predicted Aligned Error). pLDDT scores range from 0 to 100, indicating confidence in the accuracy of individual residues: scores above 90 suggest high confidence, while scores below 70 indicate potential inaccuracies. PAE measures the expected positional error between pairs of residues, with lower values reflecting greater confidence in their spatial arrangement. For 3D protein structure prediction, protein sequences were inputted into ColabFold version 1.5.2 (https://github.com/sokrypton/ColabFold) installed on the local HPC cluster through EasyBuild environment module (https://docs.easybuild.io/version-specific/supported-software/c/ColabFold/) with the help of Federico Marotta (Bork Group, EMBL Heidelberg). For each sequence, five models were generated and ranked by their pLDDT scores. The predicted structures were visualized and analyzed using PyMOL.

X-Ray Crystallography

Protein purification and crystallization

pCoofy1-derived vector with kanamycin antibiotic resistance (Addgene #43974, kindly provided by the Protein Expression and Purification Core Facility at EMBL Heidelberg) was used for protein crystallization purposes. In this case, psHaloTag1a was N-terminally tagged with Hisx6, followed by the HRV 3C protease cleavage site (LEVLFQ|GP). psHaloTag1a was expressed in *E. coli* BL21(DE3) cells. 3 liters LB cultures were grown in the presence of 100 µg·mL^{-1} kanamycin at 37°C to an optical density at 600 nm of 0.4−0.6, induced by the addition of IPTG (1 mM final concentration) and further grown at 18°C overnight (200 rpm). The cells were harvested by centrifugation (6000 rpm, 20 min, 4°C) and lysed by microfluidizer (Microfluidics Microfluidizer, M-110L FluidProcessor) in lysis buffer (20 mM Tris-HCl pH 7.4, 300 mM NaCl, 1 mM PMSF, ½ tablet of protease inhibitor (cOmplete, Roche), 0.01 mg·mL^{-1} DNase (Roche)). The cell lysate was cleared by ultra-centrifugation (35000 rpm, 45 min, 4°C). Proteins were purified using Ni-NTA chromatography (Protino Ni-NTA 5 mL FLPC column, Macherey-Nagel) and the eluted fraction (20 mM Tris-HCl pH 7.4, 300 mM NaCl, 150 mM imidazole) was dialyzed overnight in the presence of His-tagged HRV 3C protease (1:200) in dialysis buffer (50 mM Tris-HCl pH 7.4, 100 mM NaCl, 5 mM imidazole, 2 mM β-mercaptoethanol) followed by a reverse Ni-NTA chromatography. The flow-through fraction was then used for size exclusion chromatography. The obtained monomeric fraction was then incubated with **JF$_{635}$-HTL** (in excess, 2 molar equivalents) overnight at 4°C in SEC buffer containing 1% DMSO. Excess dye was then removed by PD-10 desalting column (Cytiva) and dye-labelled protein was concentrated to 47 mg·mL^{-1} for protein crystallization screens. psHaloTag1a$_{635}$ was screened for crystallization using the sitting drop vapor diffusion method with different commercial crystallization screens. Sample and crystallization conditions were mixed in 96-well plates with two sample concentrations (23 mg·mL^{-1} and 12 mg·mL^{-1} final concentrations) by pipetting 100 nL of sample to 100 nL crystallization buffer using a Mosquito LCP pipetting robot (SPT Labtech). Crystallization was carried out at 20°C in a crystallization incubator (Formulatrix). Diffracting crystals grew in a condition with 0.1 M Bis-Tris propane pH 6.5, 0.2 M sodium

nitrate, 20% (w/v) PEG3350, 10% (v/v) ethylene glycol (Ligand Friendly Screen, Molecular Dimensions). Crystals appeared after 1-5 days, were fished in CrystalCap SPINE HT loops, and frozen with an additional 20 % (v/v) glycerol as cryo-protectant in liquid nitrogen.

X-Ray data collection and model building

psHaloTag1a$_{635}$ X-ray diffraction data set was collected at beamline ID23-2 (ESRF, Grenoble) at 100 K with reflections up to 2.4 Å. The crystal belonged to the C121 space group with unit cell dimensions of 167.7 Å x 78.0 Å x 81.8 Å and 90°, 104°, 90° angles. Reflections on diffraction images were integrated by the ESRF automated processing pipeline with XDS[12] and STARANISO.[13] Subsequently, phases were obtained by molecular replacement using a HaloTag protein model (PDB: 5Y2Y)[14] with PHASER-MR[15] implemented in the PHENIX package.[16] Iterative model building and refinement were performed with Coot[17] and Phenix.refine.[18] The asymmetric unit comprises two molecules of psHaloTag1a$_{635}$. The final structure also contains eleven molecules of glycerol.

Cell Culture and Microscopy

Cell culture

U2OS cells (ATCC) were cultured in Dulbecco's modified Eagle medium (DMEM, high glucose (4.5 g·L^{-1}) with phenol red (Gibco), supplemented with 10% v/v fetal bovine serum (ThermoFisher Scientific), penicillin (100 units·mL^{-1}), streptomycin (100 µg·mL^{-1}, Gibco), 2 mM L-Glutamine (Gibco) and 1 mM sodium pyruvate (Gibco), and maintained at 37°C in a humidified 5% v/v CO_2 environment.

Plasmids used can be found in Table S6. Transient transfections were performed using FUGENE6 (Promega) transfection reagent according to the manufacturer´s recommendations ratio (1 µg plasmid DNA, 3 µl FUGENE6) in OptiMEM (Gibco) and added to wells (Nunclon delta surface 6 well plates, Nunc) with 70−80% cell confluency in imaging buffer (DMEM without phenol red (Gibco), supplemented with glucose (4.5 g·L^{-1}), 10% v/v fetal bovine serum (ThermoFisher Scientific), 2 mM L-Glutamine (Gibco) and 1 mM sodium pyruvate (Gibco)) for 24−48 h. Transfected cells were then trypsinized, counted, and seeded into imaging plates (24 well glass bottom plate with high-performance #1.5 cover glass (Cellvis)). For the SMLM experiment, cells were seeded into ibidi, µ-Dish$^{35\ mm,\ high}$ Glass Bottom, 81158. The labelling and imaging were conducted the following day.

Labelling and sample preparation

Cells were labelled with respective fluorophores (1 µM, 3 h at 37°C, in the dark) in imaging buffer and subsequently washed three to five times with prewarmed imaging buffer before fluorescence imaging.

Colocalization assay

U2OS cells expressing H2B-psHaloTag1a-T2A-EGFP or TOMM20-psHaloTag1a-T2A-EGFP were incubated with 1 µM **JF$_{635}$-HTL** for 2 h and washed 3 times with imaging buffer, prior to cellular marker staining. For nuclear staining, cells were incubated with 10 µM Hoechst 33342 (Thermo Scientific) for 10 min in imaging buffer. For mitochondrial staining, cells were incubated with 500 nM LumiTracker Mito Orange CMTMRos (Lumiprobe) in imaging buffer without fetal bovine serum for 1h. Cells were then washed 3 times with prewarmed imaging buffer prior to fluorescence imaging.

MTS assay

U2OS cells (either transfected or untransfected, $1{\cdot}10^4$ cells per well) were seeded in triplicates in 96-well plates (Nunclon Delta surface, Nunc) and incubated overnight to allow the cells to attach to the surface of the wells. The cells were then incubated with 1 µM **JF_{635}-HTL** for 2 h, washed 3 times and illuminated for 1h at 450 nm (2 $mW{\cdot}cm^{-2}$). Cell viability was determined using an MTS assay (Abcam Inc.) according to the supplier's instructions. Briefly, 20 µL of MTS reagent was added (to each well) to both the non illuminated (control plate) and the illuminated plate. After 1 h 20 min, absorbance at 490 nm was measured on a microplate reader (TECAN Spark).

Microscopy

Widefield imaging was performed at the Advanced Light Microscopy Facility (ALMF, EMBL Heidelberg), on a Nikon Ti-E microscope equipped with a Spectra X light engine (Lumencore) with a 40x objective (CFI P-Apo 40x Lambda/0.95/0.25−0.16) and imaged onto a scientific complementary metal-oxide-semiconductor camera (pco.edge 4.2 CL). The microscope was equipped with a CO_2 and temperature-controllable incubator (home-built, 37°C). A quad bandpass filter cube was used to image Hoechst 33342 (excitation 387/11, emission 447/60), EGFP (excitation 485/20, emission 525/50), LumiTracker Mito Orange CMTMRos (excitation 560/25, emission 593/40) **JF_{635}-HTL/JF_{630}-HTL** (excitation 650/13, emission 692/40). Photoswitching was induced by illumination for 10 minutes (450 nm, 2 $mW{\cdot}cm^{-2}$) using the custom-made LED box described above.

Photoactivation of single cells and cycles were performed at the Advanced Light Microscopy Facility (ALMF, EMBL Heidelberg), on a Stellaris 8 confocal point scanning microscope (Leica Microsystems GmbH, Wetzlar Germany), equipped with a DMi8 inverted microscope stand, a CO_2 and temperature-controllable incubator (home-built, 37°C) and a 20x objective (HC PL APO CS2 20x/0.75 IMM; or HC PL APO CS2 20x/0.75 air for fatigue resistance measurement during photoswitching cycles). Image signal was detected with HyD S detectors for **JF_{635}-HTL** (excitation 635 nm, emission 642-712 nm range). Photoswitching was induced directly from the microscope by using a white light laser (440-790 nm, pulsed 80 Mhz) at 450 nm for 6 minutes (1.6 $\mu W{\cdot}cm^{-2}$) for the ROI (i.e. region of interest = single nucleus) or the full field of view (for fatigue resistance measurements).

Widefield and Confocal Image analysis

Statistical analysis as well as curve fitting was performed using GraphPad (Prism). All images were processed with ImageJ/Fiji and macros written therein unless otherwise stated.[19, 20] Segmentation of the nucleus was done in ImageJ/Fiji. Segmentation of actin was done using the Cellpose plugin for ImageJ/Fiji using the 'cyto2' model.[21] For the segmentation and evaluation of the psHaloTag1a_{635} performance in mitochondria, Arif Khan of the bioimage analysis support team at EMBL wrote a specific Python script, which is based on cell segmentation of the cytosolic EGFP signal using a model trained on top of Cellpose model 'cyto2'. The resulting cell labels were matched across images. The fluorescence intensity change F/F_0 was determined for each cell. Dead cells were excluded from the analysis. Arif Khan additionally provided a modified code to quantify intensity change in the photoswitching cycles of psHaloTag1a labelled with **JF_{635}-HTL** or **JF_{630}-HTL**, respectively.

To compare the basal brightness of the activated state, the fluorescence intensity of the psHaloTag1$a_{635/630}$ in the ON state was divided by the cytosolic GFP intensity. For actin, the same mask was used to obtain a value for the basal brightness of each cell. In contrast, for the nucleus and mitochondria, a mean EGFP

value per field of view was calculated and the **JF_{635}** signal was divided by this value to account for differences in masks and areas, which led to larger variations.

For co-localization analysis and Pearson correlation coefficient determination, the EGFP channel was used for ROI generation. Each ROI corresponds to a single transfected cell expressing either H2B-psHaloTag1a (nucleus) or TOMM20-psHaloTag1a (mitochondria). The Pearson correlation coefficient was calculated for each ROI in both fluorescent channels (i.e., specific cellular marker channel vs. **JF_{635}-HTL** channel) by using ImageJ/Fiji software and its Analyze-colocalization-coloc2 plugin. Data were obtained from transfected cells appearing in 6-15 fields of view for each experimental condition (n = 50 transfected cells for single labelling to discard possible bleed-through; n=100 transfected cells with both labels).

SMLM image acquisition and analysis

U2OS cells expressing H2B-psHaloTag1a and H2B-HaloTag were incubated with 25 nM **JF_{635b}-HTL** or **JF_{630b}-HTL** for 2 h and washed 5 times with prewarmed imaging buffer. Prior to imaging, HEPES (pH 7.4, final concentration 10 mM) was added.

All imaging experiments were performed on a custom-built single-molecule localization microscope[22] equipped with a 100x objective. The microscope was equipped with an incubator so that the cells could be maintained a 37ºC under ambient atmostphere. Fluorescence emission was collected through a 700/100 nm bandpass filter, and blinking movies were acquired using 4.8 mW of 640 nm laser excitation with an irradiance of 0.21 kW·cm^{-2}. Each region of interest (ROI) contained between one and four cells and imaged for cells expressing psHaloTag1a or HaloTag labelled with **JF_{630}b-HTL** or **JF_{635}b-HTL**.

To examine photoactivation behavior during SMLM acquisition, blinking movies were recorded under defined illumination conditions. Initially, 5000 frames were acquired at an exposure time of 100 ms using 4.8 mW of 640 nm laser excitation. This was followed by the acquisition of an additional 5000 frames under identical conditions with the simultaneous addition of 450 nm illumination (LED box, 0.16 mW·cm^{-2}). Subsequently, two further sequences of 5000 frames each were acquired under the same conditions but without 450 nm illumination. Finally, an additional 5000 frames were acquired with 450 nm illumination, after which imaging was continued on the same ROI under identical conditions in the absence of 450 nm excitation.

Single-molecule events in the raw image data were identified and fitted using 'Picasso: Localize'.[23] The parameters were set as follows. For point spread function (PSF) identification, a box side length of 5 pixels and a minimum net gradient threshold of 3000 were applied to all images. Photon conversion parameters were adjusted according to the camera specifications: the EM gain was set to 1, baseline to 100, the sensitivity (conversion factor, electrons per count) to 0.24, the quantum efficiency to 1, and the pixel size to 106 nm. For PSF fitting, least squares (LQ) Gaussian method was employed.

The localized data generated by Picasso: Localize were subsequently processed using the Filter module of Picasso. Localizations corresponding to individual cells were filtered and exported as CSV files. For image reconstruction, the localized data was rendered in the Render module of Picasso.

References

(1) Wilhelm, J.; Kuhn, S.; Tarnawski, M.; Gotthard, G.; Tunnermann, J.; Tanzer, T.; Karpenko, J.; Mertes, N.; Xue, L.; Uhrig, U.; et al. Kinetic and Structural Characterization of the Self-Labeling Protein Tags HaloTag7, SNAP-tag, and CLIP-tag. *Biochemistry* **2021**, *60* (33), 2560-2575. DOI: 10.1021/acs.biochem.1c00258 From NLM Medline.
(2) Grimm, J. B.; Muthusamy, A. K.; Liang, Y.; Brown, T. A.; Lemon, W. C.; Patel, R.; Lu, R.; Macklin, J. J.; Keller, P. J.; Ji, N.; et al. A general method to fine-tune fluorophores for live-cell and in vivo imaging. *Nat Methods* **2017**, *14* (10), 987-994. DOI: 10.1038/nmeth.4403.
(3) Zheng, Q.; Ayala, A. X.; Chung, I.; Weigel, A. V.; Ranjan, A.; Falco, N.; Grimm, J. B.; Tkachuk, A. N.; Wu, C.; Lippincott-Schwartz, J.; et al. Rational Design of Fluorogenic and Spontaneously Blinking Labels for Super-Resolution Imaging. *ACS Cent Sci* **2019**, *5* (9), 1602-1613. DOI: 10.1021/acscentsci.9b00676.
(4) Deo, C.; Abdelfattah, A. S.; Bhargava, H. K.; Berro, A. J.; Falco, N.; Farrants, H.; Moeyaert, B.; Chupanova, M.; Lavis, L. D.; Schreiter, E. R. The HaloTag as a general scaffold for far-red tunable chemigenetic indicators. *Nat Chem Biol* **2021**, *17* (6), 718-723. DOI: 10.1038/s41589-021-00775-w.
(5) Grimm, J. B.; Tkachuk, A. N.; Xie, L.; Choi, H.; Mohar, B.; Falco, N.; Schaefer, K.; Patel, R.; Zheng, Q.; Liu, Z.; et al. A general method to optimize and functionalize red-shifted rhodamine dyes. *Nat Methods* **2020**, *17* (8), 815-821. DOI: 10.1038/s41592-020-0909-6.
(6) Wang, L.; Tran, M.; D'Este, E.; Roberti, J.; Koch, B.; Xue, L.; Johnsson, K. A general strategy to develop cell permeable and fluorogenic probes for multicolour nanoscopy. *Nature Chemistry* **2019**, *12* (2), 165-172. DOI: 10.1038/s41557-019-0371-1.
(7) Farrants, H.; Shuai, Y.; Lemon, W. C.; Hernandez, C. M.; Yang, S.; Patel, R.; Qiao, G.; Frei, M. S.; Grimm, J. B.; Hanson, T. L.; et al. A modular chemigenetic calcium indicator enables in vivo functional imaging with near-infrared light. *bioRxiv* **2023**. DOI: 10.1101/2023.07.18.549527.
(8) Halavaty, A. S.; Moffat, K. N- and C-Terminal Flanking Regions Modulate Light-Induced Signal Transduction in the LOV2 Domain of the Blue Light Sensor Phototropin 1 from AVena satiVa. *Biochemistry* **2007**, *46*, 14001-14009.
(9) Holland, K. L.; Plutkis, S. E.; Daugird, T. A.; Sau, A.; Grimm, J. B.; English, B. P.; Zheng, Q.; Dave, S.; Rahman, F.; Xie, L.; et al. A series of spontaneously blinking dyes for super-resolution microscopy. *bioRxiv preprint* **2024**, 10.1101/2024.1102.1123.581625.
(10) OpenWetWare. *Lidstrom:Autoinduction Media*. 2021. https://openwetware.org/wiki/Lidstrom:Autoinduction_Media (accessed November 4, 2024).
(11) Mirdita, M.; Schütze, K.; Moriwaki, Y.; Heo, L.; Ovchinnikov, S.; Steinegger, M. ColabFold - Making protein folding accessible to all. *bioRxiv* **2022**. DOI: 10.1101/2021.08.15.456425.
(12) Kabsch, W. XDS. *Acta Crystallogr D Biol Crystallogr* **2010**, *66* (Pt 2), 125-132. DOI: 10.1107/S0907444909047337 From NLM Medline.
(13) Vonrhein, C.; Tickle, I.; Flensburg, C.; Keller, P.; Paciorek, W.; Sharff, A.; Bricogne, G. Advances in automated data analysis and processing within autoPROC, combined with imrpoved characterisation, mitigation and visualisation of the anisotropy of diffraction limits using STARANISO. *Acta Crystallographica Section A: Foundations and Advances* **2018**, *74*, a360.
(14) Kang, M. G.; Lee, H.; Kim, B. H.; Dunbayev, Y.; Seo, J. K.; Lee, C.; Rhee, H. W. Structure-guided synthesis of a protein-based fluorescent sensor for alkyl halides. *Chem Commun (Camb)* **2017**, *53* (66), 9226-9229. DOI: 10.1039/c7cc03714g.
(15) McCoy, A. J. Solving structures of protein complexes by molecular replacement with Phaser. *Acta Crystallogr D Biol Crystallogr* **2007**, *63* (Pt 1), 32-41. DOI: 10.1107/S0907444906045975.
(16) Adams, P. D.; Afonine, P. V.; Bunkoczi, G.; Chen, V. B.; Davis, I. W.; Echols, N.; Headd, J. J.; Hung, L. W.; Kapral, G. J.; Grosse-Kunstleve, R. W.; et al. PHENIX: a comprehensive Python-based system for macromolecular structure solution. *Acta Crystallogr D Biol Crystallogr* **2010**, *66* (Pt 2), 213-221. DOI: 10.1107/S0907444909052925.
(17) Emsley, P.; Lohkamp, B.; Scott, W. G.; Cowtan, K. Features and development of Coot. *Acta Crystallogr D Biol Crystallogr* **2010**, *66* (Pt 4), 486-501. DOI: 10.1107/S0907444910007493.
(18) Afonine, P. V.; Grosse-Kunstleve, R. W.; Echols, N.; Headd, J. J.; Moriarty, N. W.; Mustyakimov, M.; Terwilliger, T. C.; Urzhumtsev, A.; Zwart, P. H.; Adams, P. D. Towards automated crystallographic structure

refinement with phenix.refine. *Acta Crystallogr D Biol Crystallogr* **2012**, *68* (Pt 4), 352-367. DOI: 10.1107/S0907444912001308.
(19) Schindelin, J.; Arganda-Carreras, I.; Frise, E.; Kaynig, V.; Longair, M.; Pietzsch, T.; Preibisch, S.; Rueden, C.; Saalfeld, S.; Schmid, B.; et al. Fiji: an open-source platform for biological-image analysis. *Nature Methods* **2012**, *9* (7), 676-682. DOI: 10.1038/nmeth.2019.
(20) Rueden, C. T.; Schindelin, J.; Hiner, M. C.; DeZonia, B. E.; Walter, A. E.; Arena, E. T.; Eliceiri, K. W. ImageJ2: ImageJ for the next generation of scientific image data. *BMC Bioinformatics* **2017**, *18* (1). DOI: 10.1186/s12859-017-1934-z.
(21) Stringer, C.; Wang, T.; Michaelos, M.; Pachitariu, M. Cellpose: a generalist algorithm for cellular segmentation. *Nature Methods* **2020**, *18* (1), 100-106. DOI: 10.1038/s41592-020-01018-x.
(22) Power, R. M.; Tschanz, A.; Zimmermann, T.; Ries, J. Build and operation of a custom 3D, multicolor, single-molecule localization microscope. *Nat Protoc* **2024**, *19* (8), 2467-2525. DOI: 10.1038/s41596-024-00989-x From NLM Medline.
(23) Schnitzbauer, J.; Strauss, M. T.; Schlichthaerle, T.; Schueder, F.; Jungmann, R. Super-resolution microscopy with DNA-PAINT. *Nat Protoc* **2017**, *12* (6), 1198-1228. DOI: 10.1038/nprot.2017.024 From NLM Medline.
